# Effects of pregabalin on neurobehavior in an adult male rat model of PTSD

**DOI:** 10.1371/journal.pone.0209494

**Published:** 2018-12-31

**Authors:** Debra A. Valdivieso, Thomas G. Baughan, Ursuline M. Canavati, Allison M. Rey, Cristal L. Trotter, Destynni R. Burrell, John E. Buonora, Tomás Eduardo Ceremuga

**Affiliations:** US Army Graduate Program in Anesthesia Nursing, Academy of Health Sciences, AMEDDC&S, Fort Sam Houston, Texas, United States of America; Technion Israel Institute of Technology, ISRAEL

## Abstract

Posttraumatic stress disorder (PTSD) can be a very debilitating condition. Effective approaches to prevent and treat PTSD are important areas of basic science research. Pregabalin (PGB), a gabapentinoid derivative of γ-aminobutyric acid, possesses the potential to positively affect neurobehavioral changes associated with PTSD. Using a rodent model of PTSD, the aims of this study were to determine the effects of PGB as a possible prevention for the development of PTSD-like symptoms and its use as a possible treatment. A prospective, experimental, between groups design was used in conjunction with a three-day restraint/shock PTSD stress model. Sixty rats were randomly assigned between two groups, non-stressed and stressed (PTSD). Each of the main two groups was then randomly assigned into six experimental groups: control vehicle, control PGB, control naïve, PTSD vehicle, PTSD Pre-PGB (prophylactic), PTSD Post-PGB (non-prophylactic). The neurobehavioral components of PTSD were evaluated using the elevated plus maze (EPM), Morris water maze (MWM), and forced swim test (FST). Pregabalin administered 24 hours before the initial PTSD event or for 10 days following the last PTSD stress event did not statistically improve mean open arm exploration on the EPM, spatial memory, and learning in the MWM or behavioral despair measured by the FST (p > 0.05).

## Introduction

Posttraumatic stress disorder (PTSD) has been sequalae of combat and traumatic event survivors for centuries. The earliest recording of PTSD-like symptoms can be traced to 490 BCE, by the Greek historian Herodotus [1]. The risk of PTSD is of particular interest to the men and women serving in the military, as they are exposed to combat events which can be triggers for the development of PTSD. Between 2000 and August 2015, over 177,000 service members have been diagnosed with PTSD [2]. Longitudinal research on PTSD demonstrates that it can have a delayed onset of symptoms and can become a chronic mental health condition [3]. Data from the 2001–2013 Millennium Cohort Study indicates that 47% of respondents who were found to have a positive score for PTSD during their initial survey were still positive three years later [4]. PTSD resulting from traumatic events is not limited to military service members. Results from the 2013 National Epidemiologic Survey on Alcohol and Related Conditions-III revealed that traumatic events had directly impacted an estimated 4.7% of Americans in 2012–2013, which translates to a 6.1 ± 0.21% lifetime prevalence [5]. When comparing the epidemiology of PTSD over a decade, the prevalence of the disorder remained relatively constant from the 1990s to 2000. However, when evaluating available data from 2003 to 2013, a disturbing trend was noted. There has been a steady increase in the prevalence of PTSD in both yearly numbers of disorder diagnosis and calculated lifetime prevalence [6]. The financial impact of PTSD on the United States healthcare economy remains significant. A diagnosis of PTSD adds, an additional average annual healthcare cost of $763 for Medicaid patients and $936 for private pay patients [7].

The long-term consequences of traumatic stress on both physical and psychological well-being are numerous and complex [8]. Emotional reactions associated with trauma or life-threatening events are common. However, if these reactions persist or gradually become worse over time, they become the basis for PTSD. Common symptoms associated with PTSD include the following: intruding thoughts, avoidance of reminders of the event, re-experiencing or reliving the event through flashbacks, strong negative reactions to seemingly innocuous triggers, nightmares, hyperarousal, and negative feelings [9]. Along with these classic symptoms of PTSD there is also a robust association of anxiety and depression among this group of patients [10, 11]. Physiologically, PTSD affects structural changes in the brain as well as neurochemical alterations and imbalances [12]. Reductions in hippocampal volume and an increase in amygdala activity have been reported [13–15]. The hippocampus has long been implicated in associative learning and spatial memory [14]. Amygdala activity has been attributed to contextual fear memory and potentiating such episodes into long-term memories of the event [16].

There is a substantial need for further research in PTSD; the opportunity for pharmacological and nutraceutical interventions in PTSD is one such exciting avenue to explore. Historically rodents have been used for basic neuroscience research. Decades of well-validated experimental instruments are available which aid in the translational interpretation of rodent data to the human physiology and neurochemical changes associated with anxiety, depression, spatial learning, and memory formation. Dysregulation of the excitatory neurotransmitter glutamate has been implicated in the hallmark features of stress-related psychiatric disorders including PTSD [17].

Numerous pharmacological and nutraceutical compounds have been tested in an attempt to alleviate the symptoms of PTSD [18]. One such medication that has shown promise in clinical applications is pregabalin. Pregabalin (PGB), a gabapentinoid derivative of γ-aminobutyric acid, possesses the potential to positively affect neurobehavioral changes associated with PTSD. Baniasadi *et al*., found that pregabalin used as an adjunct to traditional treatments, effectively reduced the severity of PTSD symptoms in patients diagnosed with chronic PTSD [19]. This study of 37 adult male patients specifically examined a post-exposure treatment model.

Pregabalin is an analog of the major inhibitory neurotransmitter GABA, but it is functionally different. Pregabalin does not bind to GABA_**A**_ or GABA_**B**_ receptors and is not converted metabolically to GABA or to a GABA agonist. Pregabalin’s pharmacological properties are the result of presynaptic binding to the alpha-2-delta subunit of voltage-gated calcium channels and has also been shown to reduce glutamate release at cortical synapses.

This binding has been shown to reduce depolarization-induced calcium influx at nerve terminals, which reduces the release of several excitatory neurotransmitters such as glutamate and norepinephrine. Studies of animal models have shown that the modulation of neurotransmitter release is the most likely mechanism of its analgesic, anticonvulsant, and anxiolytic effects [20].

Recognizing that PTSD is the result of precipitating traumatic event(s) which often leads to or necessitates follow-up medical care, a thorough understanding of the physiology of the beginning of PTSD, and potential pathways of relief from PTSD symptoms is vitally important. An innovative approach to the prevention of the neurobiological development of PTSD may include effective prophylactic treatment for those individuals at high risk to experience traumatic events.

The aim of this study was to evaluate the effectiveness of PGB as a prophylactic treatment and as a post-traumatic stress event treatment, utilizing well-established rodent neurobehavioral models of anxiety, locomotion, memory, and despair.

## Material and methods

### Animals

The study was performed using a homogenous (sex and ancestry) sample of adult male Sprague-Dawley rats (Envigo, Houston, TX) with purchase weights of 200–249 grams. Housing was in groups of two in polycarbonate Sealsafe Plus GR900 cages (Tecniplast, West Chester, PA) lined with bedding and containing environmental enrichment. The rats underwent an adaptation period of seven days in a temperature-controlled environment (20–26°C, 30–70% humidity) with a reverse light-dark cycle, 12 hours of light from 0001 to 1200 and 12 hours of darkness from 1200 to 2400. All rats had free access to food and water. Water was supplied as a liquid and in hydrogel form (ClearH20, Westbrook ME). Handling of the animals was only for weighing, drug administration, and cleaning of cages. After the acclimation period, body weight, food, and water consumption were measured daily up to the first day of neurobehavioral testing. Baseline body weights were measured, and there was not a significant or clinical difference in baseline body weights between the control and experimental groups. Rats were randomly assigned to the non-stressed group or the 3-day restraint shock PTSD group. Following the initial division into two main groups, each main group was then divided into three subgroups. The three non-stressed subgroups consisted of the following: control vehicle, control PGB, and control naïve. The three stressed groups consisted of these: PTSD-vehicle, PTSD Pre-PGB, and PTSD Post-PGB. Each animal received a subcutaneous injection of either saline (vehicle) or pregabalin 10mg/kg in saline. The volumes of all subcutaneous injections totaled one mL and were administered twice per day (0700 hrs and 1900 hrs). The pre-PGB animals were given their first dose 24 hours before the 3-day restraint/shock stress model began, while the post-PGB animals were given their first dose of PGB following the third day of the restraint/shock. Pregabalin injections continued daily throughout the remainder of the experimental period. Pregabalin was generously gifted by Pfizer (New York City, NY) through Pfizer’s Compound Transfer Program. All animal experiments were performed in accordance with the Association for Assessment and Accreditation of Laboratory Animal Care (AAALAC International) directives after obtaining the approval from the US Army Institute of Surgical Research Institutional Animal Care and Use Committee (IACUC). The IACUC at the US Army Institute of Surgical Research, Fort Sam Houston approved of this research protocol. Protocol #A-15-037. Isoflurane was used as the anesthetic before animal sacrifice.

Based on an a priori power analysis and consultation with neurobehavioral scientists regarding replacement, reduction, and refinement in optimizing animal use, a sample of sixty rats was used for the current investigation. The determination of the effect size was based on previous studies that have investigated various compounds for their effects on PTSD and anxiety [21–25]. Use of the software program, G-Power 3.1, with a large standardized effect size of *F* = 0.45, power of 0.8, and α = 0.05, determined a total sample size of 60 rats or 10 rats per group [26].

### Instruments

#### Three-day restraint/tail shock PTSD stress model

Stress exposure consisted of one two-hour session per day of immobilization and tail-shocks. Rats were restrained in a transparent acrylic glass tube and 40 electric shocks (2 mA, 3 s duration) were applied at random intervals (140–180 s). Commercial hardware and software controlled the timing and amplitude of the stimulus (Precision Animal Shocker, Coulbourn Instruments, Columbus, Ohio, USA). Rats were stressed for three consecutive days at approximately the same time each day. It has been demonstrated that repeated stress sessions are more effective than a single stress session in producing physiological and behavioral abnormalities such as acoustic startle response and reduced body weight consistent with PTSD [27]. After the 3-day restraint/tail shock stress model was completed, all rats except the control naïve rats were given subcutaneous injections based on their body weight twice daily for a period of ten days. This allowed for the development of PTSD-like symptoms, as demonstrated by previous studies [27].

#### Elevated plus maze (EPM)

The EPM is a widely utilized and validated instrument to measure anxiety in the rodent model [28]. The EPM is made entirely of Plexiglas and consists of a set of opposing open arms and a set of opposing closed arms, which are each 50 cm in length and 10 cm wide. The two open arms are lined with 1 cm high Plexiglas on the sides to avoid falls. The maze is placed 50 centimeters above the floor and surrounded by screens to minimize any room cues or influences. The walking surface of the EPM is Plexiglas to avoid excess stimulation and provide a waterproof surface when exposed to urine and feces. The maze is in the shape of a cross (+), with the intersection of the four arms measuring 10 x 10 cm. Each experimental session was recorded for five minutes by the AnyMaze software where mean speed (centimeters per second), mean time mobile (seconds), and open-arm time ratio (percent) were the main data points investigated. Open arm time ratio was calculated by taking the time spent in the open arm divided by the total recorded time and multiplying by 100 to get a percentage. An increase in the percentage of time spent in the open arms reflects an anxiolytic effect [28]. The EPM was cleansed between each testing with non-fragrant soap and water and thoroughly dried. After the EPM test, animals were taken to the Morris water maze room.

#### Morris water maze (MWM)

After completion of the five-minute EPM, the animals were taken to the MWM for testing. The water maze task has been most extensively used to investigate specific aspects of spatial memory. This task is based upon the premise that animals have evolved an optimal strategy to explore their environment and escape from the water with a minimum amount of effort—i.e., swimming the shortest distance possible [29]. The time it takes a rat to find a hidden platform in a water pool after previous exposure to the setup, using only available external cues, is determined as a measure of spatial memory [30]. The water maze test, as described previously with minor modifications, was used on all animals [31, 32]. The setup consisted of a circular tank (190 cm in diameter) filled with water (up to 30cm deep; temperature: 22 ± 2 °C) and made opaque by the addition of a non-toxic dye. The pool was divided into four zones. A platform (18 cm × 18 cm) was submerged 2 cm below the water surface in zone 1. The pool was placed in a small room with external cues, kept constant throughout the experiments. The data were recorded and analyzed with an overhead video-camera connected to the AnyMaze software. For training, all rats were exposed six times per day to the setup for two consecutive days, for a total of 12 trials. Each rat was given 60 seconds to randomly explore the water maze. If the animal did not find the platform in this period by chance, it was guided to it and allowed to remain there for 10 seconds to familiarize itself with the location of the platform relative to the visual cues in the maze. Formal data recording consisted of the probe test and commenced on the third day of MWM exposure. The probe test involved removing the platform and the rats undergoing a single trial of 60 seconds (probe trial). The percentage of time spent in each zone and the area where the platform was previously located was recorded. After the end of the probe test, animals were taken to the forced swim test room.

#### Forced swim test (FST)

After testing in the MWM, animals were carried to a separate room and evaluated in the FST. The FST is a validated test of behavioral despair in the rodent model [33]. In two sessions separated by 24 hours, rats were forced to swim in a narrow cylinder from which they cannot escape. Transparent glass cylinders (20.32 cm diameter × 71.12 cm high) contained water (25°C ± 2°C) at a depth sufficient to prevent the rat from touching the bottom of the cylinder. The first session, lasting 15 minutes, was the training session and was conducted 24 hours prior to data collection and without behavioral recording. The initial 15-minute session was conducted to habituate and acclimate the rats to the test situation, thereby providing a stable, high level of immobile behavior during the five-minute recorded test session 24 hours later. The AnyMaze software was used to video record the five-minute test session. The de-identified videos were independently reviewed by three investigators and the amount of time the rats were immobile was the mean of the three investigators’ times. The measurement of the duration of immobility when rodents are exposed to an inescapable situation (FST) is a reflection of behavioral despair or learned helplessness. In this rodent model of depression, the longer the duration of immobility, the greater the behavioral despair or depression. The amount of immobility time was statistically analyzed between groups.

#### Fecal pellet output (FPO)

Multiple species, including rodents, demonstrate a stress response by changes in colonic motor activity. Increased stool output and transit speed are reliable measures of autonomic system modulation of colonic motility in both humans and rodents [34, 35]. The FST fecal pellet output (defecation pellets) were measured and statistically analyzed between groups.

### Statistical analysis

For this design, a one-way ANOVA was conducted for each of the outcome variables. All assumptions were examined including homogeneity of error variances (via the Levine test) and normality. The eta-squared (η^2^) effect size is reported. Though interpreting and casting judgment as to what constitutes a small/medium/large effect size is context-dependent using Cohen’s taxonomy, .01/.059/138 is small/medium/large [36]. All outliers and data anomalies were examined and addressed accordingly (e.g., transformations, nonparametric options, etc.). In the event of a significant result (α = .05), post-hoc tests (e.g., Tukey’s HSD) were performed. A separate analysis was conducted using ANCOVA comparing the groups while controlling for days, total body weight, water intake, and food intake variables. As stated in the results, there were four cases with replacements; therefore, for the ANCOVA, the sample size was n = 56. The homogeneity of regression assumption was tested (i.e., testing the group x covariate interaction). The Sidak multiple comparison procedure test (MCP) was used to test pairwise comparisons for significance.

## Results

During the three-day restraint/tail shock PTSD stress model, three rats from the PTSD-drug pretreatment group expired and an additional rat from the PTSD-drug post-treatment group expired during the FST acclimation test. Replacement rats were added to the next cohort of experiments to maintain the sample size of 10 rats per group.

### Total body weight

**Fig 1:**
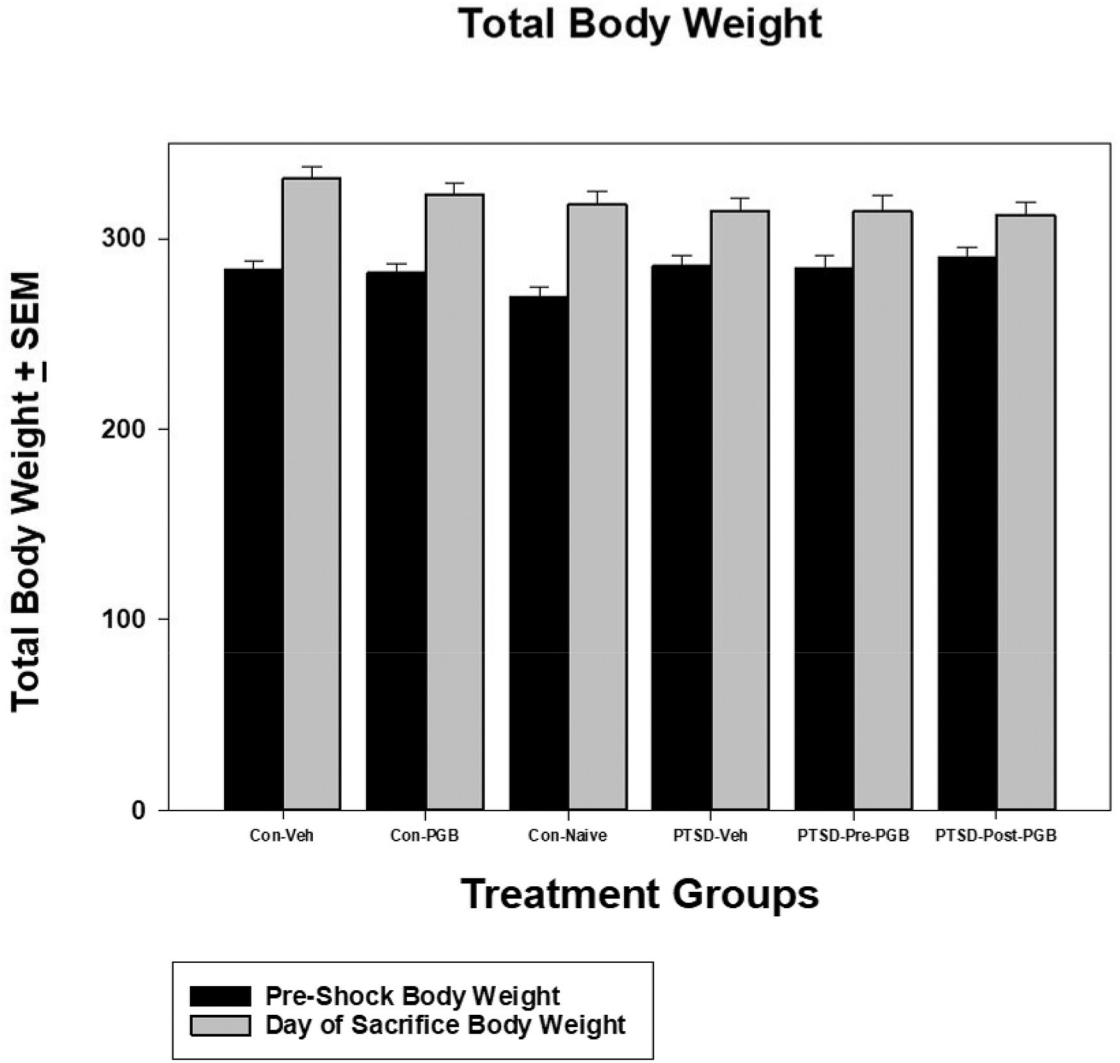
ANCOVA for total body weight. Each of the main two groups (Stressed and Non-stressed) had three subgroups: non-stressed: control vehicle, control PGB, control naïve; and stressed: Posttraumatic stress disorder (PTSD) vehicle, PTSD Pre-PGB (prophylactic), PTSD Post-PGB (non-prophylactic). Mean total body weight is reported with the standard error of the mean (SEM).

For the 6 x 2 (group x time) design ANCOVA with days as a covariate (*n* = 56, given missing data for *n* = 4 cases on the covariate), there was a significant 2-way interaction: *F*(5, 49) = 6.50, *p* < .001 (η^2^ = .399). The nature of the interaction is such that at the pre-shock stage the PTSD-post-treatment group has the highest mean (*M* = 289.7) and the control-naïve group the lowest (*M* = 269.23) whereas for the subsequent day of sacrifice outcome the control-vehicle group has the highest mean (*M* = 331.34) and the PTSD-post-treatment the lowest mean (*M* = 311.97). The simple effects for group (i.e., comparing the groups at each wave of data collection, controlling for days) were not significant for both preshock: *F*(5, 49) = 1.86, *p* = .119 (η^2^ = .16) or for day of sacrifice: *F*(5, 49) = 1.19, *p* = .329 (η^2^ = .108). When analyzing the simple effect for time, each of the groups has a significant increase in weight across time with the largest mean differences when adjusting for the covariate (*M*_*d*_) obtained for the control-naïve group (*M*_*d*_ = 48.94) and the smallest mean difference for the PTSD-post-treatment group (*M*_*d*_ = 22. 27) (Fig 1).

### Total water intake weight

**Fig 2:**
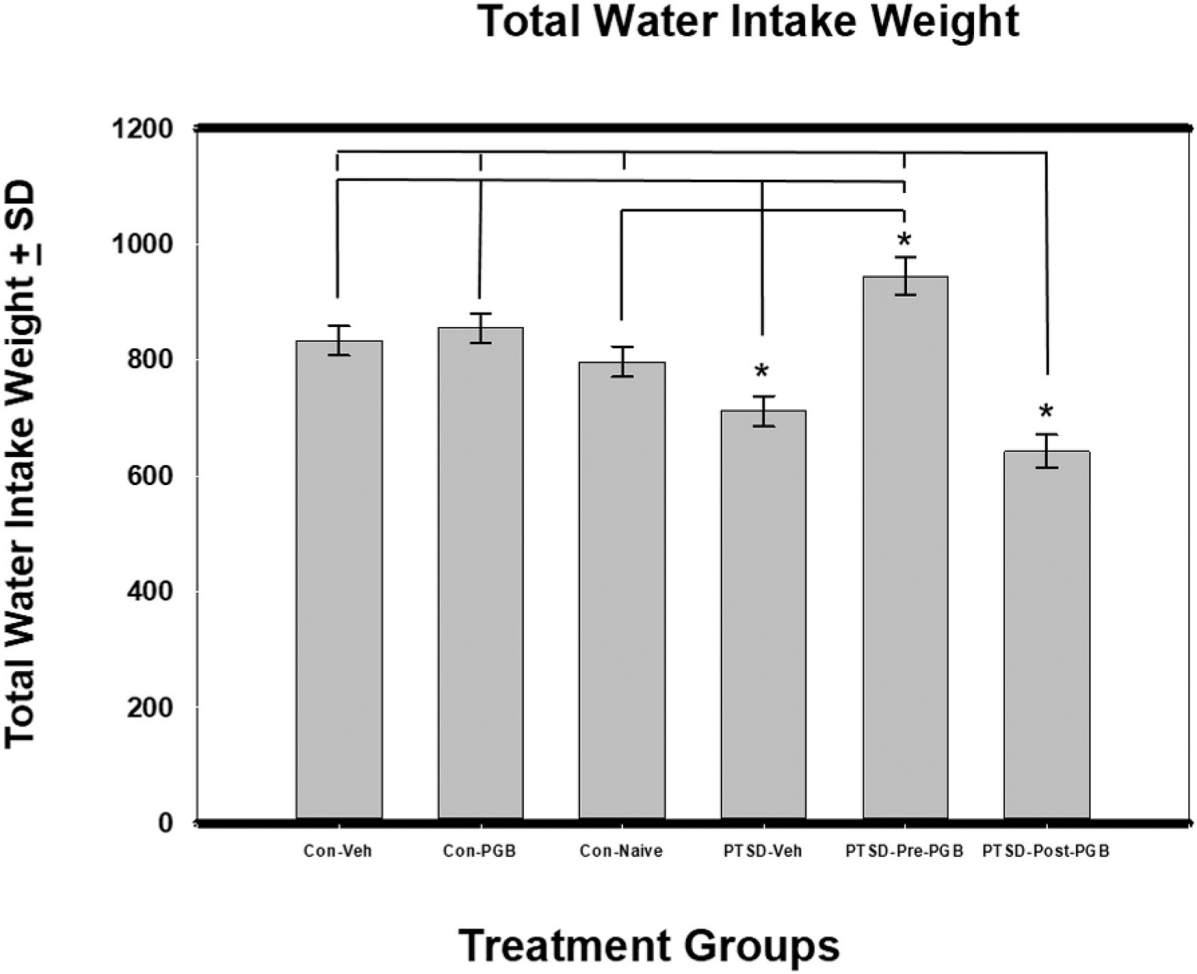
ANCOVA for total water intake weight. Each of the main two groups (Stressed and Non-stressed) had three subgroups: non-stressed: control vehicle, control PGB, control naïve; and stressed: Posttraumatic stress disorder (PTSD) vehicle, PTSD Pre-PGB (prophylactic), PTSD Post-PGB (non-prophylactic). Mean total water intake weight is reported with the SEM. Asterisks denote statistical significance at *p* < .05. PTSD-Post-PGB was significantly less than PTSD-Pre-PGB and all three control groups. PTSD Veh was significantly lower than Con-Veh, Con-PGB, and PTSD-Pre-PGB. PTSD-Pre-PGB was significantly higher than Con-Naïve.

When controlling for days via one-way ANCOVA, there was a significant difference between the six groups: *F*(5, 54) = 13.47, *p* < .001 (η^2^ = .579) and the covariate was significant (*p* < .001, η^2^ = .363). The PTSD Pre-PGB group had the highest mean (*M* = 945.04) and PTSD vehicle the lowest (*M* = 711.75). Per the Sidak test (MCP pairwise tests based on the adjusted means) the PTSD vehicle group had a significantly lower mean than all the groups except for control naïve (*p* = .365) and PTSD Post-PGB group (*p* = .770). The PTSD Post-PGB group had a significantly lower mean than all the groups except PTSD vehicle (as mentioned previously, *p* = .770). The PTSD Pre-PGB group had a significantly higher mean than the control naïve group (*p* = .018) (Fig 2).

### Total food intake weight

**Fig 3:**
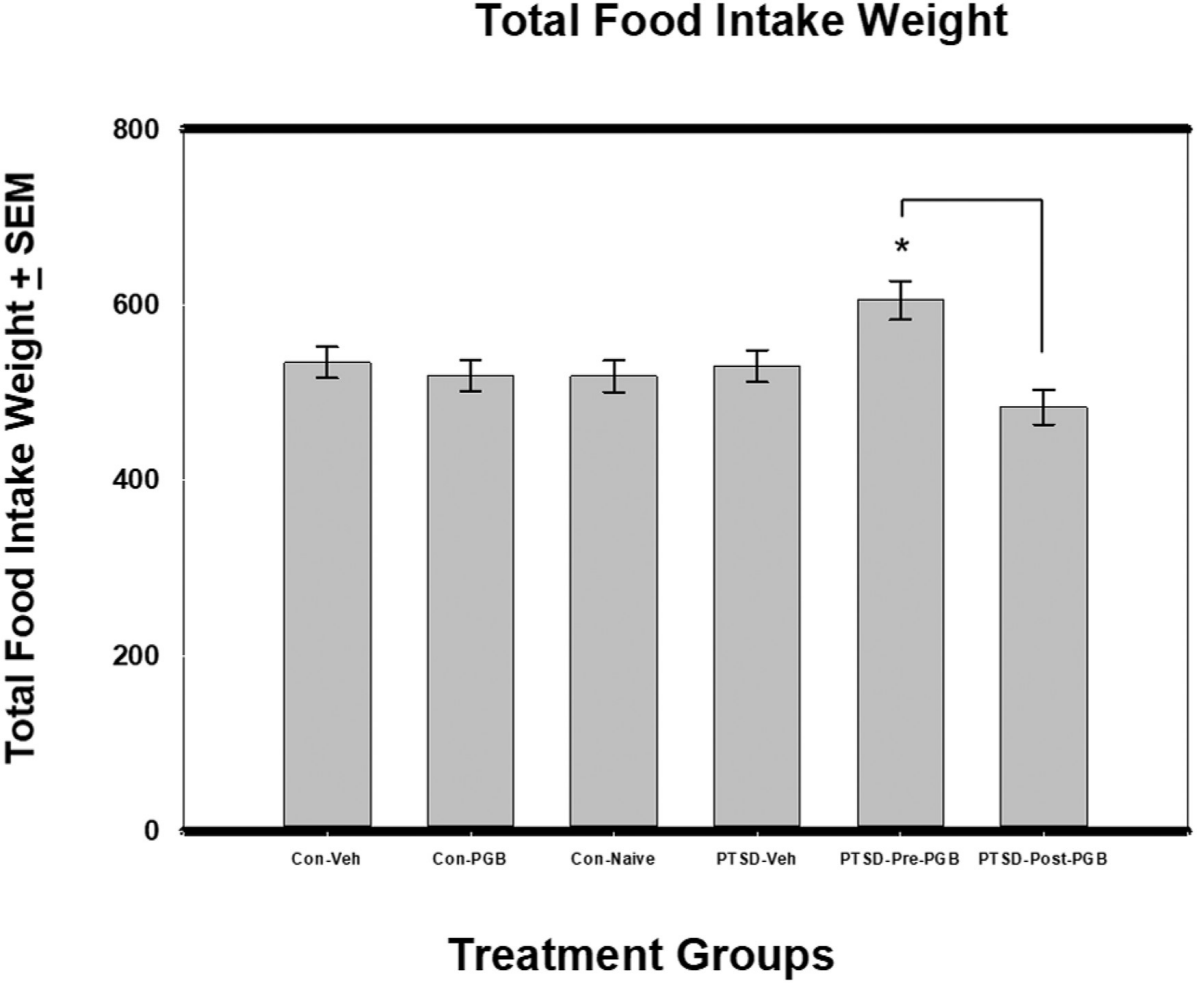
ANCOVA for total food intake weight. Each of the main two groups (Stressed and Non-stressed) had three subgroups: non-stressed: control vehicle, control PGB, control naïve, and stressed: Posttraumatic stress disorder (PTSD) vehicle, PTSD Pre-PGB (prophylactic), PTSD Post-PGB (non-prophylactic). Mean total food intake weight is reported with the SEM. Asterisks denote statistical significance at *p* < .05. PTSD-Pre-PGB was significantly higher than the PTSD-Post-PGB.

For the Total Food Intake Weight outcome, when controlling for days via one-way ANCOVA, there was a significant difference between the six groups: *F*(5, 54) = 3.17, *p* = .015 (η^2^ = .244) and the covariate was significant (*p* < .001, η^2^ = .411). The PTSD Pre-PGB group had the highest mean (*M* = 605.11) and PTSD Post-PGB the lowest (*M* = 483.4) (Fig 3). Per the Sidak test, the only significant pairwise difference (based on the adjusted means) was between PTSD Pre-PGB (higher mean) and PTSD Post-PGB (*p* = .005).

### Elevated plus maze

**Fig 4:**
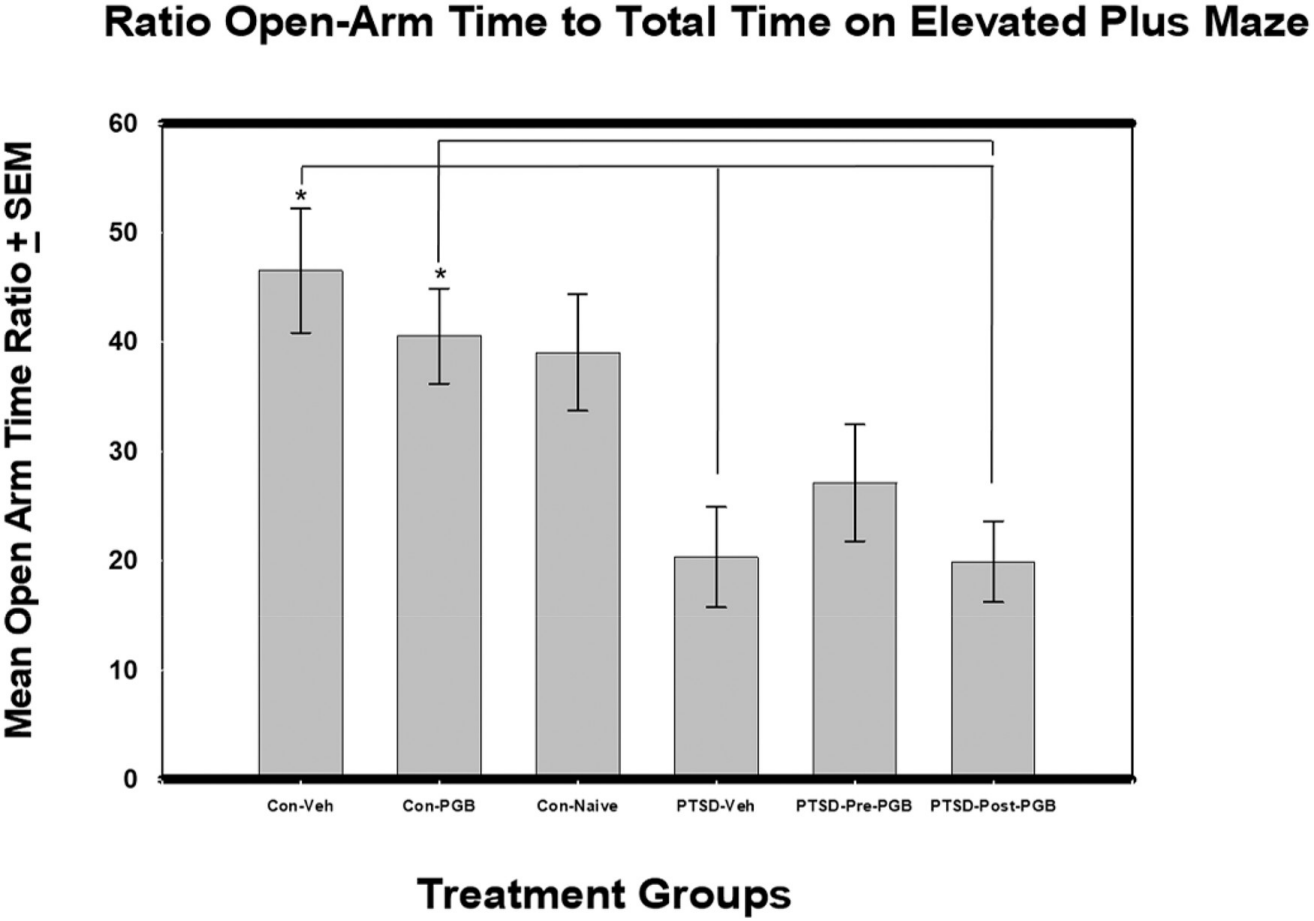
ANOVA of Mean Open-Arm Time Ratio (percent) from Elevated Plus-Maze (EPM) and Standard Error of the Mean (SEM). Each of the main two groups (Stressed and Non-stressed) had three subgroups: non-stressed: control vehicle, control PGB, control naïve, and stressed: Posttraumatic stress disorder (PTSD) vehicle, PTSD Pre-PGB (prophylactic), PTSD Post-PGB (non-prophylactic). Asterisks denote statistical significance at *p* < .05. PTSD vehicle and PTSD Post-PGB post-treatment had significantly lower mean open arm time ratio when compared to control. PTSD Post-PGB had significantly lower mean open arm time ratio when compared to Control PGB.

No significant differences were found between the groups pertaining to mean: mobile episodes, time mobile, max speed, or open arm time. For the distance and the mean speed outcome, there was a significant difference between the six groups: *F*(5, 54) = 2.43, *p* = .047 (η^2^ = .184) and *F*(5, 54) = 2.44, *p* = .046 (η^2^ = .184) respectively. The control vehicle group had the highest mean distance (*M* = 14.0) and PTSD Post-PGB the lowest (*M* = 9.92). The control vehicle group also had the highest mean speed (*M* = .0467) and PTSD Post-PGB the lowest (*M* = .0329). The Tukey HSD post-hoc test indicated there were no significant pairwise differences for either mean distance or speed. For the time freezing outcome, there was a significant difference between the six groups: *F*(5, 54) = 4.85, *p* = .001 (η^2^ = .310). The PTSD Pre-PGB group had the highest mean (*M* = 42.9) and control naive the lowest (*M* = 8.71). The Games-Howell (given violation of the homogeneity of variance assumption) post-hoc test indicated there were no significant pairwise differences. For the open arm entries outcome, there was a significant difference between the six groups: *F*(5, 54) = 3.03, *p* = .018 (η^2^ = .219). The control-naive group had the highest mean (*M* = 10.4) and the PTSD Post-PGB group the lowest (*M* = 4.2). Per the Tukey HSD post hoc test, there were no significant pairwise differences. Analysis of the ratio of open arm time versus total time spent in the elevated plus maze revealed statistically significant increases between the six groups: *F*(5, 54) = 5.36, *p* < .001 (η^2^ = .332). The control vehicle group had the highest mean times (*M* = 46.53), and PTSD Post-PGB group had the lowest (*M* = 19.93). Per the Tukey HSD post-hoc tests, the control vehicle group had a significantly higher mean than the PTSD vehicle and PTSD Post-PGB groups (Fig 4).

### Morris water maze

**Fig 5:**
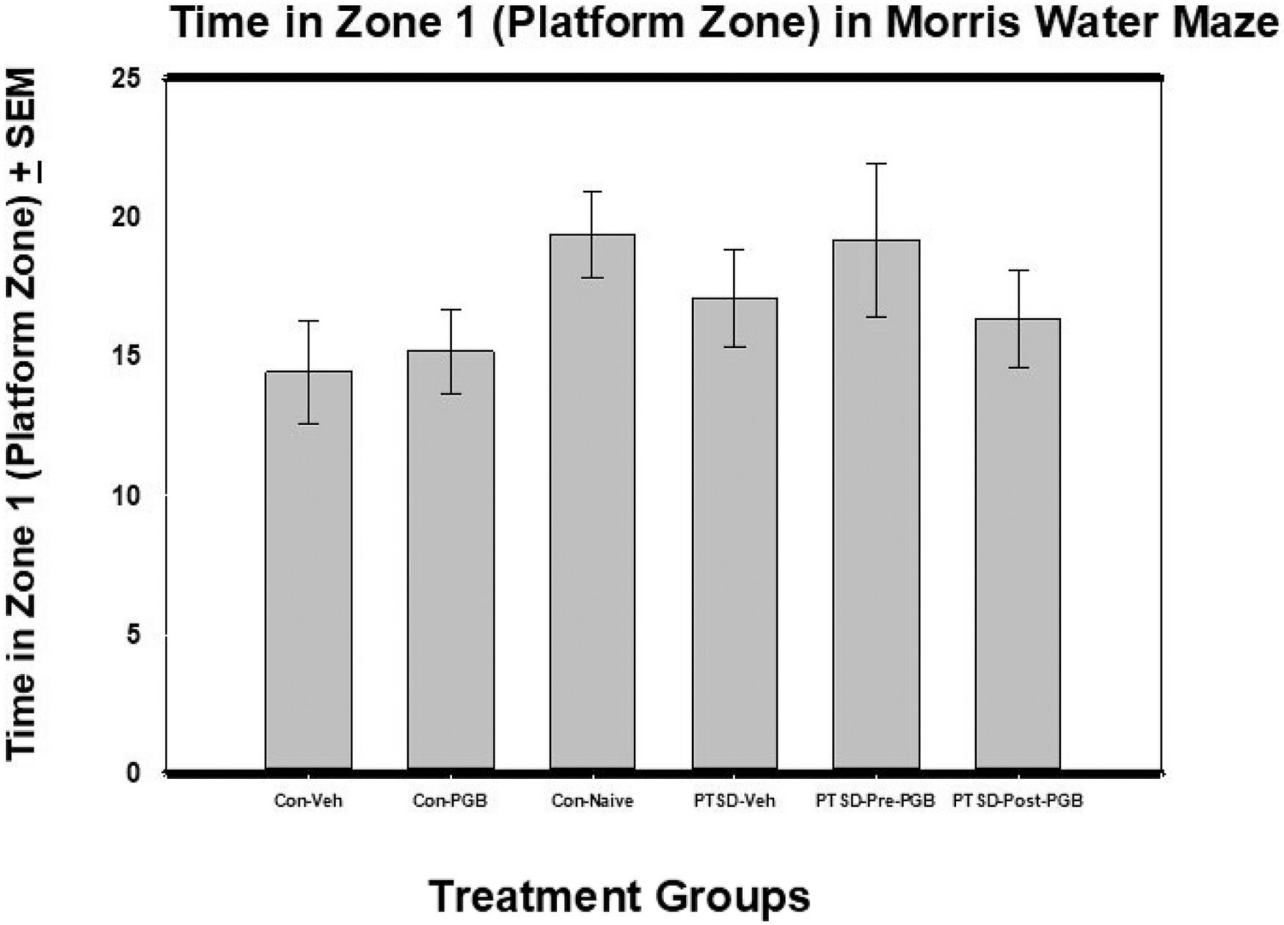
ANOVA of Time in Zone1 (Platform Zone) from Morris water maze (MWM) and Standard Error of the Mean (SEM). Each of the main two groups (Stressed and Non-stressed) had three subgroups: non-stressed: control vehicle, control PGB, control naïve; and stressed: Posttraumatic stress disorder (PTSD) vehicle, PTSD Pre-PGB (prophylactic), PTSD Post-PGB (non-prophylactic). Mean total body weight is reported with the standard error of the mean (SEM). There was not a significant difference between the six groups.

In the Morris water maze test, there were no statistically significant differences found between groups when comparing distance traveled, mean speed, max speed, zone 1 distance traveled, zone 1 latency to first entry, zone 1 max speed, and entries to the platform area in zone 1 (the platform zone). For the Time in Zone1 (Platform Zone) Time outcome, there was not a significant difference between the six groups: *F*(5, 54) = 1.14, *p* = .353 (η^2^ = .095). Though not significant, the control naive and the PTSD Pre-PGB groups had the highest mean times, *M* = 19.37 and 19.18 respectively and the control vehicle group the lowest (*M* = 14.46) (Fig 5).

### Forced swim test

**Fig 6:**
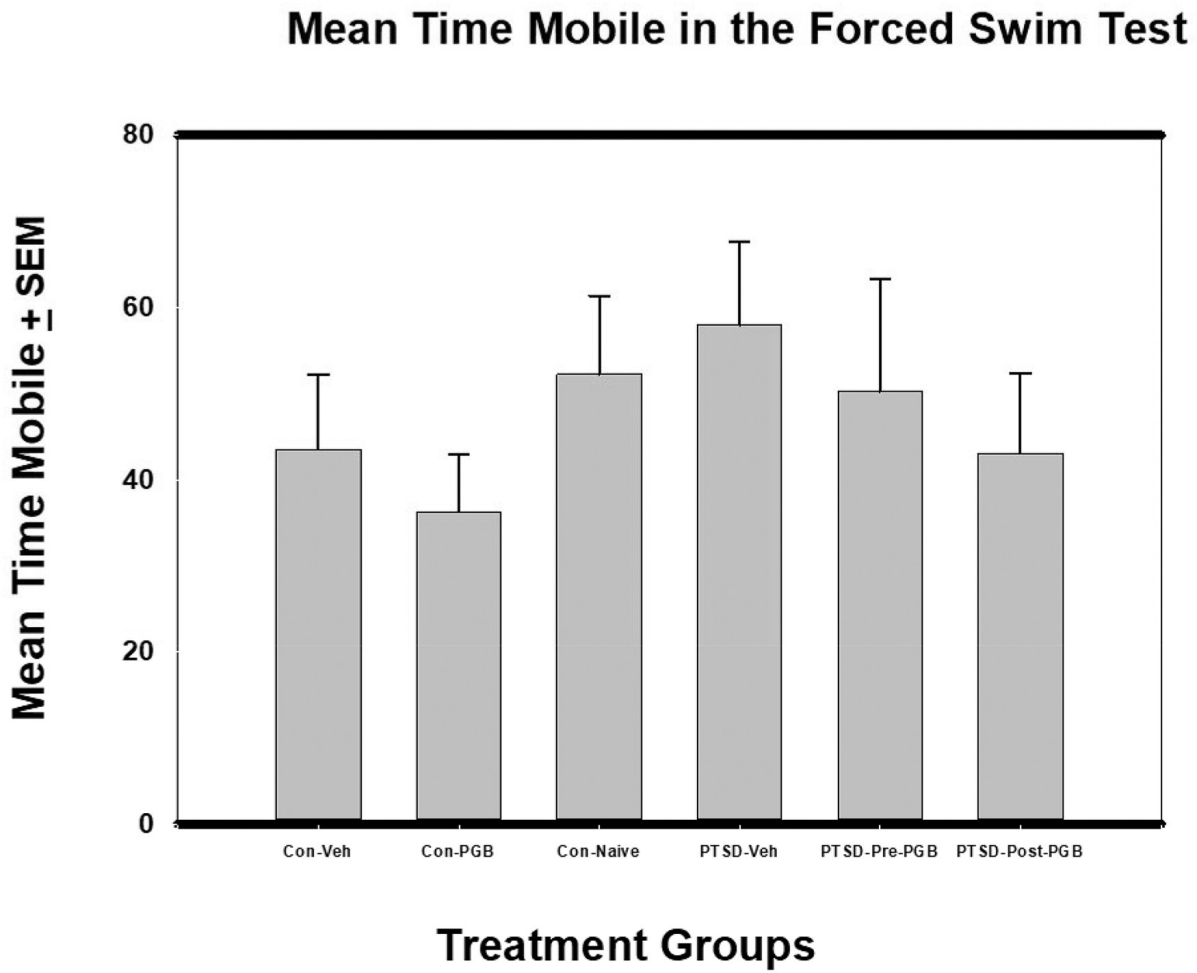
ANOVA of Mean Time Mobile from Forced Swim test (FST) and Standard Error of the Mean (SEM). Each of the main two groups (Stressed and Non-stressed) had three subgroups: non-stressed: control vehicle, control PGB, control naïve; and stressed: Posttraumatic stress disorder (PTSD) vehicle, PTSD Pre-PGB (prophylactic), PTSD Post-PGB (non-prophylactic). There was no significant difference between the six groups.

In the FST, for the Mean Time Mobile outcome, there was not a significant difference between the six groups: *F*(5, 53) = .635, *p* = .674 (η^2^ = .057). Though not significant, the PTSD vehicle group had the highest mean (*M* = 57.96) and the PGB control drug group the lowest (*M* = 36.27) (Fig 6).

### Fecal pellet output

Regarding fecal pellet output, there was not a significant difference between the six groups: *F*(5, 54) = .469, *p* = .798 (η^2^ = .042). Though not significant, the control naïve group had the highest mean (*M* = 5.7) and the PTSD Pre-PGB group the lowest (*M* = 4.3).

## Discussion

PTSD continues to increase in frequency and cause devastating consequences. The aim of this study was to investigate the prophylactic effects of pregabalin on neurobehavior in a rodent model of PTSD. The ability of pregabalin, administered before and after the induction of PTSD, to prevent the symptoms of anxiety, behavioral despair, and alterations in learning and spatial memory were evaluated.

The rodent model chosen for PTSD, using both restraint and shock, was used because of its use in modeling the human conditions of anxiety, stress, and depression [37, 38]. It has also been shown to induce neuro-structural changes in regions of the brain associated with these conditions [39]. The rodents in the PTSD group all lost weight, though not statistically significant, more weight when compared to the control groups. Nonetheless, this is a good indication that the rodents in the experimental groups were demonstrating symptoms of chronic stress and anxiety. Further supporting this model of PTSD is the food and water intake data, where two of the stressed animal groups demonstrated lower water intake. These data are encouraging as pregabalin administered prior to the stress event appears to have not only prevented decreased water consumption, but also the PTSD Pre-PGB group had the highest water consumption overall when compared to the three control groups. In addition, the PTSD Pre-PGB also had the highest food intake compared to the other groups. While this effect cannot be directly contributed to the pregabalin administration, it is a unique finding.

The elevated plus maze is based on the rodents’ natural behavior for free exploration of novel and unprotected environments using both light and dark choices. Rodents have a natural aversion to heights and open spaces; therefore, it is a good paradigm for anxiety and for the measurement of an increase or decrease in this neurobehavior. Considering pregabalin’s rapid absorption profile, its affinity for alpha-2-delta sub-units of voltage-gated calcium channels in the hippocampus and amygdala, and its ability to induce a conformational change in the calcium channel thereby reducing the release of excitatory neurotransmitters, it was hypothesized that pregabalin would provide protection and treatment from PTSD symptoms [40, 41]. All three stressed groups demonstrated an anticipated decrease in mean open arm time indicating an increase in anxiety-like behavior. While pretreatment with pregabalin did provide some modest protection in the reduction of exploratory behavior, the post-treatment pregabalin demonstrated no benefit when compared to the PTSD vehicle group. It is interesting to note that there was no significant difference between the amount of open arm exploratory time between the PTSD pretreatment group and the three non-stressed groups.

The Morris water maze was used to evaluate pre and post stress administration of pregabalin on spatial memory and learning. While the results from the pretreated pregabalin group were similar to the control naïve group, pregabalin’s ability to preserve spatial memory formation and learning was not demonstrated. In addition, pregabalin did not improve spatial memory formation and learning following the development of PTSD. In evaluating the Morris water maze data, it is possible that the study period was too short; as it has been shown that learning in the Morris water maze can be impaired for as late as three weeks after certain models of PTSD [37]. In addition, it has been reported with other studies that there can be a delay in the negative effects of stress on memory retrieval and learning [42–44].

While it has long been recognized that PTSD and depression are both clinically and biologically distinct in humans, there remains agreement that the two processes are inherently linked [45]. The forced swim test provided an opportunity to assess the learned helplessness and behavioral despair induced by the stress model and the effect of pregabalin in prevention and treatment. These data argue against a psychomotor effect of pregabalin as the rodents in the drug treatment groups had a decrease in mean time mobile.

In the rat model, an increase in stress is associated with an increase in fecal pellet output (FPO). Prior studies have found that pregabalin increased colonic compliance allowing for greater colonic volumes and a decrease in FPO [46]. While not a significant difference, the control-naïve group demonstrated the highest mean fecal pellet output, whereas the PTSD Pre-PGB group had the lowest mean. These results do not explain why the other pregabalin groups did not have a decrease in FPO. Further studies are required to fully understand this outcome.

Although no statistically significant findings were demonstrated in the neurobehavioral results there may be clinically significant correlations from this study. Even a modest decrease in the signs and symptoms of PTSD could translate into a clinically significant improvement in an individual’s quality of life.

After the implementation of the experiments and subsequent results, certain limitations of the current study were recognized. Enhanced biochemical assays and plasma biomarkers of stress, such as catecholamine levels and hypothalamus-pituitary-adrenal axis markers (plasma corticosterone and ACTH levels) could have been incorporated. These biomarkers may have added strength to the neurobehavior data and potentially validated different stress levels of the animals between groups. Furthermore, the fecal pellet output could have been analyzed for the corticosterone levels between the groups in order to provide indications of diverse stress levels. Although the sequential three neurobehavioral tests (EPM, MWM, and FST) was carefully considered and planned implementation from the least to most stressful test, another potential limitation is that the individual neurobehavioral test itself may have influenced the stress levels and behavioral results in each subsequent instrument.

Future research should include further biomarker assays to evaluate differences in stress levels and potentially conduct neurobehavioral testing in independent experiments, e.g., MWM and FST evaluation in separate sets of subjects to obviate any interaction effects from each test. Additionally, future studies should evaluate a longer pre-PTSD or prophylactic administration time period of PGB before the restraint-shock treatment. These studies may demonstrate more clinically significant benefits as it is known that some treatments of PTSD (i.e. antidepressants) may require an extended period of time to affect neurobehavior [47, 48] and to achieve higher steady state plasma levels of PGB. Furthermore, other studies which include longer post-PTSD time points (greater than 10 days) may also display more encouraging results.

## Conclusions

This study establishes a sound framework for future investigation of PTSD treatments. PTSD is a devastating, debilitating, and costly neuropathological outcome of trauma. It is critical that healthcare professionals investigate other potentially beneficial treatments to prevent and mitigate the neurobehavioral sequelae from PTSD. We recommend future studies evaluating a prophylactic regimen administered more than 24 hours prior to the stressful event. In addition, further consideration should be given to dosage and frequency of pregabalin administration after the post-traumatic event.

## Supporting information

S1 Fig ANCOVA for total body weight. Each of the main two groups (Stressed and Non-stressed) had three subgroups: non-stressed: control vehicle, control PGB, control naïve; and stressed: Posttraumatic stress disorder (PTSD) vehicle, PTSD Pre-PGB (prophylactic), PTSD Post-PGB (non-prophylactic). Mean total body weight is reported with the standard error of the mean (SEM).
(PDF)

S2 Fig ANCOVA for total water intake weight. Each of the main two groups (Stressed and Non-stressed) had three subgroups: non-stressed: control vehicle, control PGB, control naïve; and stressed: Posttraumatic stress disorder (PTSD) vehicle, PTSD Pre-PGB (prophylactic), PTSD Post-PGB (non-prophylactic). Mean total water intake weight is reported with the SEM. Asterisks denote statistical significance at *p* < .05. PTSD-Post-PGB was significantly less than PTSD-Pre-PGB and all three control groups. PTSD Veh was significantly lower than Con-Veh, Con-PGB, and PTSD-Pre-PGB. PTSD-Pre-PGB was significantly higher than Con-Naïve.
(PDF)

S3 Fig ANCOVA for total food intake weight. Each of the main two groups (Stressed and Non-stressed) had three subgroups: non-stressed: control vehicle, control PGB, control naïve, and stressed: Posttraumatic stress disorder (PTSD) vehicle, PTSD Pre-PGB (prophylactic), PTSD Post-PGB (non-prophylactic). Mean total food intake weight is reported with the SEM. Asterisks denote statistical significance at *p* < .05. PTSD-Pre-PGB was significantly higher than the PTSD-Post-PGB.
(PDF)

S4 Fig ANOVA of Mean Open-Arm Time Ratio (percent) from Elevated Plus-Maze (EPM) and Standard Error of the Mean (SEM). Each of the main two groups (Stressed and Non-stressed) had three subgroups: non-stressed: control vehicle, control PGB, control naïve, and stressed: Posttraumatic stress disorder (PTSD) vehicle, PTSD Pre-PGB (prophylactic), PTSD Post-PGB (non-prophylactic). Asterisks denote statistical significance at *p* < .05. PTSD vehicle and PTSD Post-PGB post-treatment had significantly lower mean open arm time ratio when compared to control. PTSD Post-PGB had significantly lower mean open arm time ratio when compared to Control PGB.
(PDF)

S5 Fig ANOVA of Time in Zone1 (Platform Zone) from Morris water maze (MWM) and Standard Error of the Mean (SEM). Each of the main two groups (Stressed and Non-stressed) had three subgroups: non-stressed: control vehicle, control PGB, control naïve; and stressed: Posttraumatic stress disorder (PTSD) vehicle, PTSD Pre-PGB (prophylactic), PTSD Post-PGB (non-prophylactic). Mean total body weight is reported with the standard error of the mean (SEM). There was not a significant difference between the six groups.
(PDF)

S6 Fig ANOVA of Mean Time Mobile from Forced Swim test (FST) and Standard Error of the Mean (SEM). Each of the main two groups (Stressed and Non-stressed) had three subgroups: non-stressed: control vehicle, control PGB, control naïve; and stressed: Posttraumatic stress disorder (PTSD) vehicle, PTSD Pre-PGB (prophylactic), PTSD Post-PGB (non-prophylactic). There was no significant difference between the six groups.
(PDF)
