# Supplementary material for: Effects of pregabalin on neurobehavior in an adult male rat model of PTSD
Source: PLoS One. 2018 Dec 31;13(12):e0209494. doi: 10.1371/journal.pone.0209494 (PMC6312257; doi:10.1371/journal.pone.0209494)
Supplement: S2 Fig — Each of the main two groups (Stressed and Non-stressed) had three subgroups: non-stressed: control vehicle, control PGB, control naïve; and stressed: Posttraumatic stress disorder (PTSD) vehicle, PTSD Pre-PGB (prophylactic), PTSD Post-PGB (non-prophylactic). Mean total water intake weight is reported with the SEM. Asterisks denote statistical significance at p < .05. PTSD-Post-PGB was significantly less than PTSD-Pre-PGB and all three control groups. PTSD Veh was significantly lower than Con-Veh, Con-PGB, and PTSD-Pre-PGB. PTSD-Pre-PGB was significantly higher than Con-Naïve. (PDF) [file pone.0209494.s002.pdf]

Weight Food Water

Study: Pregabalin & L-Theanine Prophylactic Effects on PTSD Behavior and Gene Expression in Male Sprague-Dawley Rats

Per the protocol the aims of this study are as follows (amended 10/13/17):

SPECIFIC AIMS

The aims of these studies are to determine if preemptive administration of PGB or L-Th prevent PTSD development in the rodent model. Specifically, the aims are as follows:

1. Determine the effects of PGB and L-Th on anxiety
2. Determine the effects of PGB and L-Th on locomotion
3. Determine the effects of PGB and L-Th on memory
4. Determine the effects of PGB and L-Th on depression
5. Determine the effects of PGB and L-Th on gene expression in the brain (p. 14).

And:

The aims of this research protocol will be guided by the following questions:

1. Is there a significant difference in the anxiolytic effects between the groups?
2. Is there a significant difference in locomotion between the groups?
3. Is there a significant difference in memory between the groups?
4. Is there a significant difference in depression between the groups?
5. Are there significant differences in gene expression and regulation in the hippocampus between the groups?
6. Are there significant differences in gene expression and regulation in the amygdala b etween the groups? (p. 15).

The grouping variable is as follows:

There was a total of 6 groups (1-6), each with 10 rat subjects.

The three groups of non-stressed rats:
1 - control-vehicle (received vehicle injections BID);
2 - L-Th control drug (received PGB BID)*
3 - control-naïve (received no injections)

The three groups in the 3-day restraint/shock stressed rats:
4 - PTSD-vehicle (received vehicle injection BID after three-day restraint/shock);
5 - PTSD-drug pre-treatment (received PGB BID 24 hours before and for a period of 10 days after three-day restraint shock);
6 - PTSD-post-treatment (received PGB injections BID for 10 days after three-day restraint/shock)

Group

		Frequency	Percent	Valid Percent	Cumulative Percent
Valid	1 control-vehicle	10	16.7	16.7	16.7
	2 L-Th control drug	10	16.7	16.7	33.3
	3 control-naïve	10	16.7	16.7	50.0
	4 PTSD-vehicle	10	16.7	16.7	66.7
	5 PTSD-drug pre-treatment	10	16.7	16.7	83.3
	6 PTSD-post-treatment	10	16.7	16.7	100.0
	Total	60	100.0	100.0	

Statistical Analysis: For this design a one-way ANOVA will be conducted for each of the outcome variables. All assumptions will be examined including homogeneity of error variances (via the Levine test) and normality. The eta-squared (η^2) effect size will be reported. Though interpreting and casting judgment as to what constitutes a small/medium/large effect size is context-dependent using Cohen's (1988) taxonomy .01/.059/138 will be small/medium/large. As well, all outliers and data anomalies will be examined and addressed accordingly (e.g., transformations, nonparametric options, etc.). In the event of a significant result ($\alpha = .05$) post hoc tests (e.g., Tukey's HSD) will be performed. Descriptive statistics and graphics will be provided for the full sample ($n = 60$) and by group.

Moreover, a separate analysis will be conducted using ANCOVA comparing the groups whilst controlling for days. As we can see below there are four cases with replacements, hence for the ANCOVA, sample size will be $n = 56$. The homogeneity of regression assumption was tested (i.e., testing the group x covariate interaction) and it was significant for the two following outcomes: TotalWaterIntake and TotalFoodWeightIntake.

Day Day

		Frequency	Percent	Valid Percent	Cumulative Percent
Valid	1	16	26.7	26.7	26.7
	2	17	28.3	28.3	55.0
	3	14	23.3	23.3	78.3
	4	9	15.0	15.0	93.3
	Replacements	4	6.7	6.7	100.0
	Total	60	100.0	100.0	

day_num Day

		Frequency	Percent	Valid Percent	Cumulative Percent
Valid	1.00	16	26.7	28.6	28.6
	2.00	17	28.3	30.4	58.9
	3.00	14	23.3	25.0	83.9
	4.00	9	15.0	16.1	100.0
	Total	56	93.3	100.0	
Missing	System	4	6.7		
Total		60	100.0		

Also, as noted below there are three instances of multiple rats per cage (one with 2 rats and 2 with 3 rats) so this may be a possible violation of independence, and should be duly noted.

Notes_Dup

		Frequency	Percent	Valid Percent	Cumulative Percent
Valid		52	86.7	86.7	86.7
	*2 Rats per cage	2	3.3	3.3	90.0
	3 rats in this cage	6	10.0	10.0	100.0
	Total	60	100.0	100.0	

Cohen (1988). *Statistical power analysis for the behavioral sciences*. (2nd Ed.). Hillsdale, NJ: Lawrence Erlbaum.

Descriptive Statistics: Full sample

Statistics

		WeightPreShockDate102517 Weight Pre-Shock Date 10-25-17	WeightondayofSacrifice Weight on day of Sacrifice	TotalWaterIntake Total Water Intake	TotalFoodWeightIntake Total Food Weight Intake
N	Valid	60	60	60	60
	Missing	0	0	0	0
Mean		282.03	316.90	791.37	519.60
Std. Error of Mean		2.052	2.836	15.507	9.946
Median		280.00	314.50	787.00	525.50
Mode		274	304[a]	796	494[a]
Std. Deviation		15.892	21.966	120.118	77.044
Variance		252.541	482.498	14428.338	5935.837
Skewness		1.049	.061	.801	.144
Std. Error of Skewness		.309	.309	.309	.309
Kurtosis		1.098	-.156	.561	.441
Std. Error of Kurtosis		.608	.608	.608	.608
Range		73	101	514	346
Minimum		254	261	589	353
Maximum		327	362	1103	699
Sum		16922	19014	47482	31176

a. Multiple modes exist. The smallest value is shown

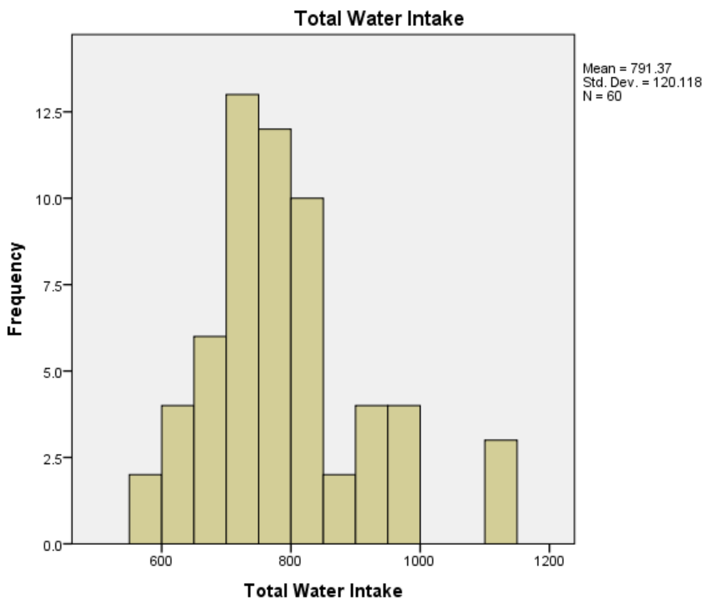

Descriptive Statistics: By Group

Statistics[a]

		WeightPreShockDate102517 Weight Pre-Shock Date 10-25-17	WeightondayofSacrifice Weight on day of Sacrifice	TotalWaterIntake Total Water Intake	TotalFoodWeightIntake Total Food Weight Intake
N	Valid	10	10	10	10
	Missing	0	0	0	0
Mean		283.60	331.00	828.00	530.00
Std. Error of Mean		5.271	5.890	20.497	12.540
Median		283.50	330.50	796.00	509.00
Mode		263[b]	310[b]	759[b]	503[b]
Std. Deviation		16.668	18.625	64.818	39.654
Variance		277.822	346.889	4201.333	1572.444
Skewness		.494	.372	.656	1.542
Std. Error of Skewness		.687	.687	.687	.687
Kurtosis		.145	-1.296	-1.159	.861
Std. Error of Kurtosis		1.334	1.334	1.334	1.334
Range		53	50	169	100
Minimum		263	310	759	503
Maximum		316	360	928	603
Sum		2836	3310	8280	5300

a. Group = 1 control-vehicle

b. Multiple modes exist. The smallest value is shown

Statistics[a]

		WeightPreShockDate102517 Weight Pre-Shock Date 10-25-17	Weightondayof Sacrifice Weight on day of Sacrifice	TotalWaterIntake Total Water Intake	TotalFoodWeightIntake Total Food Weight Intake
N	Valid	10	10	10	10
	Missing	0	0	0	0
Mean		281.90	322.50	850.60	515.60
Std. Error of Mean		4.393	6.647	32.142	10.672
Median		277.50	314.50	847.00	522.00
Mode		290	311	700[b]	467[b]
Std. Deviation		13.892	21.020	101.643	33.748
Variance		192.989	441.833	10331.378	1138.933
Skewness		1.290	.679	-.246	-.279
Std. Error of Skewness		.687	.687	.687	.687
Kurtosis		1.650	-.395	-.916	-1.230
Std. Error of Kurtosis		1.334	1.334	1.334	1.334
Range		44	67	282	91
Minimum		269	295	700	467
Maximum		313	362	982	558
Sum		2819	3225	8506	5156

a. Group = 2 L-Th control drug

b. Multiple modes exist. The smallest value is shown

Statistics[a]

		WeightPreShockDate102517 Weight Pre-Shock Date 10-25-17	Weightondayof Sacrifice Weight on day of Sacrifice	TotalWaterIntake Total Water Intake	TotalFoodWeightIntake Total Food Weight Intake
N	Valid	10	10	10	10
	Missing	0	0	0	0
Mean		269.00	320.20	829.80	543.80
Std. Error of Mean		2.966	3.574	26.804	9.098
Median		269.00	318.00	838.00	530.00
Mode		273	317	717[b]	526[b]
Std. Deviation		9.381	11.302	84.762	28.770
Variance		88.000	127.733	7184.622	827.733
Skewness		.156	.928	.365	1.717
Std. Error of Skewness		.687	.687	.687	.687
Kurtosis		-.544	1.238	-.358	1.279
Std. Error of Kurtosis		1.334	1.334	1.334	1.334
Range		31	39	245	72
Minimum		254	305	717	526
Maximum		285	344	962	598
Sum		2690	3202	8298	5438

a. Group = 3 control-naïve

b. Multiple modes exist. The smallest value is shown

Statistics[a]

		WeightPreShockDate102517 Weight Pre-Shock Date 10-25-17	WeightondayofSacrifice Weight on day of Sacrifice	TotalWaterIntake Total Water Intake	TotalFoodWeightIntake Total Food Weight Intake
N	Valid	10	10	10	10
	Missing	0	0	0	0
Mean		285.90	312.40	680.80	506.40
Std. Error of Mean		5.836	7.236	23.178	20.956
Median		281.00	306.00	690.00	474.00
Mode		295	286[b]	589[b]	441[b]
Std. Deviation		18.454	22.882	73.295	66.267
Variance		340.544	523.600	5372.178	4391.378
Skewness		1.212	1.073	.463	1.067
Std. Error of Skewness		.687	.687	.687	.687
Kurtosis		1.699	.700	-.528	-.096
Std. Error of Kurtosis		1.334	1.334	1.334	1.334
Range		61	74	207	179
Minimum		266	286	589	441
Maximum		327	360	796	620
Sum		2859	3124	6808	5064

a. Group = 4 PTSD-vehicle

b. Multiple modes exist. The smallest value is shown

Statistics[a]

		WeightPreShockDate102517 Weight Pre-Shock Date 10-25-17	WeightondayofSacrifice Weight on day of Sacrifice	TotalWaterIntake Total Water Intake	TotalFoodWeightIntake Total Food Weight Intake
N	Valid	10	10	10	10
	Missing	0	0	0	0
Mean		283.40	304.10	860.70	512.30
Std. Error of Mean		4.448	6.208	54.442	42.396
Median		281.50	301.00	790.00	461.50
Mode		267[b]	284	1103	699
Std. Deviation		14.065	19.632	172.161	134.069
Variance		197.822	385.433	29639.344	17974.456
Skewness		1.754	1.216	.828	.763
Std. Error of Skewness		.687	.687	.687	.687
Kurtosis		4.189	1.846	-1.343	-1.394
Std. Error of Kurtosis		1.334	1.334	1.334	1.334
Range		51	64	397	317
Minimum		267	284	706	382
Maximum		318	348	1103	699
Sum		2834	3041	8607	5123

a. Group = 5 PTSD-drug pre-treatment

b. Multiple modes exist. The smallest value is shown

Statistics[a]

		WeightPreShockDate102517 Weight Pre-Shock Date 10-25-17	WeightondayofSacrifice Weight on day of Sacrifice	TotalWaterIntake Total Water Intake	TotalFoodWeightIntake Total Food Weight Intake
N	Valid	10	10	10	10
	Missing	0	0	0	0
Mean		288.40	311.20	698.30	509.50
Std. Error of Mean		5.506	9.105	15.092	34.428
Median		282.50	314.50	729.50	534.50
Mode		277	336	733	623
Std. Deviation		17.411	28.794	47.726	108.871
Variance		303.156	829.067	2277.789	11852.944
Skewness		1.357	-.502	-.501	-.567
Std. Error of Skewness		.687	.687	.687	.687
Kurtosis		1.554	-.722	-2.087	-1.287
Std. Error of Kurtosis		1.334	1.334	1.334	1.334
Range		57	89	107	270
Minimum		270	261	635	353
Maximum		327	350	742	623
Sum		2884	3112	6983	5095

a. Group = 6 PTSD-post-treatment

Total Water Intake

Descriptive Statistics

Dependent Variable: TotalWaterIntake Total Water Intake

Group	Mean	Std. Deviation	N
1 control-vehicle	828.00	64.818	10
2 L-Th control drug	850.60	101.643	10
3 control-naïve	829.80	84.762	10
4 PTSD-vehicle	680.80	73.295	10
5 PTSD-drug pre-treatment	860.70	172.161	10
6 PTSD-post-treatment	698.30	47.726	10
Total	791.37	120.118	60

Levene's Test of Equality of Error Variances[a]

Dependent Variable: TotalWaterIntake Total Wa

F	df1	df2	Sig.
5.416	5	54	.000

Tests the null hypothesis that the error variance of the dependent variable is equal across groups.

a. Design: Intercept + Group

Tests of Between-Subjects Effects

Dependent Variable: TotalWaterIntake Total Water Intake

Source	Type III Sum of Squares	df	Mean Square	F	Sig.	Partial Eta Squared
Corrected Model	320212.133[a]	5	64042.427	6.512	.000	.376
Intercept	37575672.07	1	37575672.07	3820.824	.000	.986
Group	320212.133	5	64042.427	6.512	.000	.376
Error	531059.800	54	9834.441			
Total	38426944.00	60				
Corrected Total	851271.933	59				

a. R Squared = .376 (Adjusted R Squared = .318)

Multiple Comparisons

Dependent Variable: TotalWaterIntake Total Water Intake

Games-Howell

(I) Group	(J) Group	Mean Difference (I-J)	Std. Error	Sig.	95% Confidence Interval Lower Bound	Upper Bound
1 control-vehicle	2 L-Th control drug	-22.60	38.122	.990	-146.15	100.95
	3 control-naïve	-1.80	33.743	1.000	-109.85	106.25
	4 PTSD-vehicle	147.20*	30.941	.002	48.71	245.69
	5 PTSD-drug pre-treatment	-32.70	58.173	.992	-229.52	164.12
	6 PTSD-post-treatment	129.70*	25.454	.001	48.02	211.38
2 L-Th control drug	1 control-vehicle	22.60	38.122	.990	-100.95	146.15
	3 control-naïve	20.80	41.852	.996	-112.68	154.28
	4 PTSD-vehicle	169.80*	39.628	.006	42.47	297.13
	5 PTSD-drug pre-treatment	-10.10	63.222	1.000	-216.27	196.07
	6 PTSD-post-treatment	152.30*	35.509	.009	34.24	270.36
3 control-naïve	1 control-vehicle	1.80	33.743	1.000	-106.25	109.85
	2 L-Th control drug	-20.80	41.852	.996	-154.28	112.68
	4 PTSD-vehicle	149.00*	35.436	.006	36.13	261.87
	5 PTSD-drug pre-treatment	-30.90	60.683	.995	-231.85	170.05
	6 PTSD-post-treatment	131.50*	30.761	.008	30.79	232.21
4 PTSD-vehicle	1 control-vehicle	-147.20*	30.941	.002	-245.69	-48.71
	2 L-Th control drug	-169.80*	39.628	.006	-297.13	-42.47
	3 control-naïve	-149.00*	35.436	.006	-261.87	-36.13
	5 PTSD-drug pre-treatment	-179.90	59.171	.084	-378.22	18.42
	6 PTSD-post-treatment	-17.50	27.659	.987	-107.00	72.00
5 PTSD-drug pre-treatment	1 control-vehicle	32.70	58.173	.992	-164.12	229.52
	2 L-Th control drug	10.10	63.222	1.000	-196.07	216.27
	3 control-naïve	30.90	60.683	.995	-170.05	231.85
	4 PTSD-vehicle	179.90	59.171	.084	-18.42	378.22
	6 PTSD-post-treatment	162.40	56.495	.121	-32.40	357.20
6 PTSD-post-treatment	1 control-vehicle	-129.70*	25.454	.001	-211.38	-48.02
	2 L-Th control drug	-152.30*	35.509	.009	-270.36	-34.24
	3 control-naïve	-131.50*	30.761	.008	-232.21	-30.79
	4 PTSD-vehicle	17.50	27.659	.987	-72.00	107.00
	5 PTSD-drug pre-treatment	-162.40	56.495	.121	-357.20	32.40

Based on observed means.
The error term is Mean Square(Error) = 9834.441.

*. The mean difference is significant at the .05 level.

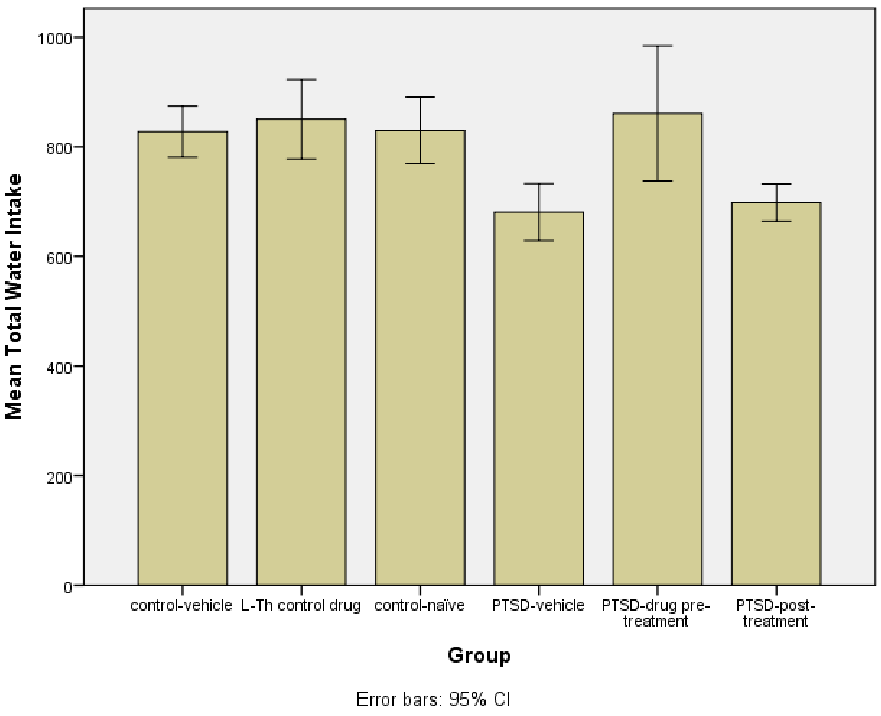

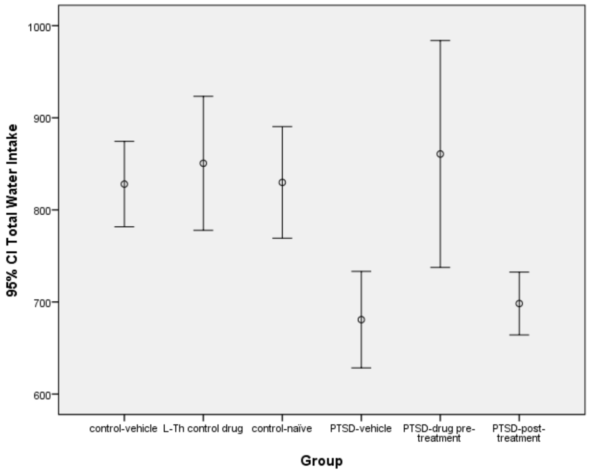

Summary: For the **Total Water Intake** outcome, there was a significant difference between the six groups: $F(5, 54) = 6.51$, $p < .001$ ($\eta^2 = .376$). The PTSD-drug pre-treatment group has the highest mean ($M = 860.7$) and PTSD-vehicle the lowest ($M = 680.8$). Given the violation of homogeneity of variance the Games-Howell (GH) post hoc test was used, which can be used in the event of heterogeneous variances. Per the GH test the PTSD post-treatment group was significantly lower than all three of the control groups. Similarly, the PTSD-vehicle was significantly lower than all three of the control groups.

Total Water Intake

Descriptive Statistics

Dependent Variable: TotalWaterIntake Total Water Intake

Group	Mean	Std. Deviation	N
1 control-vehicle	828.00	64.818	10
2 L-Th control drug	850.60	101.643	10
3 control-naïve	829.80	84.762	10
4 PTSD-vehicle	680.80	73.295	10
5 PTSD-drug pre-treatment	890.43	199.887	7
6 PTSD-post-treatment	695.22	49.557	9
Total	792.54	123.621	56

Levene's Test of Equality of Error Variances[a]

Dependent Variable: TotalWaterIntake Total Wa

F	df1	df2	Sig.
24.049	5	50	.000

Tests the null hypothesis that the error variance of the dependent variable is equal across groups.

a. Design: Intercept + day_num + Group

Tests of Between-Subjects Effects

Dependent Variable: TotalWaterIntake Total Water Intake

Source	Type III Sum of Squares	df	Mean Square	F	Sig.	Partial Eta Squared
Corrected Model	520046.630[a]	6	86674.438	13.252	.000	.619
Intercept	3237381.501	1	3237381.501	494.992	.000	.910
day_num	182709.571	1	182709.571	27.936	.000	.363
Group	440435.201	5	88087.040	13.468	.000	.579
Error	320473.299	49	6540.271			
Total	36014840.00	56				
Corrected Total	840519.929	55				

a. R Squared = .619 (Adjusted R Squared = .572)

Summary: For the **Total Water Intake** outcome when controlling for Days via one-way ANCOVA, there was a significant difference between the six groups: $F(5, 54) = 6.51$, $p < .001$ ($\eta^2 = .579$) and the covariate was significant ($p < .001$, $\eta^2 = .363$). The PTSD-drug pre treatment group has the highest mean ($M = 890.43$) and PTSD-vehicle the lowest ($M = 680.8$). The adjusted means are included in the table titled **"Estimates"**
