# Supplementary material for: Effects of pregabalin on neurobehavior in an adult male rat model of PTSD
Source: PLoS One. 2018 Dec 31;13(12):e0209494. doi: 10.1371/journal.pone.0209494 (PMC6312257; doi:10.1371/journal.pone.0209494)
Supplement: S4 Fig — Each of the main two groups (Stressed and Non-stressed) had three subgroups: non-stressed: control vehicle, control PGB, control naïve, and stressed: Posttraumatic stress disorder (PTSD) vehicle, PTSD Pre-PGB (prophylactic), PTSD Post-PGB (non-prophylactic). Asterisks denote statistical significance at p < .05. PTSD vehicle and PTSD Post-PGB post-treatment had significantly lower mean open arm time ratio when compared to control. PTSD Post-PGB had significantly lower mean open arm time ratio when compared to Control PGB. (PDF) [file pone.0209494.s004.pdf]

**Elevated Plus Maze (EPM)**

**Study: Pregabalin & L-Theanine Prophylactic Effects on PTSD Behavior and Gene Expression in Male Sprague-Dawley Rats**

Per the protocol the aims of this study are as follows (amended 10/13/17):

**SPECIFIC AIMS**

**The aims of these studies are to determine if preemptive administration of PGB or L-Th prevent PTSD development in the rodent model. Specifically, the aims are as follows:**

1. Determine the effects of PGB and L-Th on anxiety
2. Determine the effects of PGB and L-Th on locomotion
3. Determine the effects of PGB and L-Th on memory
4. Determine the effects of PGB and L-Th on depression
5. Determine the effects of PGB and L-Th on gene expression in the brain (p. 14).

And:

The aims of this research protocol will be guided by the following questions:

1. Is there a significant difference in the anxiolytic effects between the groups?
2. Is there a significant difference in locomotion between the groups?
3. Is there a significant difference in memory between the groups?
4. Is there a significant difference in depression between the groups?
5. Are there significant differences in gene expression and regulation in the hippocampus between the groups?
6. Are there significant differences in gene expression and regulation in the amygdala b etween the groups? (p. 15).

The grouping variable is as follows:

There was a total of 6 groups (1-6), each with 10 rat subjects.

**The three groups of non-stressed rats:**
1 - control-vehicle (received vehicle injections BID);
2 - L-Th control drug (received PGB BID)*
3 - control-naïve (received no injections)

**The three groups in the 3-day restraint/shock stressed rats:**
4 - PTSD-vehicle (received vehicle injection BID after three-day restraint/shock);
5 - PTSD-drug pre-treatment (received PGB BID 24 hours before and for a period of 10 days after three-day restraint shock );
6 - PTSD-post-treatment (received PGB injections BID for 10 days after three-day restraint/shock)

**Group**

| | | Frequency | Percent | Valid Percent | Cumulative Percent |
|---|---|---|---|---|---|
| Valid | 1 control-vehicle | 10 | 16.7 | 16.7 | 16.7 |
| | 2 L-Th control drug | 10 | 16.7 | 16.7 | 33.3 |
| | 3 control-naïve | 10 | 16.7 | 16.7 | 50.0 |
| | 4 PTSD-vehicle | 10 | 16.7 | 16.7 | 66.7 |
| | 5 PTSD-drug pre-treatment | 10 | 16.7 | 16.7 | 83.3 |
| | 6 PTSD-post-treatment | 10 | 16.7 | 16.7 | 100.0 |
| | Total | 60 | 100.0 | 100.0 | |

**Statistical Analysis:** For this design a one-way ANOVA will be conducted for each of the outcome variables. All assumptions will be examined including homogeneity of error variances (via the Levine test) and normality. The eta-squared ($\eta^2$) effect size will be reported. Though interpreting and casting judgment as to what constitutes a small/medium/large effect size is context-dependent using Cohen's (1988) taxonomy .01/.059/138 will be small/medium/large. As well, all outliers and data anomalies will be examined and addressed accordingly (e.g., transformations, nonparametric options, etc.). In the event of a significant result ($\alpha = .05$) post hoc tests (e.g., Tukey's HSD) will be performed. Descriptive statistics and graphics will be provided for the full sample ($n = 60$) and by group.

**Note: given one particular outlier, at the end of the document the Kruskal-Wallis (nonparametric) test will be ran, and, as well, one-way ANOVA without the outlier will be performed.**

Cohen (1988). *Statistical power analysis for the behavioral sciences*. (2nd Ed.). Hillsdale, NJ: Lawrence Erlbaum.

## Descriptive Statistics: Full sample

**Statistics**

| | | Distance | Meanspeed Mean speed | Timemobile Time mobile | Mobileepisodes Mobile episodes | Maxspeed Max speed | Timefreezing Time freezing | OpenArmsentries Open Arms : entries | OpenArmstime Open Arms : time | OpenArmstimeheadincentreout Open Arms : time head in / centre out | OpenArmTimeCenter Open Arm Time + Center | OpenArmTimeHeadInCenterOutCenterTimeCentertimeheadincentreout Open Arm Time+ Head In/Center Out + Center Time+Center : time head in / centre out | OpenArmsentriesCenterentries Open Arms : entries+Center : entries |
|---|---|---|---|---|---|---|---|---|---|---|---|---|---|
| N | Valid | 60 | 60 | 60 | 60 | 60 | 60 | 60 | 60 | 60 | 60 | 60 | 60 |
| | Missing | 0 | 0 | 0 | 0 | 0 | 0 | 0 | 0 | 0 | 0 | 0 | 0 |
| Mean | | 12.13950 | .04052 | 119.347 | 78.423 | .40412 | 22.798 | 7.12 | 25.170 | 32.257 | 76.3517 | 136.9083 | 31.25 |
| Std. Error of Mean | | .475778 | .001593 | 4.0314 | 6.3555 | .022036 | 3.1338 | .661 | 3.8903 | 2.3330 | 5.86559 | 7.67679 | 1.923 |
| Median | | 12.39350 | .04150 | 118.100 | 62.000 | .39450 | 12.850 | 6.00 | 17.900 | 30.350 | 65.1000 | 136.2500 | 31.00 |
| Mode | | 17.865 | .045 | 140.8 | 63.0 | .350 | 7.0 | 5 | 26.1[a] | 6.5[a] | 13.00[a] | 60.00 | 31[a] |
| Std. Deviation | | 3.685357 | .012338 | 31.2274 | 49.2299 | .170689 | 24.2744 | 5.119 | 30.1340 | 18.0716 | 45.43469 | 59.46416 | 14.898 |
| Variance | | 13.582 | .000 | 975.150 | 2423.579 | .029 | 589.249 | 26.206 | 908.057 | 326.581 | 2064.311 | 3535.986 | 221.953 |
| Skewness | | -.288 | -.282 | .418 | 2.022 | 5.670 | 2.334 | 1.073 | 3.816 | .441 | .854 | .185 | .017 |
| Std. Error of Skewness | | .309 | .309 | .309 | .309 | .309 | .309 | .309 | .309 | .309 | .309 | .309 | .309 |
| Kurtosis | | -.213 | -.226 | 2.456 | 2.740 | 39.121 | 6.781 | 1.423 | 20.061 | -.686 | .635 | -.813 | -.474 |
| Std. Error of Kurtosis | | .608 | .608 | .608 | .608 | .608 | .608 | .608 | .608 | .608 | .608 | .608 | .608 |
| Range | | 15.285 | .051 | 181.4 | 214.6 | 1.385 | 132.7 | 24 | 202.5 | 69.3 | 211.00 | 214.10 | 63 |
| Minimum | | 4.312 | .014 | 54.2 | 23.0 | .194 | .0 | 0 | .0 | 3.6 | 13.00 | 43.00 | 3 |
| Maximum | | 19.597 | .065 | 235.6 | 237.6 | 1.579 | 132.7 | 24 | 202.5 | 72.9 | 224.00 | 257.10 | 66 |
| Sum | | 728.370 | 2.431 | 7160.8 | 4705.4 | 24.247 | 1367.9 | 427 | 1510.2 | 1935.4 | 4581.10 | 8214.50 | 1875 |

a. Multiple modes exist. The smallest value is shown

**Distance**

| | | Frequency | Percent | Valid Percent | Cumulative Percent |
|---|---|---|---|---|---|
| Valid | 4.312 | 1 | 1.7 | 1.7 | 1.7 |
| | 4.385 | 1 | 1.7 | 1.7 | 3.3 |
| | 4.452 | 1 | 1.7 | 1.7 | 5.0 |
| | 4.900 | 1 | 1.7 | 1.7 | 6.7 |
| | 6.532 | 1 | 1.7 | 1.7 | 8.3 |
| | 6.685 | 1 | 1.7 | 1.7 | 10.0 |
| | 6.800 | 1 | 1.7 | 1.7 | 11.7 |
| | 7.174 | 1 | 1.7 | 1.7 | 13.3 |
| | 8.121 | 1 | 1.7 | 1.7 | 15.0 |
| | 8.142 | 1 | 1.7 | 1.7 | 16.7 |
| | 8.162 | 1 | 1.7 | 1.7 | 18.3 |
| | 8.880 | 1 | 1.7 | 1.7 | 20.0 |
| | 9.010 | 1 | 1.7 | 1.7 | 21.7 |
| | 9.754 | 1 | 1.7 | 1.7 | 23.3 |
| | 9.874 | 1 | 1.7 | 1.7 | 25.0 |
| | 9.907 | 1 | 1.7 | 1.7 | 26.7 |
| | 10.345 | 1 | 1.7 | 1.7 | 28.3 |
| | 10.454 | 1 | 1.7 | 1.7 | 30.0 |
| | 10.902 | 1 | 1.7 | 1.7 | 31.7 |
| | 11.127 | 1 | 1.7 | 1.7 | 33.3 |
| | 11.483 | 1 | 1.7 | 1.7 | 35.0 |
| | 11.498 | 1 | 1.7 | 1.7 | 36.7 |
| | 11.610 | 1 | 1.7 | 1.7 | 38.3 |
| | 11.735 | 1 | 1.7 | 1.7 | 40.0 |
| | 11.832 | 1 | 1.7 | 1.7 | 41.7 |
| | 11.934 | 1 | 1.7 | 1.7 | 43.3 |
| | 11.969 | 1 | 1.7 | 1.7 | 45.0 |
| | 12.082 | 1 | 1.7 | 1.7 | 46.7 |
| | 12.211 | 1 | 1.7 | 1.7 | 48.3 |
| | 12.318 | 1 | 1.7 | 1.7 | 50.0 |
| | 12.469 | 1 | 1.7 | 1.7 | 51.7 |
| | 12.828 | 1 | 1.7 | 1.7 | 53.3 |
| | 12.924 | 1 | 1.7 | 1.7 | 55.0 |
| | 13.087 | 1 | 1.7 | 1.7 | 56.7 |
| | 13.202 | 1 | 1.7 | 1.7 | 58.3 |
| | 13.294 | 1 | 1.7 | 1.7 | 60.0 |
| | 13.357 | 1 | 1.7 | 1.7 | 61.7 |
| | 13.401 | 1 | 1.7 | 1.7 | 63.3 |
| | 13.469 | 1 | 1.7 | 1.7 | 65.0 |
| | 13.487 | 1 | 1.7 | 1.7 | 66.7 |
| | 13.622 | 1 | 1.7 | 1.7 | 68.3 |
| | 13.821 | 1 | 1.7 | 1.7 | 70.0 |
| | 13.976 | 1 | 1.7 | 1.7 | 71.7 |
| | 14.389 | 1 | 1.7 | 1.7 | 73.3 |
| | 14.501 | 1 | 1.7 | 1.7 | 75.0 |
| | 14.603 | 1 | 1.7 | 1.7 | 76.7 |
| | 14.700 | 1 | 1.7 | 1.7 | 78.3 |
| | 14.891 | 1 | 1.7 | 1.7 | 80.0 |
| | 15.173 | 1 | 1.7 | 1.7 | 81.7 |
| | 15.191 | 1 | 1.7 | 1.7 | 83.3 |
| | 15.260 | 1 | 1.7 | 1.7 | 85.0 |
| | 15.303 | 1 | 1.7 | 1.7 | 86.7 |
| | 15.744 | 1 | 1.7 | 1.7 | 88.3 |
| | 16.704 | 1 | 1.7 | 1.7 | 90.0 |
| | 17.865 | 2 | 3.3 | 3.3 | 93.3 |
| | 18.107 | 1 | 1.7 | 1.7 | 95.0 |
| | 18.178 | 1 | 1.7 | 1.7 | 96.7 |
| | 18.772 | 1 | 1.7 | 1.7 | 98.3 |
| | 19.597 | 1 | 1.7 | 1.7 | 100.0 |
| | Total | 60 | 100.0 | 100.0 | |

**Meanspeed Mean speed**

| | | Frequency | Percent | Valid Percent | Cumulative Percent |
|---|---|---|---|---|---|
| Valid | .014 | 1 | 1.7 | 1.7 | 1.7 |
| | .015 | 2 | 3.3 | 3.3 | 5.0 |
| | .016 | 1 | 1.7 | 1.7 | 6.7 |
| | .022 | 2 | 3.3 | 3.3 | 10.0 |
| | .023 | 1 | 1.7 | 1.7 | 11.7 |
| | .024 | 1 | 1.7 | 1.7 | 13.3 |
| | .027 | 3 | 5.0 | 5.0 | 18.3 |
| | .030 | 2 | 3.3 | 3.3 | 21.7 |
| | .033 | 3 | 5.0 | 5.0 | 26.7 |
| | .034 | 1 | 1.7 | 1.7 | 28.3 |
| | .035 | 1 | 1.7 | 1.7 | 30.0 |
| | .036 | 1 | 1.7 | 1.7 | 31.7 |
| | .037 | 1 | 1.7 | 1.7 | 33.3 |
| | .038 | 2 | 3.3 | 3.3 | 36.7 |
| | .039 | 3 | 5.0 | 5.0 | 41.7 |
| | .040 | 3 | 5.0 | 5.0 | 46.7 |
| | .041 | 2 | 3.3 | 3.3 | 50.0 |
| | .042 | 1 | 1.7 | 1.7 | 51.7 |
| | .043 | 2 | 3.3 | 3.3 | 55.0 |
| | .044 | 3 | 5.0 | 5.0 | 60.0 |
| | .045 | 5 | 8.3 | 8.3 | 68.3 |
| | .046 | 1 | 1.7 | 1.7 | 70.0 |
| | .047 | 1 | 1.7 | 1.7 | 71.7 |
| | .048 | 2 | 3.3 | 3.3 | 75.0 |
| | .049 | 2 | 3.3 | 3.3 | 78.3 |
| | .050 | 1 | 1.7 | 1.7 | 80.0 |
| | .051 | 4 | 6.7 | 6.7 | 86.7 |
| | .052 | 1 | 1.7 | 1.7 | 88.3 |
| | .056 | 1 | 1.7 | 1.7 | 90.0 |
| | .060 | 3 | 5.0 | 5.0 | 95.0 |
| | .061 | 1 | 1.7 | 1.7 | 96.7 |
| | .063 | 1 | 1.7 | 1.7 | 98.3 |
| | .065 | 1 | 1.7 | 1.7 | 100.0 |
| | Total | 60 | 100.0 | 100.0 | |

**Timemobile Time mobile**

| | | Frequency | Percent | Valid Percent | Cumulative Percent |
|---|---|---|---|---|---|
| Valid | 54.2 | 1 | 1.7 | 1.7 | 1.7 |
| | 54.5 | 1 | 1.7 | 1.7 | 3.3 |
| | 55.7 | 1 | 1.7 | 1.7 | 5.0 |
| | 62.4 | 1 | 1.7 | 1.7 | 6.7 |
| | 69.6 | 1 | 1.7 | 1.7 | 8.3 |
| | 70.8 | 1 | 1.7 | 1.7 | 10.0 |
| | 82.9 | 1 | 1.7 | 1.7 | 11.7 |
| | 84.0 | 1 | 1.7 | 1.7 | 13.3 |
| | 87.7 | 1 | 1.7 | 1.7 | 15.0 |
| | 91.9 | 1 | 1.7 | 1.7 | 16.7 |
| | 92.8 | 1 | 1.7 | 1.7 | 18.3 |
| | 99.5 | 1 | 1.7 | 1.7 | 20.0 |
| | 102.1 | 1 | 1.7 | 1.7 | 21.7 |
| | 103.2 | 1 | 1.7 | 1.7 | 23.3 |
| | 104.0 | 1 | 1.7 | 1.7 | 25.0 |
| | 106.6 | 1 | 1.7 | 1.7 | 26.7 |
| | 107.6 | 1 | 1.7 | 1.7 | 28.3 |
| | 108.2 | 1 | 1.7 | 1.7 | 30.0 |
| | 108.9 | 1 | 1.7 | 1.7 | 31.7 |
| | 111.2 | 1 | 1.7 | 1.7 | 33.3 |
| | 113.4 | 1 | 1.7 | 1.7 | 35.0 |
| | 113.9 | 1 | 1.7 | 1.7 | 36.7 |
| | 115.3 | 1 | 1.7 | 1.7 | 38.3 |
| | 115.5 | 1 | 1.7 | 1.7 | 40.0 |
| | 115.8 | 1 | 1.7 | 1.7 | 41.7 |
| | 116.0 | 1 | 1.7 | 1.7 | 43.3 |
| | 116.1 | 1 | 1.7 | 1.7 | 45.0 |
| | 117.0 | 1 | 1.7 | 1.7 | 46.7 |
| | 117.4 | 1 | 1.7 | 1.7 | 48.3 |
| | 117.8 | 1 | 1.7 | 1.7 | 50.0 |
| | 118.4 | 1 | 1.7 | 1.7 | 51.7 |
| | 119.1 | 1 | 1.7 | 1.7 | 53.3 |
| | 120.1 | 1 | 1.7 | 1.7 | 55.0 |
| | 120.6 | 1 | 1.7 | 1.7 | 56.7 |
| | 121.4 | 1 | 1.7 | 1.7 | 58.3 |
| | 122.3 | 1 | 1.7 | 1.7 | 60.0 |
| | 124.2 | 1 | 1.7 | 1.7 | 61.7 |
| | 125.3 | 1 | 1.7 | 1.7 | 63.3 |
| | 127.1 | 1 | 1.7 | 1.7 | 65.0 |
| | 127.8 | 1 | 1.7 | 1.7 | 66.7 |
| | 131.1 | 1 | 1.7 | 1.7 | 68.3 |
| | 132.5 | 1 | 1.7 | 1.7 | 70.0 |
| | 134.0 | 1 | 1.7 | 1.7 | 71.7 |
| | 135.4 | 1 | 1.7 | 1.7 | 73.3 |
| | 136.2 | 1 | 1.7 | 1.7 | 75.0 |
| | 139.1 | 1 | 1.7 | 1.7 | 76.7 |
| | 139.3 | 1 | 1.7 | 1.7 | 78.3 |
| | 140.8 | 2 | 3.3 | 3.3 | 81.7 |
| | 143.7 | 1 | 1.7 | 1.7 | 83.3 |
| | 143.8 | 1 | 1.7 | 1.7 | 85.0 |
| | 152.8 | 1 | 1.7 | 1.7 | 86.7 |
| | 153.5 | 1 | 1.7 | 1.7 | 88.3 |
| | 154.2 | 1 | 1.7 | 1.7 | 90.0 |
| | 155.5 | 1 | 1.7 | 1.7 | 91.7 |
| | 159.6 | 1 | 1.7 | 1.7 | 93.3 |
| | 160.6 | 1 | 1.7 | 1.7 | 95.0 |
| | 163.4 | 1 | 1.7 | 1.7 | 96.7 |
| | 166.6 | 1 | 1.7 | 1.7 | 98.3 |
| | 235.6 | 1 | 1.7 | 1.7 | 100.0 |
| | Total | 60 | 100.0 | 100.0 | |

**Mobileepisodes Mobile episodes**

| | | Frequency | Percent | Valid Percent | Cumulative Percent |
|---|---|---|---|---|---|
| Valid | 23.0 | 1 | 1.7 | 1.7 | 1.7 |
| | 35.0 | 1 | 1.7 | 1.7 | 3.3 |
| | 46.0 | 1 | 1.7 | 1.7 | 5.0 |
| | 47.0 | 2 | 3.3 | 3.3 | 8.3 |
| | 51.0 | 3 | 5.0 | 5.0 | 13.3 |
| | 52.0 | 1 | 1.7 | 1.7 | 15.0 |
| | 54.0 | 2 | 3.3 | 3.3 | 18.3 |
| | 55.0 | 3 | 5.0 | 5.0 | 23.3 |
| | 56.0 | 2 | 3.3 | 3.3 | 26.7 |
| | 57.0 | 4 | 6.7 | 6.7 | 33.3 |
| | 58.0 | 5 | 8.3 | 8.3 | 41.7 |
| | 59.0 | 2 | 3.3 | 3.3 | 45.0 |
| | 60.0 | 1 | 1.7 | 1.7 | 46.7 |
| | 61.0 | 1 | 1.7 | 1.7 | 48.3 |
| | 62.0 | 4 | 6.7 | 6.7 | 55.0 |
| | 63.0 | 6 | 10.0 | 10.0 | 65.0 |
| | 64.0 | 2 | 3.3 | 3.3 | 68.3 |
| | 65.0 | 3 | 5.0 | 5.0 | 73.3 |
| | 66.0 | 1 | 1.7 | 1.7 | 75.0 |
| | 67.0 | 2 | 3.3 | 3.3 | 78.3 |
| | 68.0 | 1 | 1.7 | 1.7 | 80.0 |
| | 69.0 | 1 | 1.7 | 1.7 | 81.7 |
| | 76.0 | 1 | 1.7 | 1.7 | 83.3 |
| | 78.0 | 1 | 1.7 | 1.7 | 85.0 |
| | 159.2 | 1 | 1.7 | 1.7 | 86.7 |
| | 168.9 | 1 | 1.7 | 1.7 | 88.3 |
| | 175.8 | 1 | 1.7 | 1.7 | 90.0 |
| | 179.9 | 1 | 1.7 | 1.7 | 91.7 |
| | 193.4 | 1 | 1.7 | 1.7 | 93.3 |
| | 197.9 | 1 | 1.7 | 1.7 | 95.0 |
| | 200.5 | 1 | 1.7 | 1.7 | 96.7 |
| | 207.2 | 1 | 1.7 | 1.7 | 98.3 |
| | 237.6 | 1 | 1.7 | 1.7 | 100.0 |
| | Total | 60 | 100.0 | 100.0 | |

**Maxspeed Max speed**

| | | Frequency | Percent | Valid Percent | Cumulative Percent |
|---|---|---|---|---|---|
| Valid | .194 | 1 | 1.7 | 1.7 | 1.7 |
| | .251 | 1 | 1.7 | 1.7 | 3.3 |
| | .270 | 1 | 1.7 | 1.7 | 5.0 |
| | .279 | 1 | 1.7 | 1.7 | 6.7 |
| | .280 | 1 | 1.7 | 1.7 | 8.3 |
| | .285 | 1 | 1.7 | 1.7 | 10.0 |
| | .287 | 1 | 1.7 | 1.7 | 11.7 |
| | .300 | 1 | 1.7 | 1.7 | 13.3 |
| | .306 | 1 | 1.7 | 1.7 | 15.0 |
| | .316 | 1 | 1.7 | 1.7 | 16.7 |
| | .320 | 1 | 1.7 | 1.7 | 18.3 |
| | .325 | 2 | 3.3 | 3.3 | 21.7 |
| | .330 | 1 | 1.7 | 1.7 | 23.3 |
| | .332 | 1 | 1.7 | 1.7 | 25.0 |
| | .340 | 2 | 3.3 | 3.3 | 28.3 |
| | .348 | 1 | 1.7 | 1.7 | 30.0 |
| | .350 | 4 | 6.7 | 6.7 | 36.7 |
| | .355 | 1 | 1.7 | 1.7 | 38.3 |
| | .369 | 1 | 1.7 | 1.7 | 40.0 |
| | .375 | 3 | 5.0 | 5.0 | 45.0 |
| | .379 | 1 | 1.7 | 1.7 | 46.7 |
| | .389 | 1 | 1.7 | 1.7 | 48.3 |
| | .394 | 1 | 1.7 | 1.7 | 50.0 |
| | .395 | 1 | 1.7 | 1.7 | 51.7 |
| | .397 | 1 | 1.7 | 1.7 | 53.3 |
| | .399 | 1 | 1.7 | 1.7 | 55.0 |
| | .401 | 2 | 3.3 | 3.3 | 58.3 |
| | .404 | 1 | 1.7 | 1.7 | 60.0 |
| | .407 | 1 | 1.7 | 1.7 | 61.7 |
| | .408 | 1 | 1.7 | 1.7 | 63.3 |
| | .409 | 2 | 3.3 | 3.3 | 66.7 |
| | .411 | 1 | 1.7 | 1.7 | 68.3 |
| | .424 | 1 | 1.7 | 1.7 | 70.0 |
| | .428 | 1 | 1.7 | 1.7 | 71.7 |
| | .434 | 1 | 1.7 | 1.7 | 73.3 |
| | .439 | 1 | 1.7 | 1.7 | 75.0 |
| | .442 | 1 | 1.7 | 1.7 | 76.7 |
| | .444 | 1 | 1.7 | 1.7 | 78.3 |
| | .449 | 2 | 3.3 | 3.3 | 81.7 |
| | .464 | 2 | 3.3 | 3.3 | 85.0 |
| | .465 | 1 | 1.7 | 1.7 | 86.7 |
| | .468 | 2 | 3.3 | 3.3 | 90.0 |
| | .469 | 1 | 1.7 | 1.7 | 91.7 |
| | .472 | 1 | 1.7 | 1.7 | 93.3 |
| | .476 | 1 | 1.7 | 1.7 | 95.0 |
| | .499 | 1 | 1.7 | 1.7 | 96.7 |
| | .634 | 1 | 1.7 | 1.7 | 98.3 |
| | 1.579 | 1 | 1.7 | 1.7 | 100.0 |
| | Total | 60 | 100.0 | 100.0 | |

**Timefreezing Time freezing**

| | | Frequency | Percent | Valid Percent | Cumulative Percent |
|---|---|---|---|---|---|
| Valid | .0 | 1 | 1.7 | 1.7 | 1.7 |
| | 4.0 | 2 | 3.3 | 3.3 | 5.0 |
| | 5.0 | 2 | 3.3 | 3.3 | 8.3 |
| | 6.0 | 4 | 6.7 | 6.7 | 15.0 |
| | 6.6 | 1 | 1.7 | 1.7 | 16.7 |
| | 7.0 | 5 | 8.3 | 8.3 | 25.0 |
| | 8.0 | 2 | 3.3 | 3.3 | 28.3 |
| | 9.0 | 3 | 5.0 | 5.0 | 33.3 |
| | 9.4 | 1 | 1.7 | 1.7 | 35.0 |
| | 10.0 | 3 | 5.0 | 5.0 | 40.0 |
| | 11.0 | 1 | 1.7 | 1.7 | 41.7 |
| | 11.2 | 1 | 1.7 | 1.7 | 43.3 |
| | 11.5 | 1 | 1.7 | 1.7 | 45.0 |
| | 12.0 | 2 | 3.3 | 3.3 | 48.3 |
| | 12.7 | 1 | 1.7 | 1.7 | 50.0 |
| | 13.0 | 3 | 5.0 | 5.0 | 55.0 |
| | 13.4 | 1 | 1.7 | 1.7 | 56.7 |
| | 13.6 | 1 | 1.7 | 1.7 | 58.3 |
| | 14.0 | 1 | 1.7 | 1.7 | 60.0 |
| | 14.7 | 1 | 1.7 | 1.7 | 61.7 |
| | 17.6 | 1 | 1.7 | 1.7 | 63.3 |
| | 19.4 | 1 | 1.7 | 1.7 | 65.0 |
| | 23.0 | 1 | 1.7 | 1.7 | 66.7 |
| | 23.4 | 1 | 1.7 | 1.7 | 68.3 |
| | 24.0 | 1 | 1.7 | 1.7 | 70.0 |
| | 26.0 | 1 | 1.7 | 1.7 | 71.7 |
| | 26.1 | 1 | 1.7 | 1.7 | 73.3 |
| | 27.1 | 1 | 1.7 | 1.7 | 75.0 |
| | 28.5 | 1 | 1.7 | 1.7 | 76.7 |
| | 31.1 | 1 | 1.7 | 1.7 | 78.3 |
| | 36.1 | 1 | 1.7 | 1.7 | 80.0 |
| | 41.6 | 1 | 1.7 | 1.7 | 81.7 |
| | 45.0 | 1 | 1.7 | 1.7 | 83.3 |
| | 45.3 | 1 | 1.7 | 1.7 | 85.0 |
| | 46.4 | 1 | 1.7 | 1.7 | 86.7 |
| | 46.6 | 1 | 1.7 | 1.7 | 88.3 |
| | 51.2 | 1 | 1.7 | 1.7 | 90.0 |
| | 56.4 | 1 | 1.7 | 1.7 | 91.7 |
| | 57.5 | 1 | 1.7 | 1.7 | 93.3 |
| | 64.4 | 1 | 1.7 | 1.7 | 95.0 |
| | 79.0 | 1 | 1.7 | 1.7 | 96.7 |
| | 88.4 | 1 | 1.7 | 1.7 | 98.3 |
| | 132.7 | 1 | 1.7 | 1.7 | 100.0 |
| | Total | 60 | 100.0 | 100.0 | |

**OpenArmsentries Open Arms : entries**

| | | Frequency | Percent | Valid Percent | Cumulative Percent |
|---|---|---|---|---|---|
| Valid | 0 | 1 | 1.7 | 1.7 | 1.7 |
| | 1 | 5 | 8.3 | 8.3 | 10.0 |
| | 2 | 8 | 13.3 | 13.3 | 23.3 |
| | 3 | 2 | 3.3 | 3.3 | 26.7 |
| | 4 | 3 | 5.0 | 5.0 | 31.7 |
| | 5 | 10 | 16.7 | 16.7 | 48.3 |
| | 6 | 4 | 6.7 | 6.7 | 55.0 |
| | 7 | 3 | 5.0 | 5.0 | 60.0 |
| | 8 | 2 | 3.3 | 3.3 | 63.3 |
| | 9 | 4 | 6.7 | 6.7 | 70.0 |
| | 10 | 3 | 5.0 | 5.0 | 75.0 |
| | 11 | 3 | 5.0 | 5.0 | 80.0 |
| | 12 | 5 | 8.3 | 8.3 | 88.3 |
| | 13 | 1 | 1.7 | 1.7 | 90.0 |
| | 14 | 3 | 5.0 | 5.0 | 95.0 |
| | 17 | 1 | 1.7 | 1.7 | 96.7 |
| | 22 | 1 | 1.7 | 1.7 | 98.3 |
| | 24 | 1 | 1.7 | 1.7 | 100.0 |
| | Total | 60 | 100.0 | 100.0 | |

**OpenArmstime Open Arms : time**

| | | Frequency | Percent | Valid Percent | Cumulative Percent |
|---|---|---|---|---|---|
| Valid | .0 | 1 | 1.7 | 1.7 | 1.7 |
| | .9 | 1 | 1.7 | 1.7 | 3.3 |
| | 1.8 | 1 | 1.7 | 1.7 | 5.0 |
| | 1.9 | 1 | 1.7 | 1.7 | 6.7 |
| | 2.1 | 1 | 1.7 | 1.7 | 8.3 |
| | 2.3 | 1 | 1.7 | 1.7 | 10.0 |
| | 3.6 | 1 | 1.7 | 1.7 | 11.7 |
| | 4.6 | 1 | 1.7 | 1.7 | 13.3 |
| | 5.2 | 1 | 1.7 | 1.7 | 15.0 |
| | 5.9 | 1 | 1.7 | 1.7 | 16.7 |
| | 6.5 | 1 | 1.7 | 1.7 | 18.3 |
| | 6.9 | 1 | 1.7 | 1.7 | 20.0 |
| | 7.2 | 1 | 1.7 | 1.7 | 21.7 |
| | 7.3 | 1 | 1.7 | 1.7 | 23.3 |
| | 7.5 | 1 | 1.7 | 1.7 | 25.0 |
| | 8.9 | 1 | 1.7 | 1.7 | 26.7 |
| | 9.3 | 1 | 1.7 | 1.7 | 28.3 |
| | 9.7 | 1 | 1.7 | 1.7 | 30.0 |
| | 9.9 | 1 | 1.7 | 1.7 | 31.7 |
| | 10.0 | 1 | 1.7 | 1.7 | 33.3 |
| | 10.5 | 1 | 1.7 | 1.7 | 35.0 |
| | 12.8 | 1 | 1.7 | 1.7 | 36.7 |
| | 13.0 | 1 | 1.7 | 1.7 | 38.3 |
| | 13.1 | 1 | 1.7 | 1.7 | 40.0 |
| | 13.7 | 1 | 1.7 | 1.7 | 41.7 |
| | 14.4 | 1 | 1.7 | 1.7 | 43.3 |
| | 14.5 | 1 | 1.7 | 1.7 | 45.0 |
| | 14.6 | 1 | 1.7 | 1.7 | 46.7 |
| | 15.1 | 1 | 1.7 | 1.7 | 48.3 |
| | 17.7 | 1 | 1.7 | 1.7 | 50.0 |
| | 18.1 | 1 | 1.7 | 1.7 | 51.7 |
| | 18.3 | 1 | 1.7 | 1.7 | 53.3 |
| | 18.6 | 1 | 1.7 | 1.7 | 55.0 |
| | 18.8 | 1 | 1.7 | 1.7 | 56.7 |
| | 20.3 | 1 | 1.7 | 1.7 | 58.3 |
| | 20.6 | 1 | 1.7 | 1.7 | 60.0 |
| | 21.3 | 1 | 1.7 | 1.7 | 61.7 |
| | 22.1 | 1 | 1.7 | 1.7 | 63.3 |
| | 24.5 | 1 | 1.7 | 1.7 | 65.0 |
| | 25.6 | 1 | 1.7 | 1.7 | 66.7 |
| | 26.1 | 2 | 3.3 | 3.3 | 70.0 |
| | 26.4 | 1 | 1.7 | 1.7 | 71.7 |
| | 26.7 | 1 | 1.7 | 1.7 | 73.3 |
| | 30.9 | 1 | 1.7 | 1.7 | 75.0 |
| | 31.1 | 1 | 1.7 | 1.7 | 76.7 |
| | 34.7 | 1 | 1.7 | 1.7 | 78.3 |
| | 39.4 | 1 | 1.7 | 1.7 | 80.0 |
| | 41.8 | 1 | 1.7 | 1.7 | 81.7 |
| | 41.9 | 1 | 1.7 | 1.7 | 83.3 |
| | 43.1 | 1 | 1.7 | 1.7 | 85.0 |
| | 47.5 | 1 | 1.7 | 1.7 | 86.7 |
| | 48.0 | 2 | 3.3 | 3.3 | 90.0 |
| | 48.4 | 1 | 1.7 | 1.7 | 91.7 |
| | 54.7 | 1 | 1.7 | 1.7 | 93.3 |
| | 68.1 | 1 | 1.7 | 1.7 | 95.0 |
| | 80.4 | 1 | 1.7 | 1.7 | 96.7 |
| | 85.3 | 1 | 1.7 | 1.7 | 98.3 |
| | 202.5 | 1 | 1.7 | 1.7 | 100.0 |
| | Total | 60 | 100.0 | 100.0 | |

**OpenArmstimeheadincentreout Open Arms : time head in / centre out**

| | | Frequency | Percent | Valid Percent | Cumulative Percent |
|---|---|---|---|---|---|
| Valid | 3.6 | 1 | 1.7 | 1.7 | 1.7 |
| | 4.4 | 1 | 1.7 | 1.7 | 3.3 |
| | 6.1 | 1 | 1.7 | 1.7 | 5.0 |
| | 6.5 | 2 | 3.3 | 3.3 | 8.3 |
| | 10.4 | 1 | 1.7 | 1.7 | 10.0 |
| | 11.1 | 1 | 1.7 | 1.7 | 11.7 |
| | 13.2 | 1 | 1.7 | 1.7 | 13.3 |
| | 13.4 | 1 | 1.7 | 1.7 | 15.0 |
| | 14.5 | 1 | 1.7 | 1.7 | 16.7 |
| | 15.1 | 1 | 1.7 | 1.7 | 18.3 |
| | 16.6 | 1 | 1.7 | 1.7 | 20.0 |
| | 17.4 | 1 | 1.7 | 1.7 | 21.7 |
| | 18.1 | 1 | 1.7 | 1.7 | 23.3 |
| | 18.6 | 1 | 1.7 | 1.7 | 25.0 |
| | 18.7 | 1 | 1.7 | 1.7 | 26.7 |
| | 19.0 | 1 | 1.7 | 1.7 | 28.3 |
| | 19.1 | 2 | 3.3 | 3.3 | 31.7 |
| | 19.3 | 1 | 1.7 | 1.7 | 33.3 |
| | 20.7 | 1 | 1.7 | 1.7 | 35.0 |
| | 21.3 | 1 | 1.7 | 1.7 | 36.7 |
| | 22.1 | 1 | 1.7 | 1.7 | 38.3 |
| | 22.7 | 1 | 1.7 | 1.7 | 40.0 |
| | 23.4 | 1 | 1.7 | 1.7 | 41.7 |
| | 26.3 | 1 | 1.7 | 1.7 | 43.3 |
| | 26.7 | 1 | 1.7 | 1.7 | 45.0 |
| | 27.1 | 1 | 1.7 | 1.7 | 46.7 |
| | 29.9 | 1 | 1.7 | 1.7 | 48.3 |
| | 30.1 | 1 | 1.7 | 1.7 | 50.0 |
| | 30.6 | 1 | 1.7 | 1.7 | 51.7 |
| | 31.9 | 1 | 1.7 | 1.7 | 53.3 |
| | 33.0 | 1 | 1.7 | 1.7 | 55.0 |
| | 33.5 | 1 | 1.7 | 1.7 | 56.7 |
| | 33.8 | 1 | 1.7 | 1.7 | 58.3 |
| | 35.1 | 1 | 1.7 | 1.7 | 60.0 |
| | 36.0 | 1 | 1.7 | 1.7 | 61.7 |
| | 36.1 | 1 | 1.7 | 1.7 | 63.3 |
| | 37.1 | 2 | 3.3 | 3.3 | 66.7 |
| | 37.4 | 1 | 1.7 | 1.7 | 68.3 |
| | 39.1 | 1 | 1.7 | 1.7 | 70.0 |
| | 41.4 | 1 | 1.7 | 1.7 | 71.7 |
| | 41.7 | 1 | 1.7 | 1.7 | 73.3 |
| | 45.2 | 1 | 1.7 | 1.7 | 75.0 |
| | 46.0 | 1 | 1.7 | 1.7 | 76.7 |
| | 50.4 | 1 | 1.7 | 1.7 | 78.3 |
| | 51.0 | 1 | 1.7 | 1.7 | 80.0 |
| | 52.6 | 1 | 1.7 | 1.7 | 81.7 |
| | 53.4 | 1 | 1.7 | 1.7 | 83.3 |
| | 54.1 | 1 | 1.7 | 1.7 | 85.0 |
| | 54.3 | 1 | 1.7 | 1.7 | 86.7 |
| | 55.4 | 1 | 1.7 | 1.7 | 88.3 |
| | 57.5 | 1 | 1.7 | 1.7 | 90.0 |
| | 57.8 | 1 | 1.7 | 1.7 | 91.7 |
| | 60.4 | 2 | 3.3 | 3.3 | 95.0 |
| | 68.6 | 1 | 1.7 | 1.7 | 96.7 |
| | 70.6 | 1 | 1.7 | 1.7 | 98.3 |
| | 72.9 | 1 | 1.7 | 1.7 | 100.0 |
| | Total | 60 | 100.0 | 100.0 | |

**OpenArmTimeCenter Open Arm Time + Center**

| | | Frequency | Percent | Valid Percent | Cumulative Percent |
|---|---|---|---|---|---|
| Valid | 13.00 | 1 | 1.7 | 1.7 | 1.7 |
| | 14.60 | 1 | 1.7 | 1.7 | 3.3 |
| | 15.00 | 1 | 1.7 | 1.7 | 5.0 |
| | 19.50 | 1 | 1.7 | 1.7 | 6.7 |
| | 20.20 | 1 | 1.7 | 1.7 | 8.3 |
| | 20.60 | 1 | 1.7 | 1.7 | 10.0 |
| | 22.70 | 1 | 1.7 | 1.7 | 11.7 |
| | 22.90 | 1 | 1.7 | 1.7 | 13.3 |
| | 27.20 | 1 | 1.7 | 1.7 | 15.0 |
| | 29.80 | 1 | 1.7 | 1.7 | 16.7 |
| | 30.60 | 1 | 1.7 | 1.7 | 18.3 |
| | 35.80 | 1 | 1.7 | 1.7 | 20.0 |
| | 36.60 | 1 | 1.7 | 1.7 | 21.7 |
| | 38.50 | 1 | 1.7 | 1.7 | 23.3 |
| | 41.40 | 1 | 1.7 | 1.7 | 25.0 |
| | 43.70 | 1 | 1.7 | 1.7 | 26.7 |
| | 44.00 | 1 | 1.7 | 1.7 | 28.3 |
| | 47.60 | 1 | 1.7 | 1.7 | 30.0 |
| | 51.10 | 1 | 1.7 | 1.7 | 31.7 |
| | 55.10 | 1 | 1.7 | 1.7 | 33.3 |
| | 55.80 | 1 | 1.7 | 1.7 | 35.0 |
| | 58.70 | 1 | 1.7 | 1.7 | 36.7 |
| | 59.80 | 1 | 1.7 | 1.7 | 38.3 |
| | 59.80 | 1 | 1.7 | 1.7 | 40.0 |
| | 61.80 | 1 | 1.7 | 1.7 | 41.7 |
| | 62.00 | 1 | 1.7 | 1.7 | 43.3 |
| | 62.60 | 1 | 1.7 | 1.7 | 45.0 |
| | 63.00 | 1 | 1.7 | 1.7 | 46.7 |
| | 63.30 | 1 | 1.7 | 1.7 | 48.3 |
| | 64.10 | 1 | 1.7 | 1.7 | 50.0 |
| | 66.10 | 1 | 1.7 | 1.7 | 51.7 |
| | 66.90 | 1 | 1.7 | 1.7 | 53.3 |
| | 67.60 | 1 | 1.7 | 1.7 | 55.0 |
| | 69.10 | 1 | 1.7 | 1.7 | 56.7 |
| | 73.30 | 1 | 1.7 | 1.7 | 58.3 |
| | 73.80 | 1 | 1.7 | 1.7 | 60.0 |
| | 79.20 | 1 | 1.7 | 1.7 | 61.7 |
| | 79.80 | 1 | 1.7 | 1.7 | 63.3 |
| | 81.70 | 1 | 1.7 | 1.7 | 65.0 |
| | 88.10 | 1 | 1.7 | 1.7 | 66.7 |
| | 97.20 | 1 | 1.7 | 1.7 | 68.3 |
| | 97.50 | 1 | 1.7 | 1.7 | 70.0 |
| | 101.10 | 1 | 1.7 | 1.7 | 71.7 |
| | 104.50 | 1 | 1.7 | 1.7 | 73.3 |
| | 109.80 | 1 | 1.7 | 1.7 | 75.0 |
| | 110.10 | 1 | 1.7 | 1.7 | 76.7 |
| | 110.30 | 1 | 1.7 | 1.7 | 78.3 |
| | 111.40 | 1 | 1.7 | 1.7 | 80.0 |
| | 115.40 | 1 | 1.7 | 1.7 | 81.7 |
| | 120.00 | 1 | 1.7 | 1.7 | 83.3 |
| | 123.00 | 1 | 1.7 | 1.7 | 85.0 |
| | 125.30 | 1 | 1.7 | 1.7 | 86.7 |
| | 127.20 | 1 | 1.7 | 1.7 | 88.3 |
| | 128.80 | 1 | 1.7 | 1.7 | 90.0 |
| | 149.90 | 1 | 1.7 | 1.7 | 91.7 |
| | 153.80 | 1 | 1.7 | 1.7 | 93.3 |
| | 156.10 | 1 | 1.7 | 1.7 | 95.0 |
| | 159.50 | 1 | 1.7 | 1.7 | 96.7 |
| | 169.80 | 1 | 1.7 | 1.7 | 98.3 |
| | 224.00 | 1 | 1.7 | 1.7 | 100.0 |
| | Total | 60 | 100.0 | 100.0 | |

**OpenArmTimeHeadInCenterOutCenterTimeCentertimeheadincentreout Open Arm Time+ Head In/Center Out + Center Time+Center : time head in / centre out**

| | | Frequency | Percent | Valid Percent | Cumulative Percent |
|---|---|---|---|---|---|
| Valid | 43.00 | 1 | 1.7 | 1.7 | 1.7 |
| | 43.40 | 1 | 1.7 | 1.7 | 3.3 |
| | 50.80 | 1 | 1.7 | 1.7 | 5.0 |
| | 51.30 | 1 | 1.7 | 1.7 | 6.7 |
| | 51.70 | 1 | 1.7 | 1.7 | 8.3 |
| | 52.30 | 1 | 1.7 | 1.7 | 10.0 |
| | 52.70 | 1 | 1.7 | 1.7 | 11.7 |
| | 60.00 | 2 | 3.3 | 3.3 | 15.0 |
| | 60.70 | 1 | 1.7 | 1.7 | 16.7 |
| | 67.30 | 1 | 1.7 | 1.7 | 18.3 |
| | 69.70 | 1 | 1.7 | 1.7 | 20.0 |
| | 77.80 | 1 | 1.7 | 1.7 | 21.7 |
| | 80.20 | 1 | 1.7 | 1.7 | 23.3 |
| | 87.80 | 1 | 1.7 | 1.7 | 25.0 |
| | 88.60 | 1 | 1.7 | 1.7 | 26.7 |
| | 98.10 | 1 | 1.7 | 1.7 | 28.3 |
| | 108.40 | 1 | 1.7 | 1.7 | 30.0 |
| | 110.10 | 1 | 1.7 | 1.7 | 31.7 |
| | 111.40 | 1 | 1.7 | 1.7 | 33.3 |
| | 112.90 | 1 | 1.7 | 1.7 | 35.0 |
| | 113.80 | 1 | 1.7 | 1.7 | 36.7 |
| | 115.00 | 1 | 1.7 | 1.7 | 38.3 |
| | 115.40 | 1 | 1.7 | 1.7 | 40.0 |
| | 117.30 | 1 | 1.7 | 1.7 | 41.7 |
| | 118.90 | 1 | 1.7 | 1.7 | 43.3 |
| | 120.10 | 1 | 1.7 | 1.7 | 45.0 |
| | 126.40 | 1 | 1.7 | 1.7 | 46.7 |
| | 128.20 | 1 | 1.7 | 1.7 | 48.3 |
| | 130.80 | 1 | 1.7 | 1.7 | 50.0 |
| | 141.70 | 1 | 1.7 | 1.7 | 51.7 |
| | 143.90 | 1 | 1.7 | 1.7 | 53.3 |
| | 145.00 | 1 | 1.7 | 1.7 | 55.0 |
| | 145.70 | 1 | 1.7 | 1.7 | 56.7 |
| | 153.40 | 1 | 1.7 | 1.7 | 58.3 |
| | 153.90 | 1 | 1.7 | 1.7 | 60.0 |
| | 154.70 | 1 | 1.7 | 1.7 | 61.7 |
| | 156.30 | 1 | 1.7 | 1.7 | 63.3 |
| | 156.70 | 1 | 1.7 | 1.7 | 65.0 |
| | 160.10 | 1 | 1.7 | 1.7 | 66.7 |
| | 162.60 | 1 | 1.7 | 1.7 | 68.3 |
| | 163.20 | 1 | 1.7 | 1.7 | 70.0 |
| | 176.00 | 1 | 1.7 | 1.7 | 71.7 |
| | 176.40 | 1 | 1.7 | 1.7 | 73.3 |
| | 181.20 | 1 | 1.7 | 1.7 | 75.0 |
| | 185.20 | 1 | 1.7 | 1.7 | 76.7 |
| | 186.40 | 1 | 1.7 | 1.7 | 78.3 |
| | 186.60 | 1 | 1.7 | 1.7 | 80.0 |
| | 187.30 | 1 | 1.7 | 1.7 | 81.7 |
| | 192.80 | 1 | 1.7 | 1.7 | 83.3 |
| | 193.80 | 1 | 1.7 | 1.7 | 85.0 |
| | 203.80 | 1 | 1.7 | 1.7 | 86.7 |
| | 209.30 | 1 | 1.7 | 1.7 | 88.3 |
| | 214.00 | 1 | 1.7 | 1.7 | 90.0 |
| | 228.70 | 1 | 1.7 | 1.7 | 91.7 |
| | 229.50 | 1 | 1.7 | 1.7 | 93.3 |
| | 241.30 | 1 | 1.7 | 1.7 | 95.0 |
| | 251.40 | 1 | 1.7 | 1.7 | 96.7 |
| | 252.40 | 1 | 1.7 | 1.7 | 98.3 |
| | 257.10 | 1 | 1.7 | 1.7 | 100.0 |
| | Total | 60 | 100.0 | 100.0 | |

**OpenArmsentriesCenterentries Open Arms : entries+Center : entries**

| | | Frequency | Percent | Valid Percent | Cumulative Percent |
|---|---|---|---|---|---|
| Valid | 3 | 1 | 1.7 | 1.7 | 1.7 |
| | 5 | 1 | 1.7 | 1.7 | 3.3 |
| | 6 | 1 | 1.7 | 1.7 | 5.0 |
| | 8 | 1 | 1.7 | 1.7 | 6.7 |
| | 9 | 2 | 3.3 | 3.3 | 10.0 |
| | 10 | 1 | 1.7 | 1.7 | 11.7 |
| | 11 | 1 | 1.7 | 1.7 | 13.3 |
| | 12 | 2 | 3.3 | 3.3 | 16.7 |
| | 13 | 1 | 1.7 | 1.7 | 18.3 |
| | 15 | 1 | 1.7 | 1.7 | 20.0 |
| | 17 | 1 | 1.7 | 1.7 | 21.7 |
| | 19 | 1 | 1.7 | 1.7 | 23.3 |
| | 22 | 2 | 3.3 | 3.3 | 26.7 |
| | 23 | 2 | 3.3 | 3.3 | 30.0 |
| | 24 | 2 | 3.3 | 3.3 | 33.3 |
| | 26 | 1 | 1.7 | 1.7 | 35.0 |
| | 27 | 1 | 1.7 | 1.7 | 36.7 |
| | 28 | 1 | 1.7 | 1.7 | 38.3 |
| | 29 | 2 | 3.3 | 3.3 | 41.7 |
| | 30 | 2 | 3.3 | 3.3 | 45.0 |
| | 31 | 4 | 6.7 | 6.7 | 51.7 |
| | 34 | 1 | 1.7 | 1.7 | 53.3 |
| | 35 | 4 | 6.7 | 6.7 | 60.0 |
| | 36 | 1 | 1.7 | 1.7 | 61.7 |
| | 37 | 1 | 1.7 | 1.7 | 63.3 |
| | 38 | 2 | 3.3 | 3.3 | 66.7 |
| | 39 | 3 | 5.0 | 5.0 | 71.7 |
| | 41 | 2 | 3.3 | 3.3 | 75.0 |
| | 42 | 2 | 3.3 | 3.3 | 78.3 |
| | 43 | 3 | 5.0 | 5.0 | 83.3 |
| | 44 | 1 | 1.7 | 1.7 | 85.0 |
| | 45 | 1 | 1.7 | 1.7 | 86.7 |
| | 50 | 3 | 5.0 | 5.0 | 91.7 |
| | 52 | 1 | 1.7 | 1.7 | 93.3 |
| | 53 | 1 | 1.7 | 1.7 | 95.0 |
| | 58 | 1 | 1.7 | 1.7 | 96.7 |
| | 62 | 1 | 1.7 | 1.7 | 98.3 |
| | 66 | 1 | 1.7 | 1.7 | 100.0 |
| | Total | 60 | 100.0 | 100.0 | |

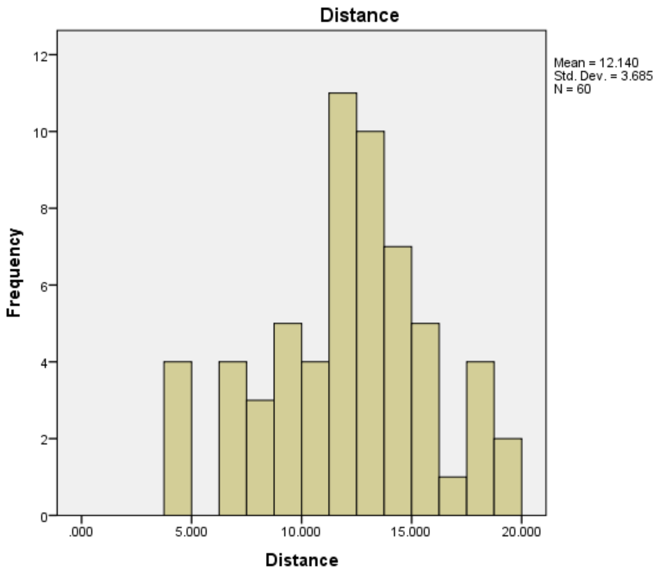
Distance
Mean = 12.140
Std. Dev. = 3.685
N = 60
Frequency
Distance
.000
5.000
10.000
15.000
20.000

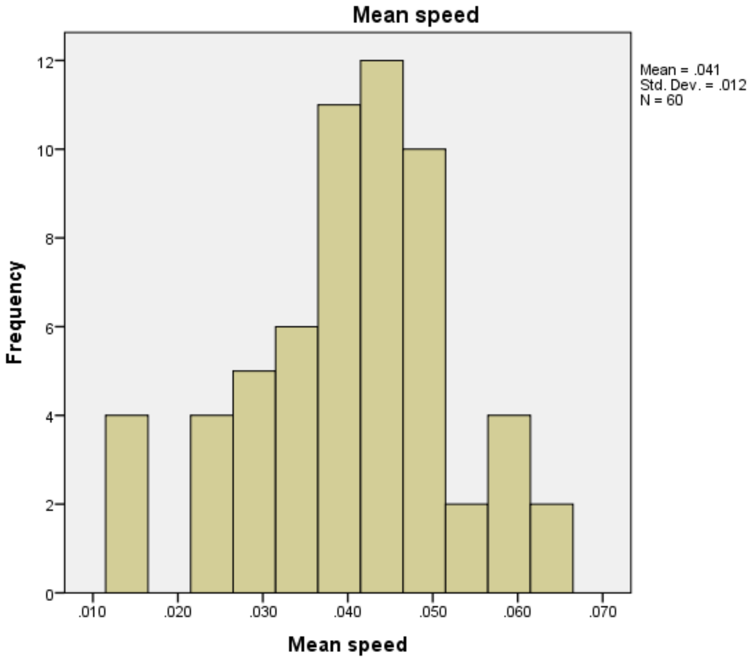
Mean speed
Mean = .041
Std. Dev. = .012
N = 60
Frequency
Mean speed
.010
.020
.030
.040
.050
.060
.070

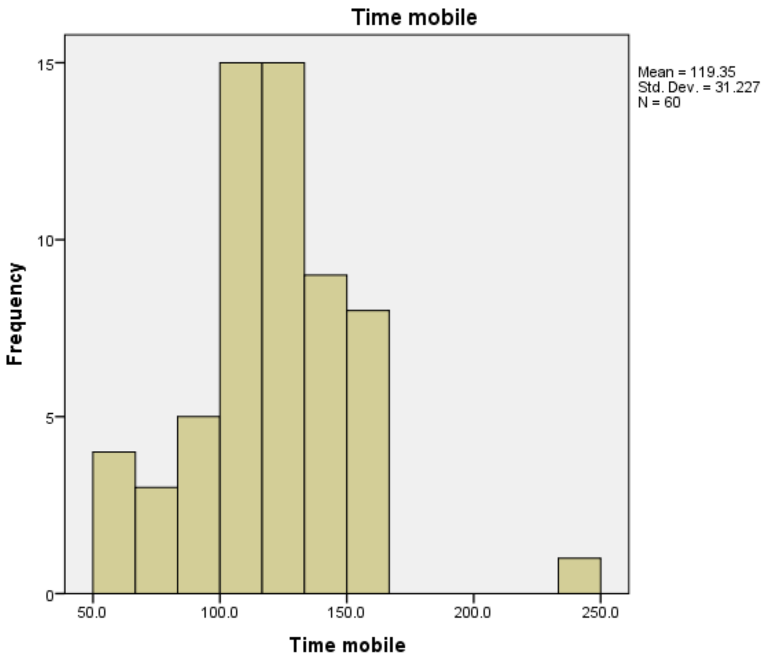

Time mobile
Mean = 119.35
Std. Dev. = 31.227
N = 60
Frequency
0
5
10
15
50.0
100.0
150.0
200.0
250.0
Time mobile


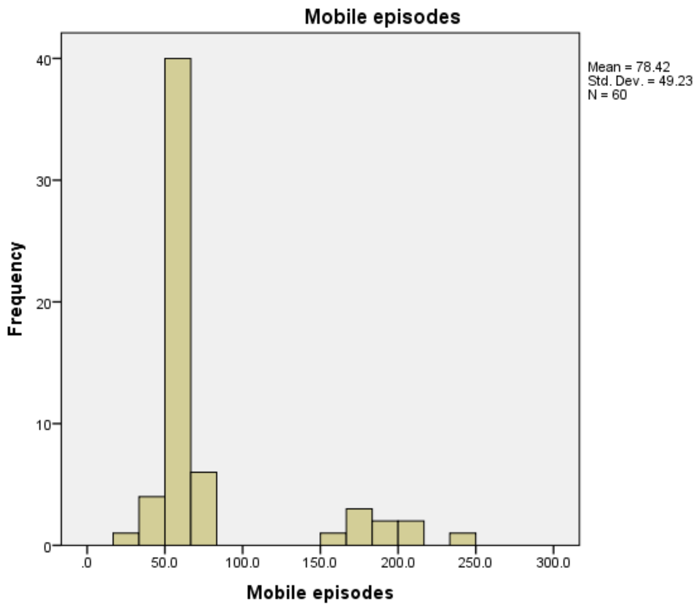

Mobile episodes
Mean = 78.42
Std. Dev. = 49.23
N = 60
Frequency
0
10
20
30
40
.0
50.0
100.0
150.0
200.0
250.0
300.0
Mobile episodes

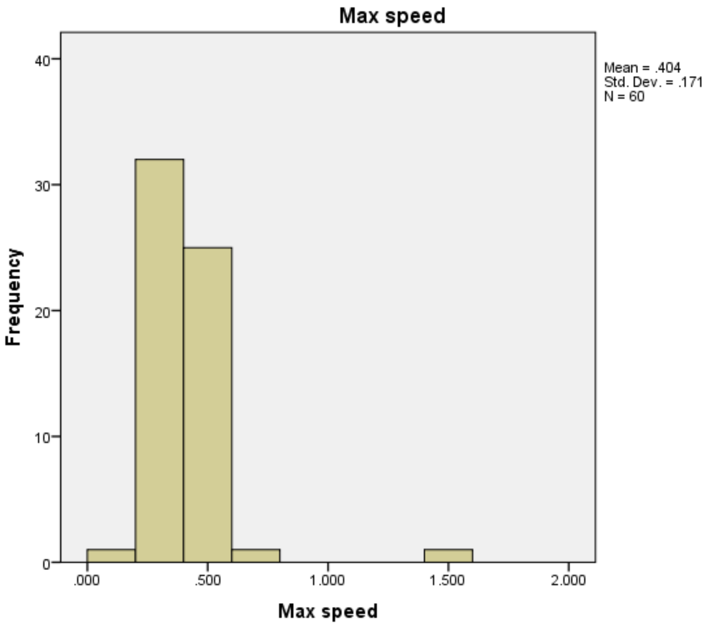
Max speed
Mean = .404
Std. Dev. = .171
N = 60
Frequency
0
10
20
30
40
.000
.500
1.000
1.500
2.000
Max speed

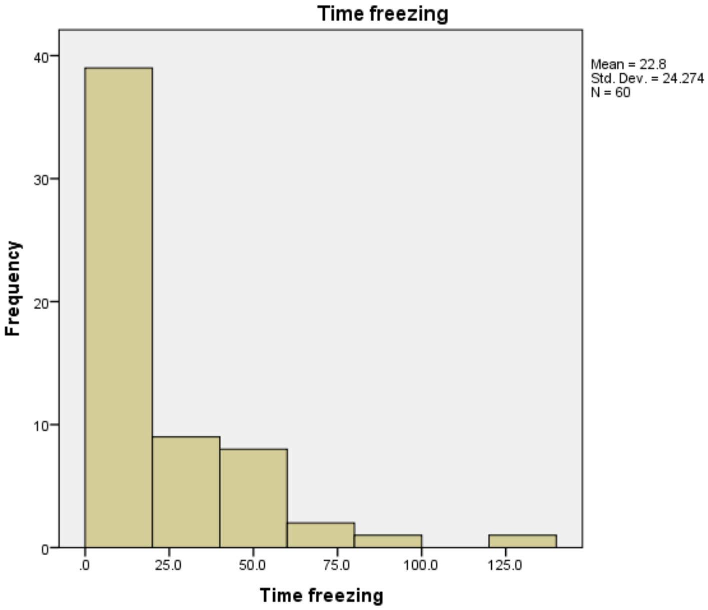
Time freezing
Mean = 22.8
Std. Dev. = 24.274
N = 60
Frequency
0
10
20
30
40
.0
25.0
50.0
75.0
100.0
125.0
Time freezing

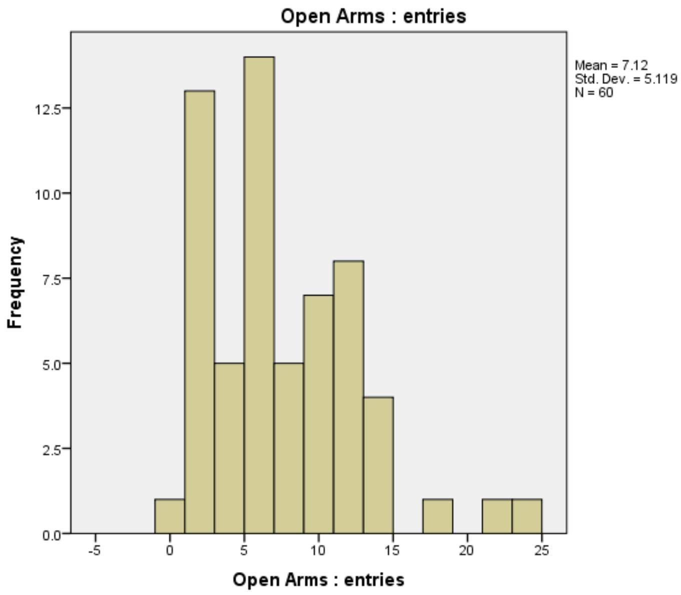
Open Arms : entries
Mean = 7.12
Std. Dev. = 5.119
N = 60
Frequency
12.5
10.0
7.5
5.0
2.5
0.0
-5
0
5
10
15
20
25
Open Arms : entries

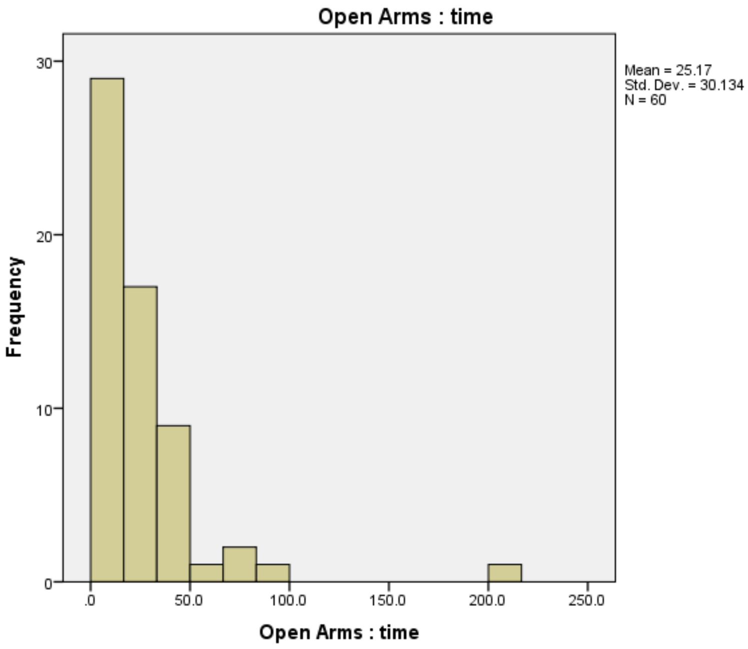
Open Arms : time
Mean = 25.17
Std. Dev. = 30.134
N = 60
Frequency
30
20
10
0
.0
50.0
100.0
150.0
200.0
250.0
Open Arms : time

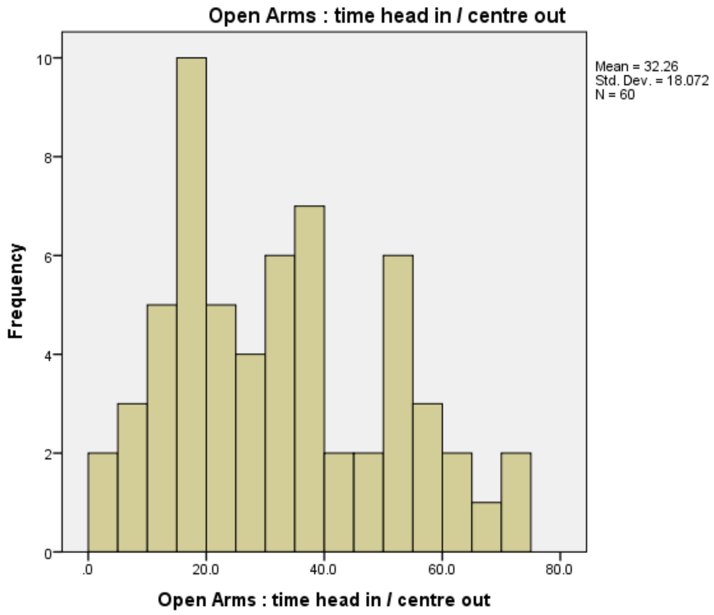

Open Arms : time head in / centre out
Mean = 32.26
Std. Dev. = 18.072
N = 60
Frequency
0
2
4
6
8
10
.0
20.0
40.0
60.0
80.0
Open Arms : time head in / centre out


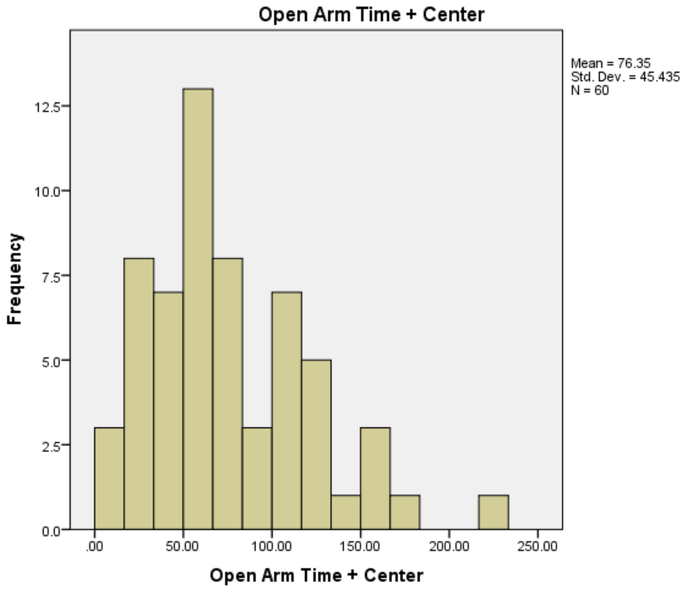

Open Arm Time + Center
Mean = 76.35
Std. Dev. = 45.435
N = 60
Frequency
0.0
2.5
5.0
7.5
10.0
12.5
.00
50.00
100.00
150.00
200.00
250.00
Open Arm Time + Center

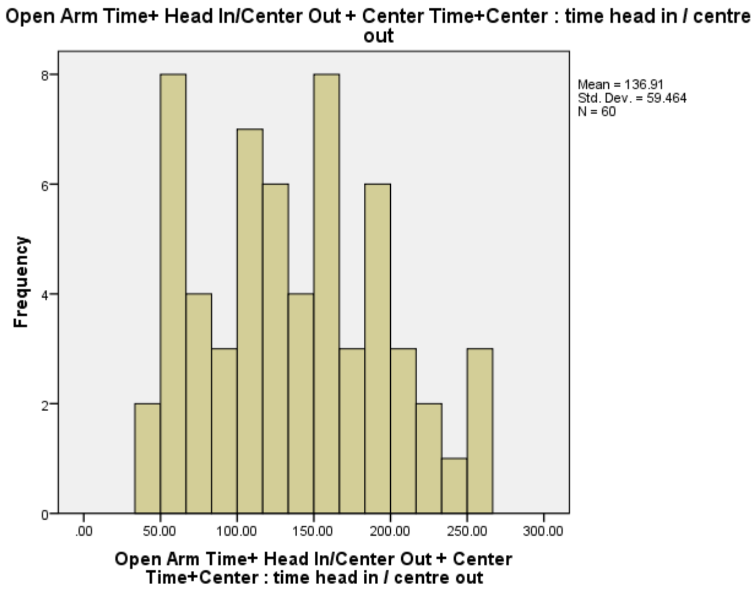
Open Arm Time+ Head In/Center Out + Center Time+Center : time head in / centre out
Mean = 136.91
Std. Dev. = 59.464
N = 60
Frequency
0
2
4
6
8
.00
50.00
100.00
150.00
200.00
250.00
300.00
Open Arm Time+ Head In/Center Out + Center Time+Center : time head in / centre out

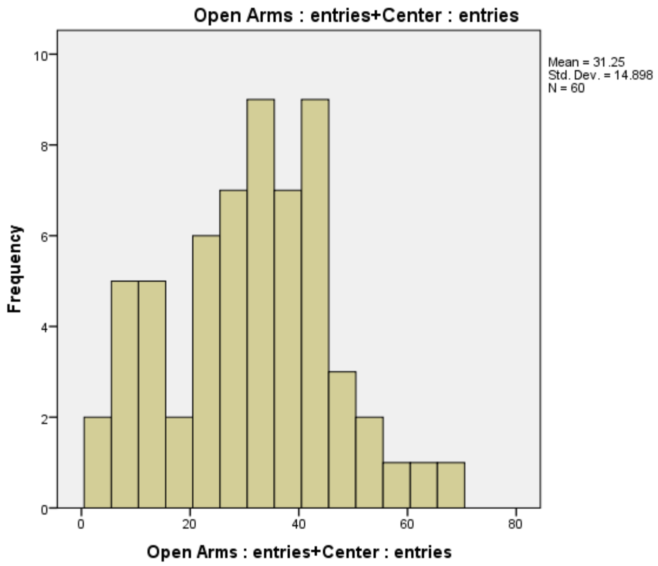
Open Arms : entries+Center : entries
Mean = 31.25
Std. Dev. = 14.898
N = 60
Frequency
0
2
4
6
8
10
0
20
40
60
80
Open Arms : entries+Center : entries

# By Group

**Statistics[a]**

| | | Distance | Meanspeed Mean speed | Timemobile Time mobile | Mobileepisodes Mobile episodes | Maxspeed Max speed | Timefreezing Time freezing | OpenArmsentries Open Arms : entries | OpenArmstime Open Arms : time | OpenArmstimeheadincentreout Open Arms : time head in / centre out | OpenArmTimeCenter Open Arm Time + Center | OpenArmTimeHeadInCenterOutCenterTimeCentertimeheadincentreout Open Arm Time+ Head In/Center Out + Center Time+Center : time head in / centre out | OpenArmsentriesCenterentries Open Arms : entries+Center : entries |
|---|---|---|---|---|---|---|---|---|---|---|---|---|---|
| N | Valid | 10 | 10 | 10 | 10 | 10 | 10 | 10 | 10 | 10 | 10 | 10 | 10 |
| | Missing | 0 | 0 | 0 | 0 | 0 | 0 | 0 | 0 | 0 | 0 | 0 | 0 |
| Mean | | 14.00460 | .04670 | 128.150 | 85.710 | .41150 | 11.440 | 9.60 | 27.530 | 46.530 | 100.9700 | 177.5400 | 42.30 |
| Std. Error of Mean | | .982100 | .003273 | 7.3189 | 13.2702 | .016579 | 2.0925 | 1.360 | 7.6758 | 5.6790 | 11.74317 | 14.35109 | 2.944 |
| Median | | 13.65400 | .04550 | 125.850 | 68.000 | .41000 | 9.700 | 9.50 | 20.200 | 43.700 | 91.7000 | 171.9000 | 43.00 |
| Mode | | 9.010[b] | .030[b] | 87.7[b] | 55.0[b] | .320[b] | 6.0[b] | 8[b] | 3.6[b] | 18.1[b] | 62.60[b] | 118.90[b] | 43 |
| Std. Deviation | | 3.105673 | .010350 | 23.1444 | 41.9640 | .052428 | 6.6170 | 4.300 | 24.2731 | 17.9585 | 37.13516 | 45.38214 | 9.310 |
| Variance | | 9.645 | .000 | 535.663 | 1760.979 | .003 | 43.785 | 18.489 | 589.185 | 322.507 | 1379.020 | 2059.538 | 86.678 |
| Skewness | | .357 | .335 | -.005 | 1.666 | -.465 | 2.199 | -.003 | 1.208 | .063 | .365 | .239 | -.306 |
| Std. Error of Skewness | | .687 | .687 | .687 | .687 | .687 | .687 | .687 | .687 | .687 | .687 | .687 | .687 |
| Kurtosis | | .149 | .083 | -.201 | 1.241 | -.843 | 5.542 | .116 | 1.196 | -.791 | -1.780 | -1.048 | .960 |
| Std. Error of Kurtosis | | 1.334 | 1.334 | 1.334 | 1.334 | 1.334 | 1.334 | 1.334 | 1.334 | 1.334 | 1.334 | 1.334 | 1.334 |
| Range | | 10.587 | .035 | 75.7 | 113.9 | .152 | 22.5 | 15 | 76.8 | 54.8 | 93.50 | 133.50 | 34 |
| Minimum | | 9.010 | .030 | 87.7 | 55.0 | .320 | 6.0 | 2 | 3.6 | 18.1 | 62.60 | 118.90 | 24 |
| Maximum | | 19.597 | .065 | 163.4 | 168.9 | .472 | 28.5 | 17 | 80.4 | 72.9 | 156.10 | 252.40 | 58 |
| Sum | | 140.046 | .467 | 1281.5 | 857.1 | 4.115 | 114.4 | 96 | 275.3 | 465.3 | 1009.70 | 1775.40 | 423 |

a. Group = 1 control-vehicle

b. Multiple modes exist. The smallest value is shown

**Statistics[a]**

| | | Distance | Meanspeed Mean speed | Timemobile Time mobile | Mobileepisodes Mobile episodes | Maxspeed Max speed | Timefreezing Time freezing | OpenArmsentries Open Arms : entries | OpenArmstime Open Arms : time | OpenArmstimeheadincentreout Open Arms : time head in / centre out | OpenArmTimeCenter Open Arm Time + Center | OpenArmTimeHeadInCenterOutCenterTimeCentertimeheadincentreout Open Arm Time+ Head In/Center Out + Center Time+Center : time head in / centre out | OpenArmsentriesCenterentries Open Arms : entries+Center : entries |
|---|---|---|---|---|---|---|---|---|---|---|---|---|---|
| N | Valid | 10 | 10 | 10 | 10 | 10 | 10 | 10 | 10 | 10 | 10 | 10 | 10 |
| | Missing | 0 | 0 | 0 | 0 | 0 | 0 | 0 | 0 | 0 | 0 | 0 | 0 |
| Mean | | 13.03780 | .04340 | 135.030 | 56.700 | .52700 | 12.150 | 8.10 | 43.070 | 40.560 | 107.4200 | 174.5300 | 34.60 |
| Std. Error of Mean | | .727207 | .002437 | 11.6527 | 4.0085 | .117898 | 2.4863 | 1.716 | 19.2517 | 4.3589 | 16.93071 | 15.75173 | 4.440 |
| Median | | 13.06100 | .04350 | 125.050 | 59.500 | .41750 | 9.500 | 6.00 | 20.950 | 40.650 | 92.6500 | 159.7500 | 33.00 |
| Mode | | 8.121[b] | .051 | 108.2[b] | 57.0[b] | .468 | 7.0[b] | 4[b] | 4.6[b] | 18.7[b] | 51.10[b] | 108.40[b] | 31[b] |
| Std. Deviation | | 2.299630 | .007706 | 36.8492 | 12.6759 | .372827 | 7.8623 | 5.425 | 60.8791 | 13.7840 | 53.53960 | 49.81136 | 14.041 |
| Variance | | 5.288 | .000 | 1357.865 | 160.678 | .139 | 61.816 | 29.433 | 3706.265 | 189.998 | 2866.488 | 2481.171 | 197.156 |
| Skewness | | -.935 | -.928 | 2.700 | -2.438 | 3.061 | 1.800 | 2.171 | 2.418 | -.277 | 1.333 | .459 | .947 |
| Std. Error of Skewness | | .687 | .687 | .687 | .687 | .687 | .687 | .687 | .687 | .687 | .687 | .687 | .687 |
| Kurtosis | | 1.093 | .996 | 7.892 | 6.757 | 9.537 | 3.529 | 5.282 | 6.084 | -1.354 | 1.444 | -.807 | 2.708 |
| Std. Error of Kurtosis | | 1.334 | 1.334 | 1.334 | 1.334 | 1.334 | 1.334 | 1.334 | 1.334 | 1.334 | 1.334 | 1.334 | 1.334 |
| Range | | 7.623 | .025 | 127.4 | 44.0 | 1.247 | 27.1 | 18 | 197.9 | 39.1 | 172.90 | 148.70 | 54 |
| Minimum | | 8.121 | .027 | 108.2 | 23.0 | .332 | 4.0 | 4 | 4.6 | 18.7 | 51.10 | 108.40 | 12 |
| Maximum | | 15.744 | .052 | 235.6 | 67.0 | 1.579 | 31.1 | 22 | 202.5 | 57.8 | 224.00 | 257.10 | 66 |
| Sum | | 130.378 | .434 | 1350.3 | 567.0 | 5.270 | 121.5 | 81 | 430.7 | 405.6 | 1074.20 | 1745.30 | 346 |

a. Group = 2 L-Th control drug

b. Multiple modes exist. The smallest value is shown

**Statistics[a]**

| | | Distance | Meanspeed Mean speed | Timemobile Time mobile | Mobileepisodes Mobile episodes | Maxspeed Max speed | Timefreezing Time freezing | OpenArmsentries Open Arms : entries | OpenArmstime Open Arms : time | OpenArmstimeheadincentreout Open Arms : time head in / centre out | OpenArmTimeCenter Open Arm Time + Center | OpenArmTimeHeadInCenterOutCenterTimeCentertimeheadincentreout Open Arm Time+ Head In/Center Out + Center Time+Center : time head in / centre out | OpenArmsentriesCenterentries Open Arms : entries+Center : entries |
|---|---|---|---|---|---|---|---|---|---|---|---|---|---|
| N | Valid | 10 | 10 | 10 | 10 | 10 | 10 | 10 | 10 | 10 | 10 | 10 | 10 |
| | Missing | 0 | 0 | 0 | 0 | 0 | 0 | 0 | 0 | 0 | 0 | 0 | 0 |
| Mean | | 13.75130 | .04600 | 128.800 | 85.500 | .41490 | 8.710 | 10.40 | 28.940 | 39.050 | 92.7300 | 163.2600 | 40.70 |
| Std. Error of Mean | | 1.213471 | .004088 | 7.4126 | 17.8578 | .022851 | 2.2449 | 1.968 | 4.4134 | 5.3391 | 12.54928 | 15.47476 | 3.649 |
| Median | | 13.48500 | .04500 | 124.750 | 60.500 | .43350 | 6.500 | 9.50 | 26.250 | 32.850 | 72.9000 | 142.8000 | 40.00 |
| Mode | | 6.800[b] | .023[b] | 92.8[b] | 58.0 | .251[b] | 5.0[b] | 5 | 13.0[b] | 21.3[b] | 55.80[b] | 112.90[b] | 26[b] |
| Std. Deviation | | 3.837332 | .012927 | 23.4408 | 56.4712 | .072263 | 7.0991 | 6.222 | 13.9565 | 16.8837 | 39.68431 | 48.93547 | 11.538 |
| Variance | | 14.725 | .000 | 549.473 | 3188.998 | .005 | 50.397 | 38.711 | 194.783 | 285.058 | 1574.845 | 2394.680 | 133.122 |
| Skewness | | -.263 | -.219 | .002 | 1.836 | -1.361 | 1.786 | 1.131 | .601 | .830 | .884 | .805 | .444 |
| Std. Error of Skewness | | .687 | .687 | .687 | .687 | .687 | .687 | .687 | .687 | .687 | .687 | .687 | .687 |
| Kurtosis | | -.505 | -.608 | -1.343 | 1.891 | 2.155 | 4.124 | 1.215 | -1.345 | -.877 | -.854 | -.705 | -.340 |
| Std. Error of Kurtosis | | 1.334 | 1.334 | 1.334 | 1.334 | 1.334 | 1.334 | 1.334 | 1.334 | 1.334 | 1.334 | 1.334 | 1.334 |
| Range | | 11.972 | .040 | 67.8 | 155.2 | .248 | 26.1 | 19 | 35.4 | 47.3 | 103.70 | 138.50 | 36 |
| Minimum | | 6.800 | .023 | 92.8 | 52.0 | .251 | .0 | 5 | 13.0 | 21.3 | 55.80 | 112.90 | 26 |
| Maximum | | 18.772 | .063 | 160.6 | 207.2 | .499 | 26.1 | 24 | 48.4 | 68.6 | 159.50 | 251.40 | 62 |
| Sum | | 137.513 | .460 | 1288.0 | 855.0 | 4.149 | 87.1 | 104 | 289.4 | 390.5 | 927.30 | 1632.60 | 407 |

a. Group = 3 control-naïve

b. Multiple modes exist. The smallest value is shown

**Statistics[a]**

| | | Distance | Meanspeed Mean speed | Timemobile Time mobile | Mobileepisodes Mobile episodes | Maxspeed Max speed | Timefreezing Time freezing | OpenArmsentries Open Arms : entries | OpenArmstime Open Arms : time | OpenArmstimeheadincentreout Open Arms : time head in / centre out | OpenArmTimeCenter Open Arm Time + Center | OpenArmTimeHeadInCenterOutCenterTimeCentertimeheadincentreout Open Arm Time+ Head In/Center Out + Center Time+Center : time head in / centre out | OpenArmsentriesCenterentries Open Arms : entries+Center : entries |
|---|---|---|---|---|---|---|---|---|---|---|---|---|---|
| N | Valid | 10 | 10 | 10 | 10 | 10 | 10 | 10 | 10 | 10 | 10 | 10 | 10 |
| | Missing | 0 | 0 | 0 | 0 | 0 | 0 | 0 | 0 | 0 | 0 | 0 | 0 |
| Mean | | 11.60550 | .03890 | 115.080 | 84.690 | .39000 | 38.040 | 5.00 | 18.620 | 20.330 | 54.7000 | 96.2200 | 24.90 |
| Std. Error of Mean | | 1.198202 | .003998 | 10.8465 | 18.8252 | .031433 | 9.1748 | 1.445 | 7.4141 | 4.6087 | 13.49651 | 17.49405 | 4.540 |
| Median | | 12.20350 | .04050 | 118.000 | 59.000 | .38650 | 31.600 | 2.50 | 9.950 | 17.000 | 36.2000 | 78.7500 | 22.50 |
| Mode | | 4.385[b] | .015[b] | 54.2[b] | 47.0 | .280[b] | 7.0[b] | 2 | .0[b] | 6.1[b] | 13.00[b] | 43.00[b] | 23 |
| Std. Deviation | | 3.789046 | .012644 | 34.2996 | 59.5304 | .099400 | 29.0134 | 4.570 | 23.4456 | 14.5741 | 42.67971 | 55.32104 | 14.356 |
| Variance | | 14.357 | .000 | 1176.460 | 3543.872 | .010 | 841.778 | 20.889 | 549.695 | 212.405 | 1821.558 | 3060.417 | 206.100 |
| Skewness | | -.660 | -.627 | -.461 | 1.732 | 1.736 | .705 | .829 | 1.615 | 1.353 | .872 | .819 | .881 |
| Std. Error of Skewness | | .687 | .687 | .687 | .687 | .687 | .687 | .687 | .687 | .687 | .687 | .687 | .687 |
| Kurtosis | | -.052 | -.104 | -.260 | 1.331 | 4.125 | -.778 | -1.142 | 1.427 | 1.054 | -1.210 | -1.150 | .078 |
| Std. Error of Kurtosis | | 1.334 | 1.334 | 1.334 | 1.334 | 1.334 | 1.334 | 1.334 | 1.334 | 1.334 | 1.334 | 1.334 | 1.334 |
| Range | | 12.319 | .041 | 112.4 | 153.5 | .354 | 81.4 | 12 | 68.1 | 44.3 | 107.00 | 142.20 | 45 |
| Minimum | | 4.385 | .015 | 54.2 | 47.0 | .280 | 7.0 | 0 | .0 | 6.1 | 13.00 | 43.00 | 8 |
| Maximum | | 16.704 | .056 | 166.6 | 200.5 | .634 | 88.4 | 12 | 68.1 | 50.4 | 120.00 | 185.20 | 53 |
| Sum | | 116.055 | .389 | 1150.8 | 846.9 | 3.900 | 380.4 | 50 | 186.2 | 203.3 | 547.00 | 962.20 | 249 |

a. Group = 4 PTSD-vehicle

b. Multiple modes exist. The smallest value is shown

Statistics[a]

| | | Distance | Meanspeed Mean speed | Timemobile Time mobile | Mobileepisodes Mobile episodes | Maxspeed Max speed | Timefreezing Time freezing | OpenArmsentries Open Arms : entries | OpenArmstime Open Arms : time | OpenArmstimeheadincentreout Open Arms : time head in / centre out | OpenArmTimeCenter Open Arm Time + Center | OpenArmTimeHeadInCenterOutCenterTimeCentertimeheadincentreout Open Arm Time+ Head In/Center Out + Center Time+Center : time head in / centre out | OpenArmsentriesCenterentries Open Arms : entries+Center : entries |
|---|---|---|---|---|---|---|---|---|---|---|---|---|---|
| N | Valid | 10 | 10 | 10 | 10 | 10 | 10 | 10 | 10 | 10 | 10 | 10 | 10 |
| | Missing | 0 | 0 | 0 | 0 | 0 | 0 | 0 | 0 | 0 | 0 | 0 | 0 |
| Mean | | 10.51410 | .03520 | 107.390 | 57.800 | .34830 | 42.900 | 5.40 | 15.900 | 27.140 | 56.7600 | 109.7100 | 25.30 |
| Std. Error of Mean | | 1.104305 | .003729 | 9.5917 | 3.1333 | .016810 | 11.7793 | 1.240 | 4.2443 | 5.3407 | 11.69005 | 18.05179 | 4.789 |
| Median | | 11.55650 | .03850 | 115.750 | 62.000 | .34750 | 43.450 | 5.50 | 14.850 | 20.700 | 45.8000 | 97.0000 | 30.50 |
| Mode | | 4.452[b] | .045 | 54.5[b] | 65.0 | .270[b] | 11.0[b] | 1 | .9[b] | 6.5[b] | 15.00[b] | 51.30[b] | 9 |
| Std. Deviation | | 3.492119 | .011793 | 30.3318 | 9.9085 | .053158 | 37.2494 | 3.921 | 13.4217 | 16.8889 | 36.96717 | 57.08476 | 15.144 |
| Variance | | 12.195 | .000 | 920.017 | 98.178 | .003 | 1387.516 | 15.378 | 180.142 | 285.236 | 1366.572 | 3258.670 | 229.344 |
| Skewness | | -.953 | -.937 | -.420 | -1.723 | -.115 | 1.650 | -.015 | .754 | .716 | .595 | .547 | -.197 |
| Std. Error of Skewness | | .687 | .687 | .687 | .687 | .687 | .687 | .687 | .687 | .687 | .687 | .687 | .687 |
| Kurtosis | | -.394 | -.411 | -.347 | 2.437 | -1.201 | 3.483 | -2.041 | -.155 | -.697 | -1.290 | -1.517 | -1.935 |
| Std. Error of Kurtosis | | 1.334 | 1.334 | 1.334 | 1.334 | 1.334 | 1.334 | 1.334 | 1.334 | 1.334 | 1.334 | 1.334 | 1.334 |
| Range | | 9.524 | .032 | 98.3 | 30.0 | .154 | 121.7 | 9 | 40.9 | 47.8 | 96.40 | 142.50 | 39 |
| Minimum | | 4.452 | .015 | 54.5 | 35.0 | .270 | 11.0 | 1 | .9 | 6.5 | 15.00 | 51.30 | 6 |
| Maximum | | 13.976 | .047 | 152.8 | 65.0 | .424 | 132.7 | 10 | 41.8 | 54.3 | 111.40 | 193.80 | 45 |
| Sum | | 105.141 | .352 | 1073.9 | 578.0 | 3.483 | 429.0 | 54 | 159.0 | 271.4 | 567.60 | 1097.10 | 253 |

a. Group = 5 PTSD-drug pre-treatment

b. Multiple modes exist. The smallest value is shown

Statistics[a]

| | | Distance | Meanspeed Mean speed | Timemobile Time mobile | Mobileepisodes Mobile episodes | Maxspeed Max speed | Timefreezing Time freezing | OpenArmsentries Open Arms : entries | OpenArmstime Open Arms : time | OpenArmstimeheadincentreout Open Arms : time head in / centre out | OpenArmTimeCenter Open Arm Time + Center | OpenArmTimeHeadInCenterOutCenterTimeCentertimeheadincentreout Open Arm Time+ Head In/Center Out + Center Time+Center : time head in / centre out | OpenArmsentriesCenterentries Open Arms : entries+Center : entries |
|---|---|---|---|---|---|---|---|---|---|---|---|---|---|
| N | Valid | 10 | 10 | 10 | 10 | 10 | 10 | 10 | 10 | 10 | 10 | 10 | 10 |
| | Missing | 0 | 0 | 0 | 0 | 0 | 0 | 0 | 0 | 0 | 0 | 0 | 0 |
| Mean | | 9.92370 | .03290 | 101.630 | 100.140 | .33300 | 23.550 | 4.20 | 16.960 | 19.930 | 45.5300 | 100.1900 | 19.70 |
| Std. Error of Mean | | 1.283369 | .004259 | 9.3094 | 23.3581 | .022467 | 5.2912 | 1.062 | 3.6975 | 3.6810 | 6.90929 | 12.60083 | 3.506 |
| Median | | 9.01800 | .03000 | 107.750 | 58.000 | .33250 | 23.200 | 3.50 | 13.250 | 18.850 | 49.4000 | 104.1000 | 19.50 |
| Mode | | 4.312[b] | .022[b] | 55.7[b] | 51.0 | .194[b] | 4.0[b] | 1[b] | 1.9[b] | 3.6[b] | 19.50[b] | 43.40[b] | 3[b] |
| Std. Deviation | | 4.058368 | .013470 | 29.4388 | 73.8647 | .071046 | 16.7324 | 3.360 | 11.6925 | 11.6405 | 21.84908 | 39.84733 | 11.086 |
| Variance | | 16.470 | .000 | 866.645 | 5455.998 | .005 | 279.972 | 11.289 | 136.714 | 135.500 | 477.382 | 1587.810 | 122.900 |
| Skewness | | .734 | .711 | -.024 | 1.167 | -.317 | 1.007 | 1.498 | .837 | .016 | .040 | .153 | -.204 |
| Std. Error of Skewness | | .687 | .687 | .687 | .687 | .687 | .687 | .687 | .687 | .687 | .687 | .687 | .687 |
| Kurtosis | | .453 | .454 | -.029 | -.535 | .807 | .682 | 2.550 | -.088 | -1.160 | -1.620 | -1.102 | -1.278 |
| Std. Error of Kurtosis | | 1.334 | 1.334 | 1.334 | 1.334 | 1.334 | 1.334 | 1.334 | 1.334 | 1.334 | 1.334 | 1.334 | 1.334 |
| Range | | 13.795 | .046 | 98.5 | 186.6 | .255 | 53.5 | 11 | 37.5 | 33.8 | 59.70 | 116.70 | 32 |
| Minimum | | 4.312 | .014 | 55.7 | 51.0 | .194 | 4.0 | 1 | 1.9 | 3.6 | 19.50 | 43.40 | 3 |
| Maximum | | 18.107 | .060 | 154.2 | 237.6 | .449 | 57.5 | 12 | 39.4 | 37.4 | 79.20 | 160.10 | 35 |
| Sum | | 99.237 | .329 | 1016.3 | 1001.4 | 3.330 | 235.5 | 42 | 169.6 | 199.3 | 455.30 | 1001.90 | 197 |

a. Group = 6 PTSD-post-treatment

b. Multiple modes exist. The smallest value is shown

## One-Way ANOVA

### Mean Speed

**Descriptive Statistics**

Dependent Variable: Meanspeed Mean speed

| Group | Mean | Std. Deviation | N |
|---|---|---|---|
| 1 control-vehicle | .04670 | .010350 | 10 |
| 2 L-Th control drug | .04340 | .007706 | 10 |
| 3 control-naïve | .04600 | .012927 | 10 |
| 4 PTSD-vehicle | .03890 | .012644 | 10 |
| 5 PTSD-drug pre-treatment | .03520 | .011793 | 10 |
| 6 PTSD-post-treatment | .03290 | .013470 | 10 |
| Total | .04052 | .012338 | 60 |

**Levene's Test of Equality of Error Variances[a]**

Dependent Variable: Meanspeed Mean speed

| F | df1 | df2 | Sig. |
|---|---|---|---|
| .786 | 5 | 54 | .564 |

Tests the null hypothesis that the error variance of the dependent variable is equal across groups.

a. Design: Intercept + Group

**Tests of Between-Subjects Effects**

Dependent Variable: Meanspeed Mean speed

| Source | Type III Sum of Squares | df | Mean Square | F | Sig. | Partial Eta Squared |
|---|---|---|---|---|---|---|
| Corrected Model | .002[a] | 5 | .000 | 2.440 | .046 | .184 |
| Intercept | .098 | 1 | .098 | 726.025 | .000 | .931 |
| Group | .002 | 5 | .000 | 2.440 | .046 | .184 |
| Error | .007 | 54 | .000 | | | |
| Total | .107 | 60 | | | | |
| Corrected Total | .009 | 59 | | | | |

a. R Squared = .184 (Adjusted R Squared = .109)

**Multiple Comparisons**

Dependent Variable: Meanspeed Mean speed

Tukey HSD

| (I) Group | (J) Group | Mean Difference (I-J) | Std. Error | Sig. | 95% Confidence Interval Lower Bound | 95% Confidence Interval Upper Bound |
|---|---|---|---|---|---|---|
| 1 control-vehicle | 2 L-Th control drug | .00330 | .005209 | .988 | -.01209 | .01869 |
| | 3 control-naïve | .00070 | .005209 | 1.000 | -.01469 | .01609 |
| | 4 PTSD-vehicle | .00780 | .005209 | .667 | -.00759 | .02319 |
| | 5 PTSD-drug pre-treatment | .01150 | .005209 | .251 | -.00389 | .02689 |
| | 6 PTSD-post-treatment | .01380 | .005209 | .103 | -.00159 | .02919 |
| 2 L-Th control drug | 1 control-vehicle | -.00330 | .005209 | .988 | -.01869 | .01209 |
| | 3 control-naïve | -.00260 | .005209 | .996 | -.01799 | .01279 |
| | 4 PTSD-vehicle | .00450 | .005209 | .953 | -.01089 | .01989 |
| | 5 PTSD-drug pre-treatment | .00820 | .005209 | .619 | -.00719 | .02359 |
| | 6 PTSD-post-treatment | .01050 | .005209 | .347 | -.00489 | .02589 |
| 3 control-naïve | 1 control-vehicle | -.00070 | .005209 | 1.000 | -.01609 | .01469 |
| | 2 L-Th control drug | .00260 | .005209 | .996 | -.01279 | .01799 |
| | 4 PTSD-vehicle | .00710 | .005209 | .748 | -.00829 | .02249 |
| | 5 PTSD-drug pre-treatment | .01080 | .005209 | .316 | -.00459 | .02619 |
| | 6 PTSD-post-treatment | .01310 | .005209 | .138 | -.00229 | .02849 |
| 4 PTSD-vehicle | 1 control-vehicle | -.00780 | .005209 | .667 | -.02319 | .00759 |
| | 2 L-Th control drug | -.00450 | .005209 | .953 | -.01989 | .01089 |
| | 3 control-naïve | -.00710 | .005209 | .748 | -.02249 | .00829 |
| | 5 PTSD-drug pre-treatment | .00370 | .005209 | .980 | -.01169 | .01909 |
| | 6 PTSD-post-treatment | .00600 | .005209 | .857 | -.00939 | .02139 |
| 5 PTSD-drug pre-treatment | 1 control-vehicle | -.01150 | .005209 | .251 | -.02689 | .00389 |
| | 2 L-Th control drug | -.00820 | .005209 | .619 | -.02359 | .00719 |
| | 3 control-naïve | -.01080 | .005209 | .316 | -.02619 | .00459 |
| | 4 PTSD-vehicle | -.00370 | .005209 | .980 | -.01909 | .01169 |
| | 6 PTSD-post-treatment | .00230 | .005209 | .998 | -.01309 | .01769 |
| 6 PTSD-post-treatment | 1 control-vehicle | -.01380 | .005209 | .103 | -.02919 | .00159 |
| | 2 L-Th control drug | -.01050 | .005209 | .347 | -.02589 | .00489 |
| | 3 control-naïve | -.01310 | .005209 | .138 | -.02849 | .00229 |
| | 4 PTSD-vehicle | -.00600 | .005209 | .857 | -.02139 | .00939 |
| | 5 PTSD-drug pre-treatment | -.00230 | .005209 | .998 | -.01769 | .01309 |

Based on observed means.
The error term is Mean Square(Error) = .000.

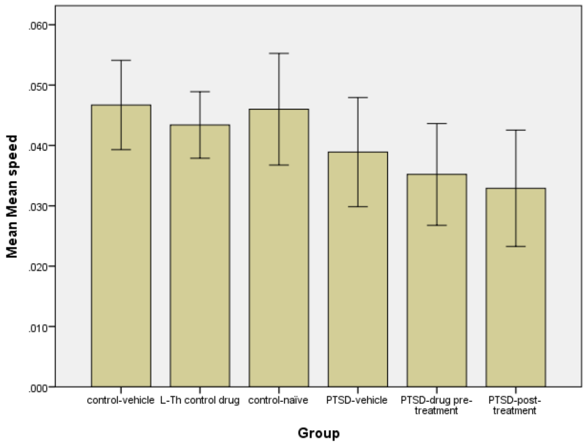


Error bars: 95% CI

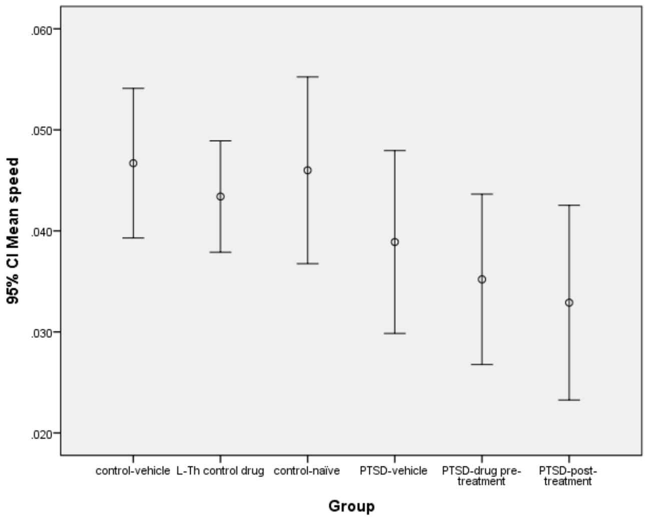


**Summary:** For the **Mean Speed** outcome, there was a significant difference between the six groups: $F(5, 54) = 2.44$, $p = .046$ ($\eta^2 = .184$). The control-vehicle group has the highest mean ($M = .0467$) and PTSD-post-treatment the lowest ($M = .0329$). Per the Tukey HSD post hoc test there were no significant pairwise differences.

## Time Mobile

**Descriptive Statistics**

Dependent Variable: Timemobile Time mobile

| Group | Mean | Std. Deviation | N |
|---|---|---|---|
| 1 control-vehicle | 128.150 | 23.1444 | 10 |
| 2 L-Th control drug | 135.030 | 36.8492 | 10 |
| 3 control-naïve | 128.800 | 23.4408 | 10 |
| 4 PTSD-vehicle | 115.080 | 34.2996 | 10 |
| 5 PTSD-drug pre-treatment | 107.390 | 30.3318 | 10 |
| 6 PTSD-post-treatment | 101.630 | 29.4388 | 10 |
| Total | 119.347 | 31.2274 | 60 |

**Levene's Test of Equality of Error Variances[a]**

Dependent Variable: Timemobile Time mobile

| F | df1 | df2 | Sig. |
|---|---|---|---|
| .285 | 5 | 54 | .919 |

Tests the null hypothesis that the error variance of the dependent variable is equal across groups.

a. Design: Intercept + Group

**Tests of Between-Subjects Effects**

Dependent Variable: Timemobile Time mobile

| Source | Type III Sum of Squares | df | Mean Square | F | Sig. | Partial Eta Squared |
|---|---|---|---|---|---|---|
| Corrected Model | 8878.777[a] | 5 | 1775.755 | 1.971 | .098 | .154 |
| Intercept | 854617.611 | 1 | 854617.611 | 948.500 | .000 | .946 |
| Group | 8878.777 | 5 | 1775.755 | 1.971 | .098 | .154 |
| Error | 48655.092 | 54 | 901.020 | | | |
| Total | 912151.480 | 60 | | | | |
| Corrected Total | 57533.869 | 59 | | | | |

a. R Squared = .154 (Adjusted R Squared = .076)

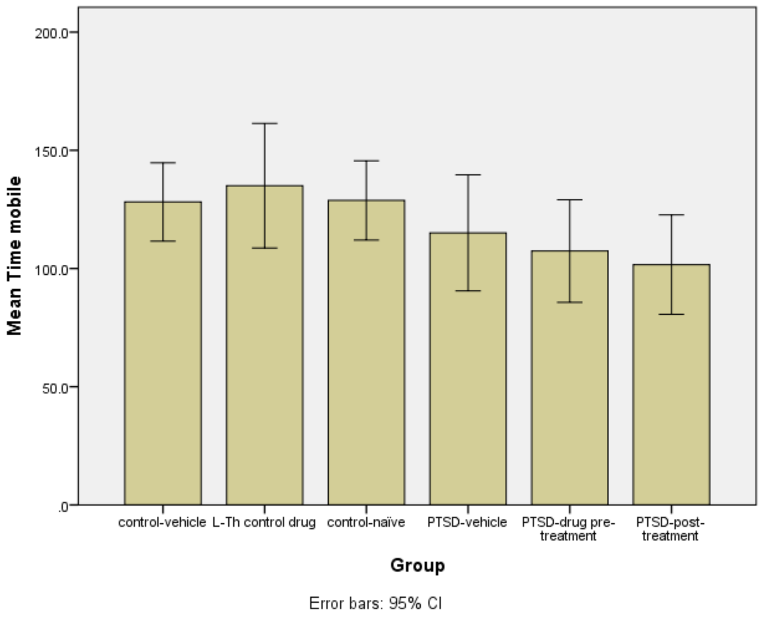


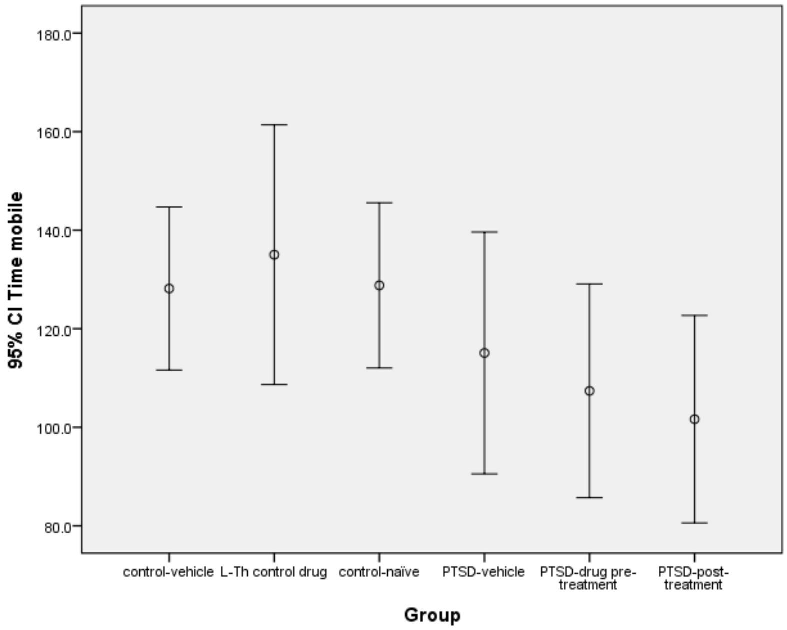


**Summary:** For the **Time Mobile** outcome, there was not a significant difference between the six groups: $F(5, 54) = 1.97$, $p = .098$ ($\eta^2 = .154$). Though not significant the PGB control drug group has the highest mean ($M = 135.03$) and PTSD-post-treatment the lowest ($M = 101.63$).

## Open Arms Time

OpenArmTimeHeadInCenterOutCenterTimeCentertimeheadincentreout

**Report**

OpenArmTimeHeadInCenterOutCenterTimeCentertimeheadincentreout

| Group | Mean | N | Std. Deviation | Std. Error of Mean |
|---|---|---|---|---|
| 1 | 177.54 | 10 | 45.382 | 14.351 |
| 2 | 174.53 | 10 | 49.811 | 15.752 |
| 3 | 163.26 | 10 | 48.935 | 15.475 |
| 4 | 96.22 | 10 | 55.321 | 17.494 |
| 5 | 109.71 | 10 | 57.085 | 18.052 |
| 6 | 100.19 | 10 | 39.847 | 12.601 |
| Total | 136.91 | 60 | 59.464 | 7.677 |

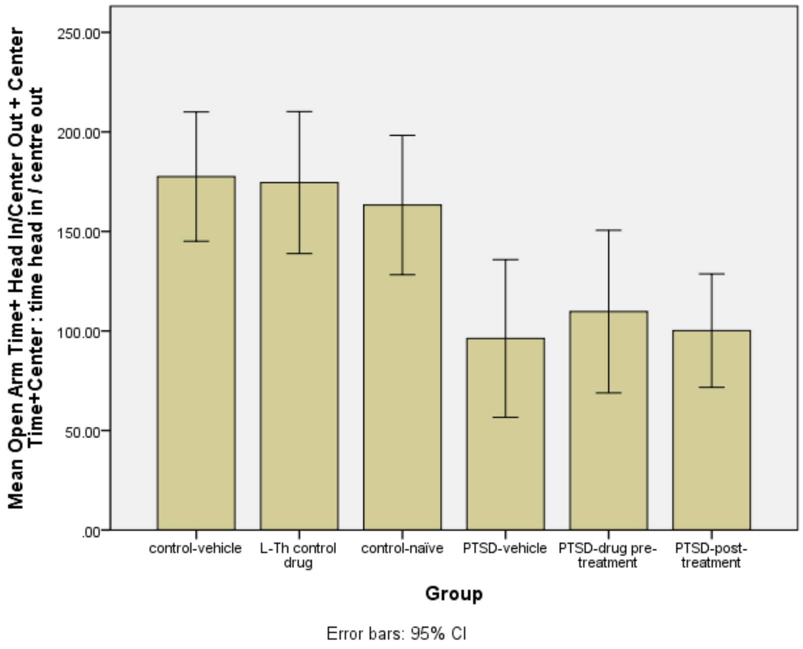

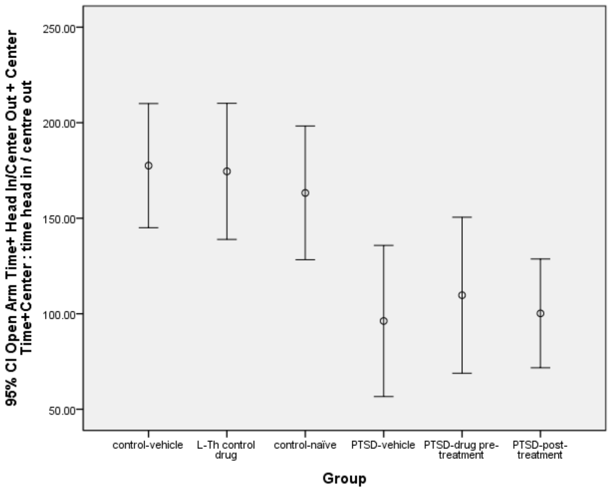


OpenArmTimeHeadInCenterOutCenterTimeCentertimeheadincentreout

**Multiple Comparisons**

Dependent Variable: Ratio18

Tukey HSD

| (I) Group | (J) Group | Mean Difference (I-J) | Std. Error | Sig. | 95% Confidence Interval Lower Bound | Upper Bound |
|---|---|---|---|---|---|---|
| 1 | 2 | 1.00333 | 7.41427 | 1.000 | -20.9020 | 22.9087 |
| | 3 | 4.76000 | 7.41427 | .987 | -17.1453 | 26.6653 |
| | 4 | 27.10667* | 7.41427 | .007 | 5.2013 | 49.0120 |
| | 5 | 22.61000* | 7.41427 | .039 | .7047 | 44.5153 |
| | 6 | 25.78333* | 7.41427 | .012 | 3.8780 | 47.6887 |
| 2 | 1 | -1.00333 | 7.41427 | 1.000 | -22.9087 | 20.9020 |
| | 3 | 3.75667 | 7.41427 | .996 | -18.1487 | 25.6620 |
| | 4 | 26.10333* | 7.41427 | .011 | 4.1980 | 48.0087 |
| | 5 | 21.60667 | 7.41427 | .055 | -.2987 | 43.5120 |
| | 6 | 24.78000* | 7.41427 | .018 | 2.8747 | 46.6853 |
| 3 | 1 | -4.76000 | 7.41427 | .987 | -26.6653 | 17.1453 |
| | 2 | -3.75667 | 7.41427 | .996 | -25.6620 | 18.1487 |
| | 4 | 22.34667* | 7.41427 | .043 | .4413 | 44.2520 |
| | 5 | 17.85000 | 7.41427 | .172 | -4.0553 | 39.7553 |
| | 6 | 21.02333 | 7.41427 | .067 | -.8820 | 42.9287 |
| 4 | 1 | -27.10667* | 7.41427 | .007 | -49.0120 | -5.2013 |
| | 2 | -26.10333* | 7.41427 | .011 | -48.0087 | -4.1980 |
| | 3 | -22.34667* | 7.41427 | .043 | -44.2520 | -.4413 |
| | 5 | -4.49667 | 7.41427 | .990 | -26.4020 | 17.4087 |

| | | | | | | |
|---|---|---|---|---|---|---|
| | 6 | -1.32333 | 7.41427 | 1.000 | -23.2287 | 20.5820 |
| 5 | 1 | -22.61000* | 7.41427 | .039 | -44.5153 | -.7047 |
| | 2 | -21.60667 | 7.41427 | .055 | -43.5120 | .2987 |
| | 3 | -17.85000 | 7.41427 | .172 | -39.7553 | 4.0553 |
| | 4 | 4.49667 | 7.41427 | .990 | -17.4087 | 26.4020 |
| | 6 | 3.17333 | 7.41427 | .998 | -18.7320 | 25.0787 |
| 6 | 1 | -25.78333* | 7.41427 | .012 | -47.6887 | -3.8780 |
| | 2 | -24.78000* | 7.41427 | .018 | -46.6853 | -2.8747 |
| | 3 | -21.02333 | 7.41427 | .067 | -42.9287 | .8820 |
| | 4 | 1.32333 | 7.41427 | 1.000 | -20.5820 | 23.2287 |
| | 5 | -3.17333 | 7.41427 | .998 | -25.0787 | 18.7320 |

*. The mean difference is significant at the 0.05 level.

**Report**

Ratio18

| Group | Mean | N | Std. Deviation | Std. Error of Mean |
|---|---|---|---|---|
| 1 | 59.1800 | 10 | 15.12738 | 4.78370 |
| 2 | 58.1767 | 10 | 16.60379 | 5.25058 |
| 3 | 54.4200 | 10 | 16.31182 | 5.15825 |
| 4 | 32.0733 | 10 | 18.44035 | 5.83135 |
| 5 | 36.5700 | 10 | 19.02825 | 6.01726 |
| 6 | 33.3967 | 10 | 13.28244 | 4.20028 |
| Total | 45.6361 | 60 | 19.82139 | 2.55893 |

**Summary:** For the **OpenArmTimeHeadInCenterOutCenter** outcome, there was a significant difference between the six groups: $F(5, 54) = 6.07$, $p < .001$ ($\eta^2 = .36$). The control-vehicle group has the highest mean ($M = 177.54$) and PTSD-vehicle lowest ($M = 96.22$). Per the Tukey HSD post hoc tests the control vehicle group had a significantly higher mean than the PTSD-vehicle, PTSD-drug pre- treatment, and PTSD-post-treatment groups. Moreover, the PTSD-vehicle group has a significantly lower mean than the both the PGB control drug and control naïve groups.
