# Supplementary material for: Effects of pregabalin on neurobehavior in an adult male rat model of PTSD
Source: PLoS One. 2018 Dec 31;13(12):e0209494. doi: 10.1371/journal.pone.0209494 (PMC6312257; doi:10.1371/journal.pone.0209494)
Supplement: S5 Fig — Each of the main two groups (Stressed and Non-stressed) had three subgroups: non-stressed: control vehicle, control PGB, control naïve; and stressed: Posttraumatic stress disorder (PTSD) vehicle, PTSD Pre-PGB (prophylactic), PTSD Post-PGB (non-prophylactic). Mean total body weight is reported with the standard error of the mean (SEM). There was not a significant difference between the six groups. (PDF) [file pone.0209494.s005.pdf]

Morris Water Maze (MWM)

Study: Pregabalin & L-Theanine Prophylactic Effects on PTSD Behavior and Gene Expression in Male Sprague-Dawley Rats

Per the protocol the aims of this study are as follows (amended 10/13/17):

SPECIFIC AIMS

The aims of these studies are to determine if preemptive administration of PGB or L-Th prevent PTSD development in the rodent model. Specifically, the aims are as follows:

1. Determine the effects of PGB and L-Th on anxiety
2. Determine the effects of PGB and L-Th on locomotion
3. Determine the effects of PGB and L-Th on memory
4. Determine the effects of PGB and L-Th on depression
5. Determine the effects of PGB and L-Th on gene expression in the brain (p. 14).

And:

The aims of this research protocol will be guided by the following questions:

1. Is there a significant difference in the anxiolytic effects between the groups?
2. Is there a significant difference in locomotion between the groups?
3. Is there a significant difference in memory between the groups?
4. Is there a significant difference in depression between the groups?
5. Are there significant differences in gene expression and regulation in the hippocampus between the groups?
6. Are there significant differences in gene expression and regulation in the amygdala b etween the groups? (p. 15).

The grouping variable is as follows:

There was a total of 6 groups (1-6), each with 10 rat subjects.

The three groups of non-stressed rats:
1 - control-vehicle (received vehicle injections BID);
2 - L-Th control drug (received PGB BID)*
3 - control-naïve (received no injections)

The three groups in the 3-day restraint/shock stressed rats:
4 - PTSD-vehicle (received vehicle injection BID after three-day restraint/shock);
5 - PTSD-drug pre-treatment (received PGB BID 24 hours before and for a period of 10 days after three-day restraint shock);
6 - PTSD-post-treatment (received PGB injections BID for 10 days after three-day restraint/shock)

Group

		Frequency	Percent	Valid Percent	Cumulative Percent
Valid	1 control-vehicle	10	16.7	16.7	16.7
	2 L-Th control drug	10	16.7	16.7	33.3
	3 control-naïve	10	16.7	16.7	50.0
	4 PTSD-vehicle	10	16.7	16.7	66.7
	5 PTSD-drug pre-treatment	10	16.7	16.7	83.3
	6 PTSD-post-treatment	10	16.7	16.7	100.0
	Total	60	100.0	100.0	

Statistical Analysis: For this design a one-way ANOVA will be conducted for each of the outcome variables. All assumptions will be examined including homogeneity of error variances (via the Levine test) and normality. The eta-squared (η^2) effect size will be reported. Though interpreting and casting judgment as to what constitutes a small/medium/large effect size is context-dependent using Cohen's (1988) taxonomy .01/.059/138 will be small/medium/large. As well, all outliers and data anomalies will be examined and addressed accordingly (e.g., transformations, nonparametric options, etc.). In the event of a significant result ($\alpha = .05$) post hoc tests (e.g., Tukey's HSD) will be performed. Descriptive statistics and graphics will be provided for the full sample ($n = 60$) and by group.

Cohen (1988). *Statistical power analysis for the behavioral sciences*. (2nd Ed.). Hillsdale, NJ: Lawrence Erlbaum.

Descriptive Statistics: Full sample

Statistics

		Distance	Meanspeed Mean speed	Maxspeed Max speed	ZONE1entries ZONE 1 : entries	ZONE1time ZONE 1 : time	ZONE1distance ZONE 1 : distance	ZONE1latencytofirstentry ZONE 1 : latency to first entry	ZONE1maxspeed ZONE 1 : max speed
N	Valid	60	60	60	60	60	60	60	60
	Missing	0	0	0	0	0	0	0	0
Mean		14.75612	.24592	.40417	5.22	16.943	4.05363	7.200	.40927
Std. Error of Mean		.211584	.003525	.004257	.215	.7796	.160388	.8616	.006544
Median		14.87250	.24800	.40700	5.00	16.950	4.09200	5.800	.40650
Mode		16.745	.248	.428	5	10.5[a]	4.233[a]	.0[a]	.398
Std. Deviation		1.638925	.027303	.032974	1.668	6.0388	1.242361	6.6741	.050690
Variance		2.686	.001	.001	2.783	36.467	1.543	44.543	.003
Skewness		-.051	-.047	-1.022	.437	.655	.337	1.340	1.223
Std. Error of Skewness		.309	.309	.309	.309	.309	.309	.309	.309
Kurtosis		-.721	-.712	3.479	-.114	.788	.230	1.827	5.691
Std. Error of Kurtosis		.608	.608	.608	.608	.608	.608	.608	.608
Range		6.627	.111	.200	8	30.7	6.330	28.8	.354
Minimum		11.347	.189	.270	2	4.3	.999	.0	.277
Maximum		17.974	.300	.470	10	35.0	7.329	28.8	.631
Sum		885.367	14.755	24.250	313	1016.6	243.218	432.0	24.556

a. Multiple modes exist. The smallest value is shown

Distance

		Frequency	Percent	Valid Percent	Cumulative Percent
Valid	11.347	1	1.7	1.7	1.7
	11.778	1	1.7	1.7	3.3
	11.972	1	1.7	1.7	5.0
	12.092	1	1.7	1.7	6.7
	12.262	1	1.7	1.7	8.3
	12.413	1	1.7	1.7	10.0
	12.588	1	1.7	1.7	11.7
	12.752	1	1.7	1.7	13.3
	12.800	1	1.7	1.7	15.0
	12.936	1	1.7	1.7	16.7
	12.978	1	1.7	1.7	18.3
	13.102	1	1.7	1.7	20.0
	13.105	1	1.7	1.7	21.7
	13.367	1	1.7	1.7	23.3
	13.585	1	1.7	1.7	25.0
	13.667	1	1.7	1.7	26.7
	13.748	1	1.7	1.7	28.3
	13.801	1	1.7	1.7	30.0
	13.843	1	1.7	1.7	31.7
	13.970	1	1.7	1.7	33.3
	14.217	1	1.7	1.7	35.0
	14.274	1	1.7	1.7	36.7
	14.295	1	1.7	1.7	38.3
	14.330	1	1.7	1.7	40.0
	14.334	1	1.7	1.7	41.7
	14.447	1	1.7	1.7	43.3
	14.500	1	1.7	1.7	45.0
	14.542	1	1.7	1.7	46.7
	14.751	1	1.7	1.7	48.3
	14.864	1	1.7	1.7	50.0
	14.881	1	1.7	1.7	51.7
	14.902	1	1.7	1.7	53.3
	14.966	1	1.7	1.7	55.0
	15.054	1	1.7	1.7	56.7
	15.072	1	1.7	1.7	58.3
	15.142	1	1.7	1.7	60.0
	15.160	1	1.7	1.7	61.7
	15.174	1	1.7	1.7	63.3
	15.386	1	1.7	1.7	65.0
	15.488	1	1.7	1.7	66.7
	15.506	1	1.7	1.7	68.3
	15.626	1	1.7	1.7	70.0
	15.644	1	1.7	1.7	71.7
	15.665	1	1.7	1.7	73.3
	16.029	1	1.7	1.7	75.0
	16.131	1	1.7	1.7	76.7
	16.195	1	1.7	1.7	78.3
	16.308	1	1.7	1.7	80.0
	16.428	1	1.7	1.7	81.7
	16.491	1	1.7	1.7	83.3
	16.519	1	1.7	1.7	85.0
	16.745	2	3.3	3.3	88.3
	16.819	1	1.7	1.7	90.0
	16.943	1	1.7	1.7	91.7
	17.105	1	1.7	1.7	93.3
	17.162	1	1.7	1.7	95.0
	17.621	1	1.7	1.7	96.7
	17.826	1	1.7	1.7	98.3
	17.974	1	1.7	1.7	100.0
	Total	60	100.0	100.0	

Meanspeed Mean speed

		Frequency	Percent	Valid Percent	Cumulative Percent
Valid	.189	1	1.7	1.7	1.7
	.196	1	1.7	1.7	3.3
	.200	1	1.7	1.7	5.0
	.202	1	1.7	1.7	6.7
	.204	1	1.7	1.7	8.3
	.207	1	1.7	1.7	10.0
	.210	1	1.7	1.7	11.7
	.213	2	3.3	3.3	15.0
	.216	2	3.3	3.3	18.3
	.218	2	3.3	3.3	21.7
	.223	1	1.7	1.7	23.3
	.226	1	1.7	1.7	25.0
	.228	1	1.7	1.7	26.7
	.229	1	1.7	1.7	28.3
	.230	1	1.7	1.7	30.0
	.231	1	1.7	1.7	31.7
	.233	1	1.7	1.7	33.3
	.237	1	1.7	1.7	35.0
	.238	2	3.3	3.3	38.3
	.239	2	3.3	3.3	41.7
	.241	1	1.7	1.7	43.3
	.242	2	3.3	3.3	46.7
	.246	1	1.7	1.7	48.3
	.248	3	5.0	5.0	53.3
	.249	1	1.7	1.7	55.0
	.251	2	3.3	3.3	58.3
	.252	1	1.7	1.7	60.0
	.253	2	3.3	3.3	63.3
	.256	1	1.7	1.7	65.0
	.258	2	3.3	3.3	68.3
	.260	1	1.7	1.7	70.0
	.261	2	3.3	3.3	73.3
	.267	1	1.7	1.7	75.0
	.269	1	1.7	1.7	76.7
	.270	1	1.7	1.7	78.3
	.272	1	1.7	1.7	80.0
	.274	1	1.7	1.7	81.7
	.275	2	3.3	3.3	85.0
	.279	2	3.3	3.3	88.3
	.280	1	1.7	1.7	90.0
	.282	1	1.7	1.7	91.7
	.285	1	1.7	1.7	93.3
	.286	1	1.7	1.7	95.0
	.294	1	1.7	1.7	96.7
	.297	1	1.7	1.7	98.3
	.300	1	1.7	1.7	100.0
	Total	60	100.0	100.0	

Maxspeed Max speed

		Frequency	Percent	Valid Percent	Cumulative Percent
Valid	.270	1	1.7	1.7	1.7
	.339	1	1.7	1.7	3.3
	.359	2	3.3	3.3	6.7
	.366	1	1.7	1.7	8.3
	.367	1	1.7	1.7	10.0
	.369	1	1.7	1.7	11.7
	.372	2	3.3	3.3	15.0
	.373	3	5.0	5.0	20.0
	.380	1	1.7	1.7	21.7
	.385	1	1.7	1.7	23.3
	.386	1	1.7	1.7	25.0
	.387	1	1.7	1.7	26.7
	.388	1	1.7	1.7	28.3
	.389	1	1.7	1.7	30.0
	.394	3	5.0	5.0	35.0
	.395	2	3.3	3.3	38.3
	.399	1	1.7	1.7	40.0
	.400	1	1.7	1.7	41.7
	.401	3	5.0	5.0	46.7
	.402	1	1.7	1.7	48.3
	.407	3	5.0	5.0	53.3
	.408	2	3.3	3.3	56.7
	.409	1	1.7	1.7	58.3
	.414	1	1.7	1.7	60.0
	.415	2	3.3	3.3	63.3
	.418	1	1.7	1.7	65.0
	.420	1	1.7	1.7	66.7
	.421	3	5.0	5.0	71.7
	.427	1	1.7	1.7	73.3
	.428	4	6.7	6.7	80.0
	.429	1	1.7	1.7	81.7
	.434	1	1.7	1.7	83.3
	.435	3	5.0	5.0	88.3
	.441	1	1.7	1.7	90.0
	.442	2	3.3	3.3	93.3
	.455	1	1.7	1.7	95.0
	.456	1	1.7	1.7	96.7
	.463	1	1.7	1.7	98.3
	.470	1	1.7	1.7	100.0
	Total	60	100.0	100.0	

ZONE1entries ZONE 1 : entries

		Frequency	Percent	Valid Percent	Cumulative Percent
Valid	2	1	1.7	1.7	1.7
	3	9	15.0	15.0	16.7
	4	11	18.3	18.3	35.0
	5	16	26.7	26.7	61.7
	6	9	15.0	15.0	76.7
	7	8	13.3	13.3	90.0
	8	5	8.3	8.3	98.3
	10	1	1.7	1.7	100.0
	Total	60	100.0	100.0	

ZONE1time ZONE 1 : time

		Frequency	Percent	Valid Percent	Cumulative Percent
Valid	4.3	1	1.7	1.7	1.7
	7.8	1	1.7	1.7	3.3
	8.5	1	1.7	1.7	5.0
	8.6	1	1.7	1.7	6.7
	9.4	1	1.7	1.7	8.3
	9.5	1	1.7	1.7	10.0
	10.5	2	3.3	3.3	13.3
	10.6	1	1.7	1.7	15.0
	10.9	2	3.3	3.3	18.3
	11.0	1	1.7	1.7	20.0
	11.3	1	1.7	1.7	21.7
	12.4	2	3.3	3.3	25.0
	12.7	1	1.7	1.7	26.7
	12.8	1	1.7	1.7	28.3
	13.4	1	1.7	1.7	30.0
	13.9	1	1.7	1.7	31.7
	14.0	1	1.7	1.7	33.3
	14.1	1	1.7	1.7	35.0
	14.3	2	3.3	3.3	38.3
	14.5	1	1.7	1.7	40.0
	15.0	1	1.7	1.7	41.7
	15.5	1	1.7	1.7	43.3
	15.6	1	1.7	1.7	45.0
	15.8	1	1.7	1.7	46.7
	16.0	1	1.7	1.7	48.3
	16.9	1	1.7	1.7	50.0
	17.0	2	3.3	3.3	53.3
	17.2	1	1.7	1.7	55.0
	17.3	1	1.7	1.7	56.7
	17.7	2	3.3	3.3	60.0
	17.8	1	1.7	1.7	61.7
	18.1	1	1.7	1.7	63.3
	18.8	1	1.7	1.7	65.0
	19.0	1	1.7	1.7	66.7
	19.3	1	1.7	1.7	68.3
	19.8	1	1.7	1.7	70.0
	20.6	1	1.7	1.7	71.7
	20.7	1	1.7	1.7	73.3
	20.8	1	1.7	1.7	75.0
	21.2	2	3.3	3.3	78.3
	21.4	1	1.7	1.7	80.0
	21.6	1	1.7	1.7	81.7
	21.8	1	1.7	1.7	83.3
	21.9	1	1.7	1.7	85.0
	22.3	1	1.7	1.7	86.7
	22.6	1	1.7	1.7	88.3
	22.8	1	1.7	1.7	90.0
	23.3	1	1.7	1.7	91.7
	25.6	1	1.7	1.7	93.3
	27.8	1	1.7	1.7	95.0
	29.1	1	1.7	1.7	96.7
	32.8	1	1.7	1.7	98.3
	35.0	1	1.7	1.7	100.0
	Total	60	100.0	100.0	

ZONE1distance ZONE 1 : distance

		Frequency	Percent	Valid Percent	Cumulative Percent
Valid	.999	1	1.7	1.7	1.7
	2.065	1	1.7	1.7	3.3
	2.263	1	1.7	1.7	5.0
	2.401	1	1.7	1.7	6.7
	2.450	1	1.7	1.7	8.3
	2.475	1	1.7	1.7	10.0
	2.683	1	1.7	1.7	11.7
	2.690	1	1.7	1.7	13.3
	2.750	1	1.7	1.7	15.0
	2.856	1	1.7	1.7	16.7
	2.948	1	1.7	1.7	18.3
	2.980	1	1.7	1.7	20.0
	3.033	1	1.7	1.7	21.7
	3.107	1	1.7	1.7	23.3
	3.113	1	1.7	1.7	25.0
	3.160	1	1.7	1.7	26.7
	3.167	1	1.7	1.7	28.3
	3.280	1	1.7	1.7	30.0
	3.294	1	1.7	1.7	31.7
	3.315	1	1.7	1.7	33.3
	3.396	1	1.7	1.7	35.0
	3.442	1	1.7	1.7	36.7
	3.492	1	1.7	1.7	38.3
	3.612	1	1.7	1.7	40.0
	3.647	1	1.7	1.7	41.7
	3.668	1	1.7	1.7	43.3
	3.813	1	1.7	1.7	45.0
	3.827	1	1.7	1.7	46.7
	3.944	1	1.7	1.7	48.3
	3.951	1	1.7	1.7	50.0
	4.233	2	3.3	3.3	53.3
	4.258	1	1.7	1.7	55.0
	4.279	1	1.7	1.7	56.7
	4.304	1	1.7	1.7	58.3
	4.353	1	1.7	1.7	60.0
	4.427	2	3.3	3.3	63.3
	4.456	1	1.7	1.7	65.0
	4.491	1	1.7	1.7	66.7
	4.565	1	1.7	1.7	68.3
	4.625	1	1.7	1.7	70.0
	4.710	1	1.7	1.7	71.7
	4.756	1	1.7	1.7	73.3
	4.766	1	1.7	1.7	75.0
	4.773	1	1.7	1.7	76.7
	4.861	1	1.7	1.7	78.3
	4.914	1	1.7	1.7	80.0
	4.964	1	1.7	1.7	81.7
	4.978	1	1.7	1.7	83.3
	5.087	1	1.7	1.7	85.0
	5.091	1	1.7	1.7	86.7
	5.236	1	1.7	1.7	88.3
	5.681	1	1.7	1.7	90.0
	5.857	1	1.7	1.7	91.7
	6.195	1	1.7	1.7	93.3
	6.217	1	1.7	1.7	95.0
	6.612	1	1.7	1.7	96.7
	6.719	1	1.7	1.7	98.3
	7.329	1	1.7	1.7	100.0
	Total	60	100.0	100.0	

ZONE1latencytofirstentry ZONE 1 : latency to first entry

		Frequency	Percent	Valid Percent	Cumulative Percent
Valid	.0	3	5.0	5.0	5.0
	.4	1	1.7	1.7	6.7
	.7	1	1.7	1.7	8.3
	.9	1	1.7	1.7	10.0
	1.1	2	3.3	3.3	13.3
	1.2	1	1.7	1.7	15.0
	1.4	3	5.0	5.0	20.0
	1.6	1	1.7	1.7	21.7
	1.7	1	1.7	1.7	23.3
	1.8	1	1.7	1.7	25.0
	2.0	1	1.7	1.7	26.7
	2.1	1	1.7	1.7	28.3
	2.2	1	1.7	1.7	30.0
	2.3	1	1.7	1.7	31.7
	2.4	1	1.7	1.7	33.3
	2.5	1	1.7	1.7	35.0
	2.6	1	1.7	1.7	36.7
	2.8	1	1.7	1.7	38.3
	3.0	1	1.7	1.7	40.0
	3.2	1	1.7	1.7	41.7
	3.9	1	1.7	1.7	43.3
	4.6	1	1.7	1.7	45.0
	5.1	1	1.7	1.7	46.7
	5.3	1	1.7	1.7	48.3
	5.6	1	1.7	1.7	50.0
	6.0	1	1.7	1.7	51.7
	6.2	1	1.7	1.7	53.3
	6.3	1	1.7	1.7	55.0
	6.5	1	1.7	1.7	56.7
	6.7	1	1.7	1.7	58.3
	6.9	1	1.7	1.7	60.0
	7.5	1	1.7	1.7	61.7
	7.7	1	1.7	1.7	63.3
	8.2	1	1.7	1.7	65.0
	8.6	1	1.7	1.7	66.7
	9.4	1	1.7	1.7	68.3
	9.8	1	1.7	1.7	70.0
	10.0	1	1.7	1.7	71.7
	10.1	1	1.7	1.7	73.3
	10.3	1	1.7	1.7	75.0
	10.4	1	1.7	1.7	76.7
	10.6	1	1.7	1.7	78.3
	11.3	2	3.3	3.3	81.7
	13.5	1	1.7	1.7	83.3
	14.2	1	1.7	1.7	85.0
	14.8	1	1.7	1.7	86.7
	15.7	1	1.7	1.7	88.3
	16.0	1	1.7	1.7	90.0
	16.7	1	1.7	1.7	91.7
	18.4	1	1.7	1.7	93.3
	18.5	1	1.7	1.7	95.0
	18.9	1	1.7	1.7	96.7
	28.4	1	1.7	1.7	98.3
	28.8	1	1.7	1.7	100.0
	Total	60	100.0	100.0	

ZONE1maxspeed ZONE 1 : max speed

		Frequency	Percent	Valid Percent	Cumulative Percent
Valid	.277	1	1.7	1.7	1.7
	.329	1	1.7	1.7	3.3
	.332	1	1.7	1.7	5.0
	.346	1	1.7	1.7	6.7
	.348	2	3.3	3.3	10.0
	.360	2	3.3	3.3	13.3
	.362	1	1.7	1.7	15.0
	.363	3	5.0	5.0	20.0
	.381	3	5.0	5.0	25.0
	.385	1	1.7	1.7	26.7
	.394	1	1.7	1.7	28.3
	.396	3	5.0	5.0	33.3
	.398	9	15.0	15.0	48.3
	.400	1	1.7	1.7	50.0
	.413	6	10.0	10.0	60.0
	.415	4	6.7	6.7	66.7
	.417	1	1.7	1.7	68.3
	.431	5	8.3	8.3	76.7
	.433	2	3.3	3.3	80.0
	.435	1	1.7	1.7	81.7
	.450	2	3.3	3.3	85.0
	.451	2	3.3	3.3	88.3
	.452	2	3.3	3.3	91.7
	.480	1	1.7	1.7	93.3
	.485	2	3.3	3.3	96.7
	.515	1	1.7	1.7	98.3
	.631	1	1.7	1.7	100.0
	Total	60	100.0	100.0	

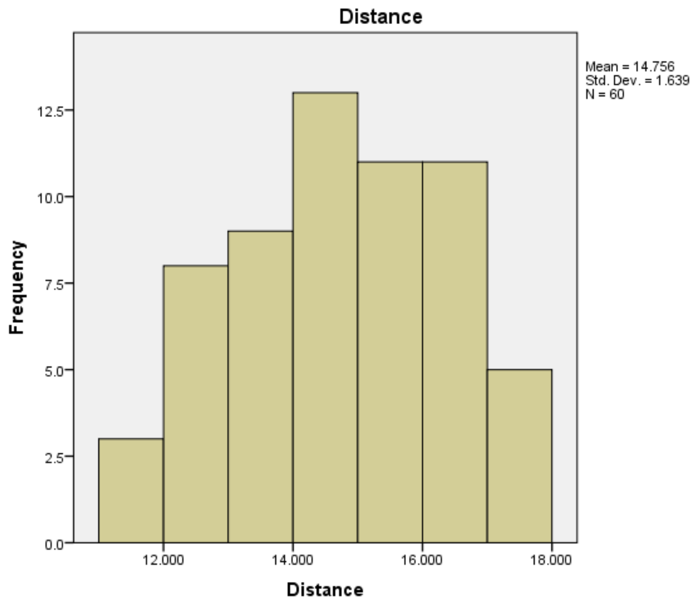

Distance
Mean = 14.756
Std. Dev. = 1.639
N = 60
12.5
10.0
7.5
5.0
2.5
0.0
Frequency
12.000
14.000
16.000
18.000
Distance

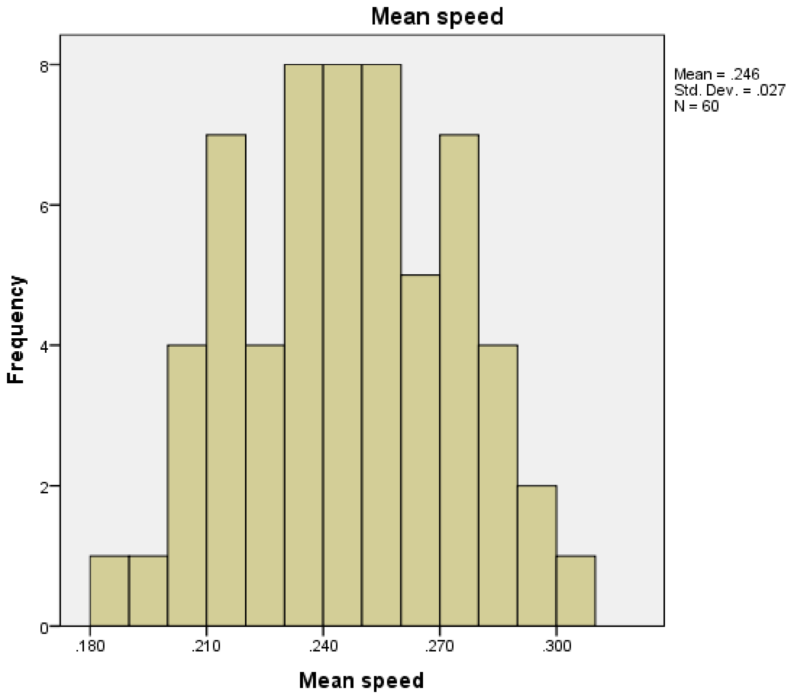

Mean speed
Mean = .246
Std. Dev. = .027
N = 60
8
6
4
2
0
Frequency
.180
.210
.240
.270
.300
Mean speed

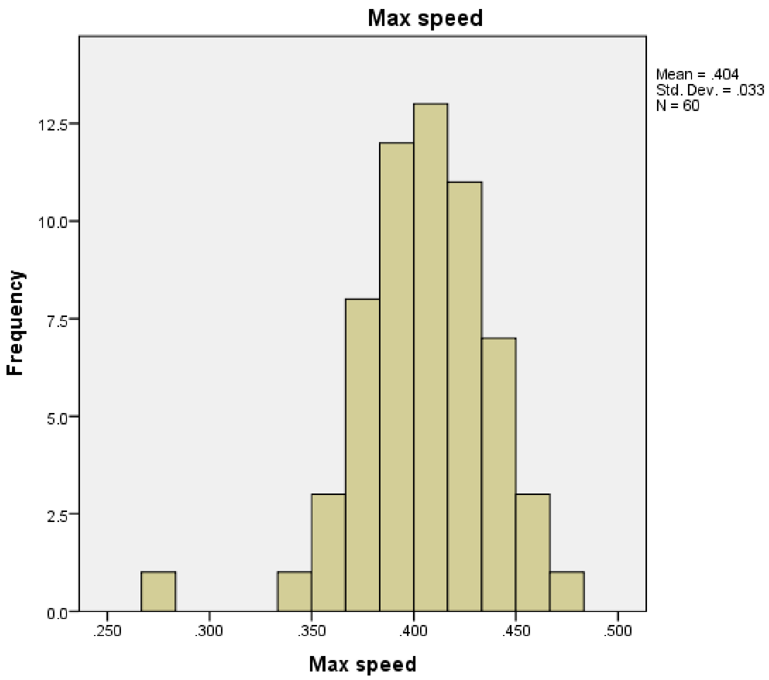
Max speed
Mean = .404
Std. Dev. = .033
N = 60
Frequency
0.0
2.5
5.0
7.5
10.0
12.5
.250
.300
.350
.400
.450
.500
Max speed

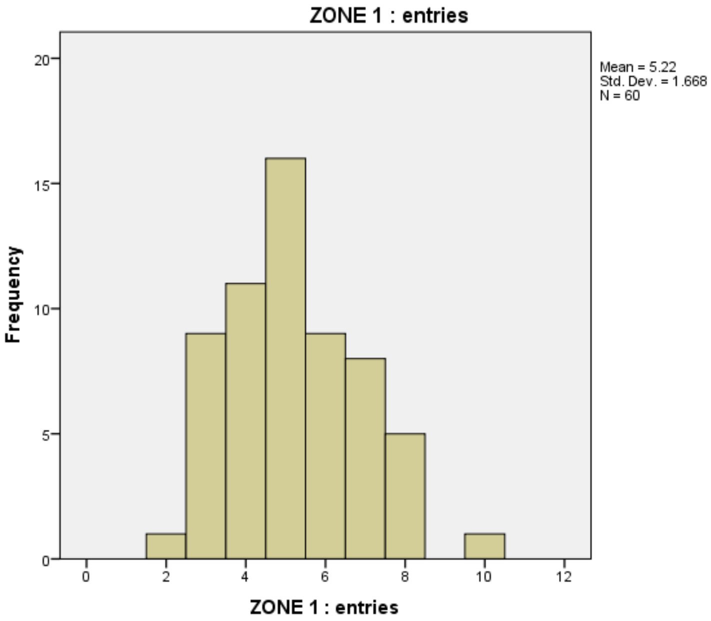
ZONE 1 : entries
Mean = 5.22
Std. Dev. = 1.668
N = 60
Frequency
0
5
10
15
20
0
2
4
6
8
10
12
ZONE 1 : entries

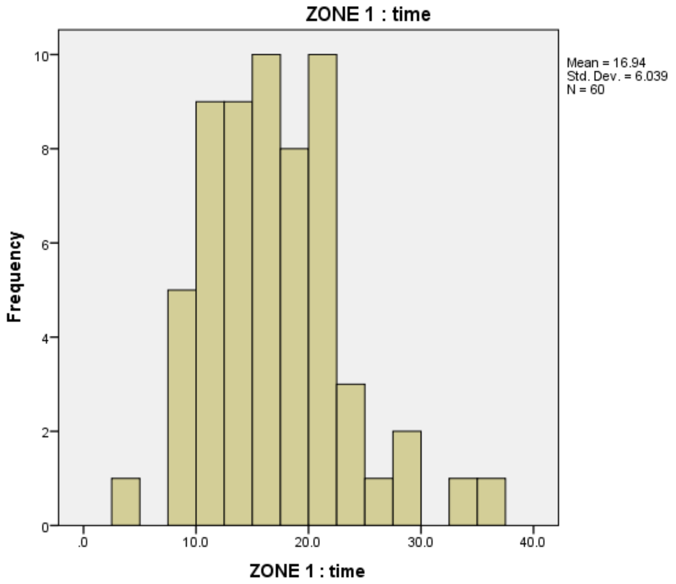
ZONE 1 : time
Mean = 16.94
Std. Dev. = 6.039
N = 60
Frequency
0
2
4
6
8
10
.0
10.0
20.0
30.0
40.0
ZONE 1 : time

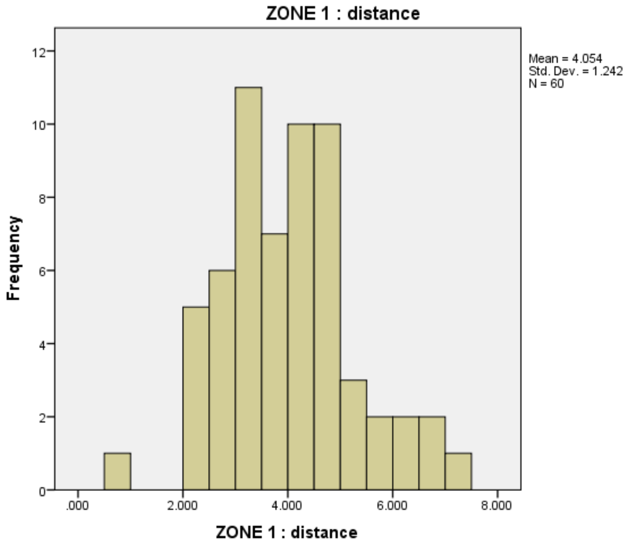
ZONE 1 : distance
Mean = 4.054
Std. Dev. = 1.242
N = 60
Frequency
0
2
4
6
8
10
12
.000
2.000
4.000
6.000
8.000
ZONE 1 : distance

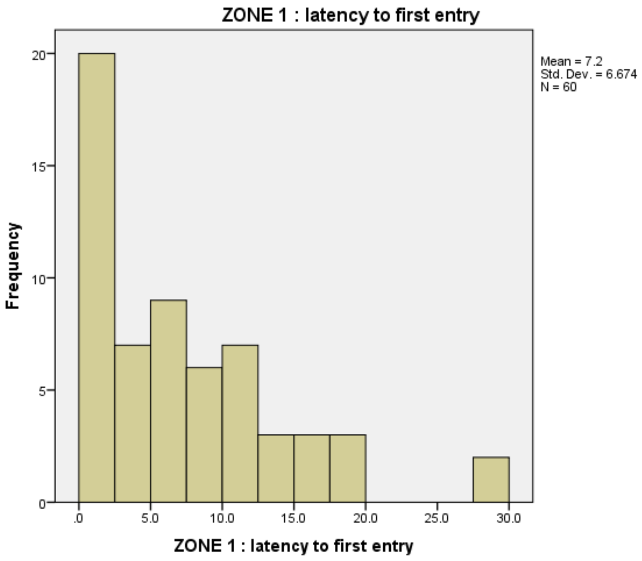
ZONE 1 : latency to first entry
Mean = 7.2
Std. Dev. = 6.674
N = 60
Frequency
20
15
10
5
0
.0
5.0
10.0
15.0
20.0
25.0
30.0
ZONE 1 : latency to first entry

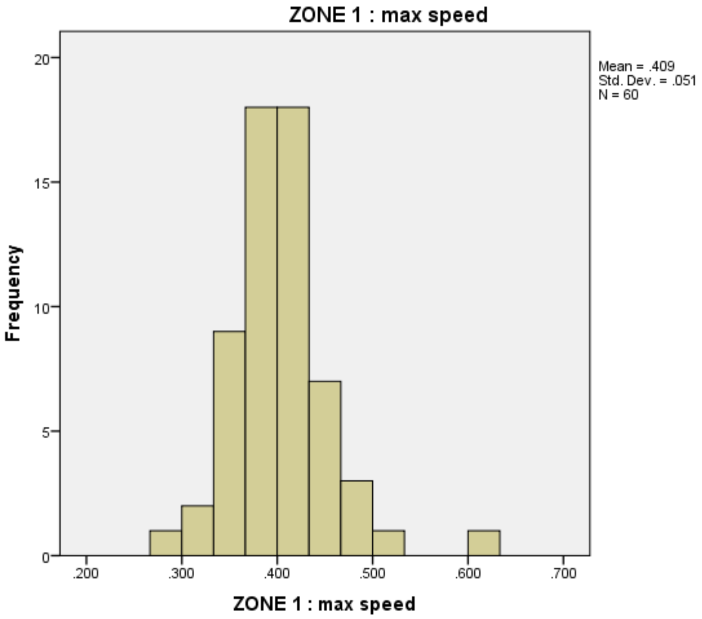
ZONE 1 : max speed
Mean = .409
Std. Dev. = .051
N = 60
Frequency
20
15
10
5
0
.200
.300
.400
.500
.600
.700
ZONE 1 : max speed

By Group

Statistics[a]

		Distance	Meanspeed Mean speed	Maxspeed Max speed	ZONE1entries ZONE 1 : entries	ZONE1time ZONE 1 : time	ZONE1distance ZONE 1 : distance	ZONE1latencytofirstentry ZONE 1 : latency to first entry	ZONE1maxspeed ZONE 1 : max speed
N	Valid	10	10	10	10	10	10	10	10
	Missing	0	0	0	0	0	0	0	0
Mean		14.55410	.24260	.41310	5.00	14.460	3.51650	9.760	.41340
Std. Error of Mean		.477502	.007902	.008703	.745	1.8507	.413659	2.6371	.011006
Median		14.41700	.24050	.40850	4.50	14.100	3.10000	7.950	.40550
Mode		12.092[b]	.239	.372[b]	3[b]	7.8[b]	2.065[b]	1.1[b]	.398
Std. Deviation		1.509994	.024990	.027522	2.357	5.8523	1.308105	8.3394	.034805
Variance		2.280	.001	.001	5.556	34.249	1.711	69.545	.001
Skewness		-.213	-.208	.706	.955	1.260	1.771	1.291	.850
Std. Error of Skewness		.687	.687	.687	.687	.687	.687	.687	.687
Kurtosis		-.198	-.189	1.043	1.021	2.344	3.985	1.780	.938
Std. Error of Kurtosis		1.334	1.334	1.334	1.334	1.334	1.334	1.334	1.334
Range		4.727	.078	.098	8	20.0	4.654	27.3	.122
Minimum		12.092	.202	.372	2	7.8	2.065	1.1	.363
Maximum		16.819	.280	.470	10	27.8	6.719	28.4	.485
Sum		145.541	2.426	4.131	50	144.6	35.165	97.6	4.134

a. Group = 1 control-vehicle

b. Multiple modes exist. The smallest value is shown

Statistics[a]

		Distance	Meanspeed Mean speed	Maxspeed Max speed	ZONE1entries ZONE 1 : entries	ZONE1time ZONE 1 : time	ZONE1distance ZONE 1 : distance	ZONE1latencytofirstentry ZONE 1 : latency to first entry	ZONE1maxspeed ZONE 1 : max speed
N	Valid	10	10	10	10	10	10	10	10
	Missing	0	0	0	0	0	0	0	0
Mean		14.87760	.24790	.41430	4.70	15.200	3.69440	10.670	.40460
Std. Error of Mean		.480676	.007984	.006838	.423	1.5101	.383844	2.6110	.015248
Median		14.97650	.24950	.41250	4.50	15.750	3.55200	10.050	.41300
Mode		12.936[b]	.216[b]	.407	4	8.6[b]	2.263[b]	.0[b]	.413
Std. Deviation		1.520030	.025247	.021623	1.337	4.7754	1.213820	8.2569	.048220
Variance		2.310	.001	.000	1.789	22.804	1.473	68.176	.002
Skewness		.611	.615	-.072	.334	-.080	.124	1.096	-.096
Std. Error of Skewness		.687	.687	.687	.687	.687	.687	.687	.687
Kurtosis		.121	.145	-1.538	-.852	-1.627	-1.985	1.917	-.491
Std. Error of Kurtosis		1.334	1.334	1.334	1.334	1.334	1.334	1.334	1.334
Range		4.890	.081	.057	4	13.0	2.973	28.8	.156
Minimum		12.936	.216	.385	3	8.6	2.263	.0	.329
Maximum		17.826	.297	.442	7	21.6	5.236	28.8	.485
Sum		148.776	2.479	4.143	47	152.0	36.944	106.7	4.046

a. Group = 2 L-Th control drug

b. Multiple modes exist. The smallest value is shown

Statistics[a]

		Distance	Meanspeed Mean speed	Maxspeed Max speed	ZONE1entries ZONE 1 : entries	ZONE1time ZONE 1 : time	ZONE1distance ZONE 1 : distance	ZONE1latencytofirstentry ZONE 1 : latency to first entry	ZONE1maxspeed ZONE 1 : max speed
N	Valid	10	10	10	10	10	10	10	10
	Missing	0	0	0	0	0	0	0	0
Mean		15.04290	.25060	.41240	5.30	19.370	4.53590	4.540	.40400
Std. Error of Mean		.419332	.007030	.011905	.559	1.5300	.363886	2.0355	.012903
Median		15.05400	.25050	.41350	5.50	18.800	4.39000	1.400	.39600
Mode		13.102[b]	.218[b]	.369[b]	3[b]	12.4[b]	3.294[b]	1.4	.381
Std. Deviation		1.326043	.022232	.037648	1.767	4.8383	1.150709	6.4369	.040803
Variance		1.758	.000	.001	3.122	23.409	1.324	41.434	.002
Skewness		.461	.482	.135	.036	.602	1.632	1.689	.603
Std. Error of Skewness		.687	.687	.687	.687	.687	.687	.687	.687
Kurtosis		.272	.314	-1.812	-1.384	.545	3.827	1.614	-.188
Std. Error of Kurtosis		1.334	1.334	1.334	1.334	1.334	1.334	1.334	1.334
Range		4.519	.076	.094	5	16.7	4.035	18.4	.132
Minimum		13.102	.218	.369	3	12.4	3.294	.0	.348
Maximum		17.621	.294	.463	8	29.1	7.329	18.4	.480
Sum		150.429	2.506	4.124	53	193.7	45.359	45.4	4.040

a. Group = 3 control-naïve

b. Multiple modes exist. The smallest value is shown

Statistics[a]

		Distance	Meanspeed Mean speed	Maxspeed Max speed	ZONE1entries ZONE 1 : entries	ZONE1time ZONE 1 : time	ZONE1distance ZONE 1 : distance	ZONE1latencytofirstentry ZONE 1 : latency to first entry	ZONE1maxspeed ZONE 1 : max speed
N	Valid	10	10	10	10	10	10	10	10
	Missing	0	0	0	0	0	0	0	0
Mean		15.28050	.25470	.39990	5.30	17.090	4.16040	5.140	.39360
Std. Error of Mean		.479612	.007940	.010308	.396	1.7413	.299289	1.5988	.009057
Median		15.54150	.25900	.40750	5.00	16.300	4.37450	3.500	.39800
Mode		16.745	.279	.401[b]	5	10.5[b]	2.683[b]	1.6[b]	.398
Std. Deviation		1.516665	.025109	.032597	1.252	5.5065	.946436	5.0557	.028640
Variance		2.300	.001	.001	1.567	30.321	.896	25.560	.001
Skewness		-.474	-.470	-.858	-.280	.214	-.227	2.400	-.291
Std. Error of Skewness		.687	.687	.687	.687	.687	.687	.687	.687
Kurtosis		-1.262	-1.286	-.458	-.066	-1.508	-.695	6.469	-.923
Std. Error of Kurtosis		1.334	1.334	1.334	1.334	1.334	1.334	1.334	1.334
Range		4.191	.069	.096	4	15.1	2.998	16.9	.083
Minimum		12.752	.213	.339	3	10.5	2.683	1.6	.348
Maximum		16.943	.282	.435	7	25.6	5.681	18.5	.431
Sum		152.805	2.547	3.999	53	170.9	41.604	51.4	3.936

a. Group = 4 PTSD-vehicle

b. Multiple modes exist. The smallest value is shown

Statistics[a]

		Distance	Meanspeed Mean speed	Maxspeed Max speed	ZONE1entries ZONE 1 : entries	ZONE1time ZONE 1 : time	ZONE1distance ZONE 1 : distance	ZONE1latencytofirstentry ZONE 1 : latency to first entry	ZONE1maxspeed ZONE 1 : max speed
N	Valid	10	10	10	10	10	10	10	10
	Missing	0	0	0	0	0	0	0	0
Mean		13.62540	.22700	.39510	5.10	19.180	4.23490	7.090	.42180
Std. Error of Mean		.572799	.009581	.006049	.526	2.7523	.424618	1.8641	.015319
Median		13.19250	.21950	.39400	5.00	17.000	3.83750	4.500	.42300
Mode		11.347[b]	.189[b]	.394[b]	5	10.5[b]	2.750[b]	11.3	.451
Std. Deviation		1.811349	.030299	.019128	1.663	8.7035	1.342761	5.8949	.048442
Variance		3.281	.001	.000	2.767	75.751	1.803	34.750	.002
Skewness		.627	.621	.680	1.072	1.000	.814	.555	.063
Std. Error of Skewness		.687	.687	.687	.687	.687	.687	.687	.687
Kurtosis		-.285	-.323	1.295	.374	-.130	-.588	-1.334	1.415
Std. Error of Kurtosis		1.334	1.334	1.334	1.334	1.334	1.334	1.334	1.334
Range		5.758	.096	.068	5	24.5	3.862	16.7	.183
Minimum		11.347	.189	.367	3	10.5	2.750	.0	.332
Maximum		17.105	.285	.435	8	35.0	6.612	16.7	.515
Sum		136.254	2.270	3.951	51	191.8	42.349	70.9	4.218

a. Group = 5 PTSD-drug pre-treatment

b. Multiple modes exist. The smallest value is shown

Statistics[a]

		Distance	Meanspeed Mean speed	Maxspeed Max speed	ZONE1entries ZONE 1 : entries	ZONE1time ZONE 1 : time	ZONE1distance ZONE 1 : distance	ZONE1latencytofirstentry ZONE 1 : latency to first entry	ZONE1maxspeed ZONE 1 : max speed
N	Valid	10	10	10	10	10	10	10	10
	Missing	0	0	0	0	0	0	0	0
Mean		15.15620	.25270	.39020	5.90	16.360	4.17970	6.000	.41820
Std. Error of Mean		.601780	.010034	.015654	.504	1.7261	.460614	1.3301	.028047
Median		15.57650	.25950	.40400	5.50	17.350	4.33000	6.450	.40750
Mode		11.972[b]	.200[b]	.270[b]	5	4.3[b]	.999[b]	.7[b]	.398[b]
Std. Deviation		1.902997	.031732	.049502	1.595	5.4584	1.456588	4.2061	.088693
Variance		3.621	.001	.002	2.544	29.794	2.122	17.691	.008
Skewness		-.276	-.260	-1.764	-.209	-1.244	-.898	.327	1.328
Std. Error of Skewness		.687	.687	.687	.687	.687	.687	.687	.687
Kurtosis		-.795	-.807	3.675	-.457	1.713	1.919	-.687	4.334
Std. Error of Kurtosis		1.334	1.334	1.334	1.334	1.334	1.334	1.334	1.334
Range		6.002	.100	.172	5	18.5	5.218	12.8	.354
Minimum		11.972	.200	.270	3	4.3	.999	.7	.277
Maximum		17.974	.300	.442	8	22.8	6.217	13.5	.631
Sum		151.562	2.527	3.902	59	163.6	41.797	60.0	4.182

a. Group = 6 PTSD-post-treatment

b. Multiple modes exist. The smallest value is shown

One-Way ANOVA

Distance

Descriptive Statistics

Dependent Variable: Distance

Group	Mean	Std. Deviation	N
1 control-vehicle	14.55410	1.509994	10
2 L-Th control drug	14.87760	1.520030	10
3 control-naïve	15.04290	1.326043	10
4 PTSD-vehicle	15.28050	1.516665	10
5 PTSD-drug pre-treatment	13.62540	1.811349	10
6 PTSD-post-treatment	15.15620	1.902997	10
Total	14.75612	1.638925	60

Levene's Test of Equality of Error Variances[a]

Dependent Variable: Distance

F	df1	df2	Sig.
.644	5	54	.667

Tests the null hypothesis that the error variance of the dependent variable is equal across groups.

a. Design: Intercept + Group

Tests of Between-Subjects Effects

Dependent Variable: Distance

Source	Type III Sum of Squares	df	Mean Square	F	Sig.	Partial Eta Squared
Corrected Model	18.514[a]	5	3.703	1.429	.229	.117
Intercept	13064.579	1	13064.579	5040.470	.000	.989
Group	18.514	5	3.703	1.429	.229	.117
Error	139.965	54	2.592			
Total	13223.057	60				
Corrected Total	158.478	59				

a. R Squared = .117 (Adjusted R Squared = .035)

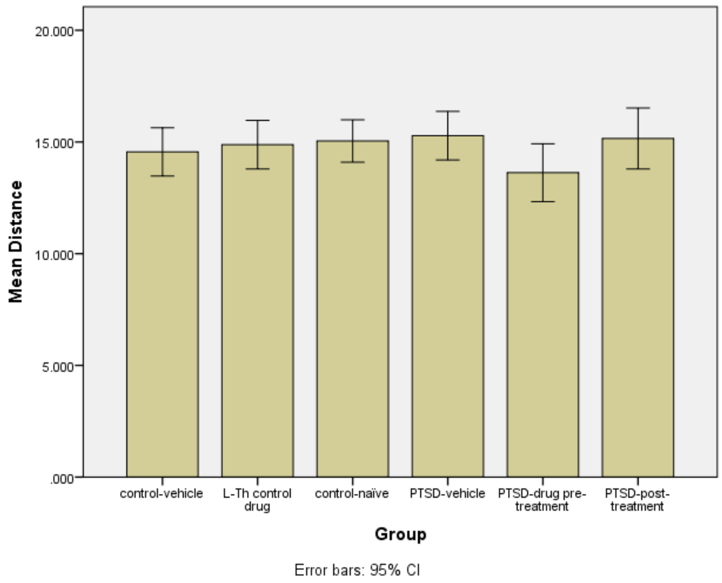

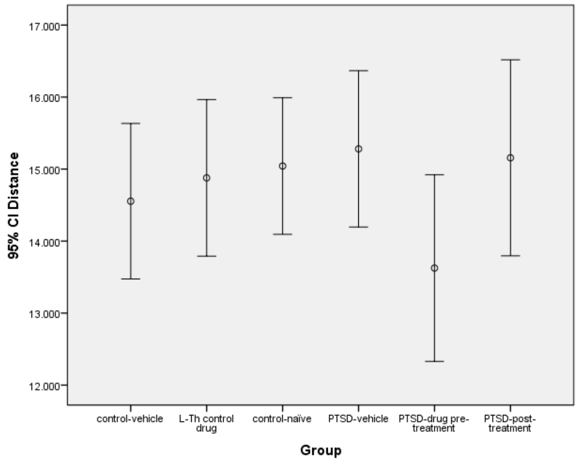

Summary: For the **distance** outcome, there was not a significant difference between the six groups: $F(5, 54) = 1.43$, $p = .229$ ($\eta^2 = .117$). Though not significant the PTSD-vehicle group has the highest mean ($M = 15.28$) and PTSD-drug pre-treatment the lowest ($M = 16.63$).

Mean Speed

Descriptive Statistics

Dependent Variable: Meanspeed Mean speed

Group	Mean	Std. Deviation	N
1 control-vehicle	.24260	.024990	10
2 L-Th control drug	.24790	.025247	10
3 control-naïve	.25060	.022232	10
4 PTSD-vehicle	.25470	.025109	10
5 PTSD-drug pre-treatment	.22700	.030299	10
6 PTSD-post-treatment	.25270	.031732	10
Total	.24592	.027303	60

Levene's Test of Equality of Error Variances[a]

Dependent Variable: Meanspeed Mean speed

F	df1	df2	Sig.
.662	5	54	.654

Tests the null hypothesis that the error variance of the dependent variable is equal across groups.

a. Design: Intercept + Group

Tests of Between-Subjects Effects

Dependent Variable: Meanspeed Mean speed

Source	Type III Sum of Squares	df	Mean Square	F	Sig.	Partial Eta Squared
Corrected Model	.005[a]	5	.001	1.441	.224	.118
Intercept	3.629	1	3.629	5049.467	.000	.989
Group	.005	5	.001	1.441	.224	.118
Error	.039	54	.001			
Total	3.672	60				
Corrected Total	.044	59				

a. R Squared = .118 (Adjusted R Squared = .036)

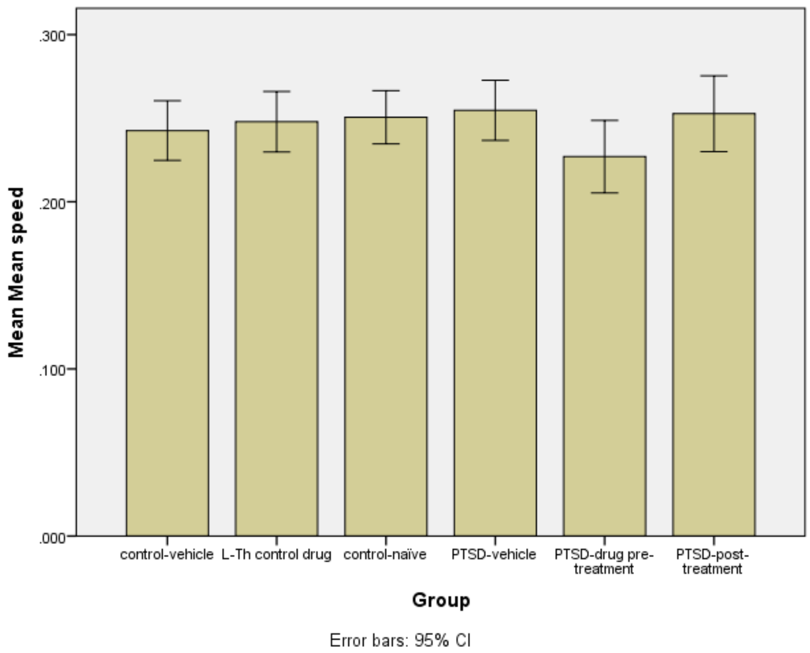

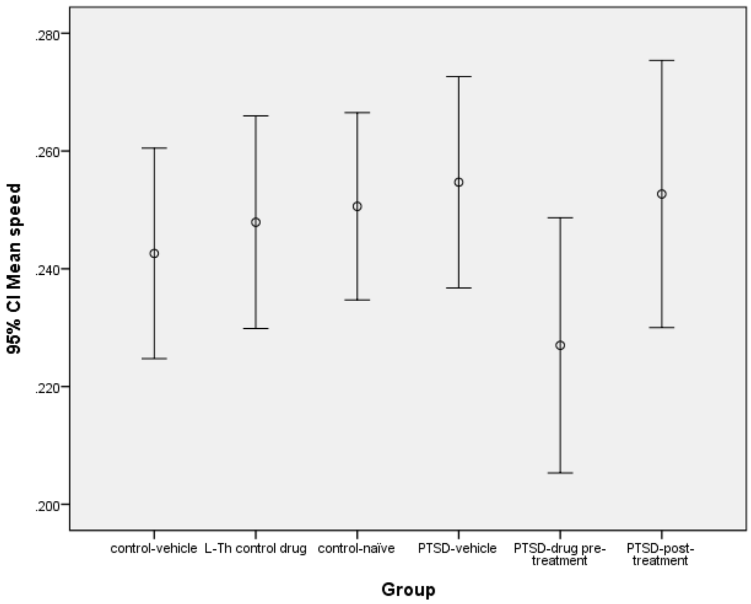

Summary: For the **Mean Speed** outcome, there was not a significant difference between the six groups: $F(5, 54) = 1.44$, $p = .224$ ($\eta^2 = .118$). Though not significant the PTSD-vehicle group has the highest mean ($M = .2547$) and PTSD-drug pre-treatment the lowest ($M = .227$).

Max Speed

Descriptive Statistics

Dependent Variable: Maxspeed Max speed

Group	Mean	Std. Deviation	N
1 control-vehicle	.41310	.027522	10
2 L-Th control drug	.41430	.021623	10
3 control-naïve	.41240	.037648	10
4 PTSD-vehicle	.39990	.032597	10
5 PTSD-drug pre-treatment	.39510	.019128	10
6 PTSD-post-treatment	.39020	.049502	10
Total	.40417	.032974	60

Levene's Test of Equality of Error Variances[a]

Dependent Variable: Maxspeed Max speed

F	df1	df2	Sig.
1.917	5	54	.107

Tests the null hypothesis that the error variance of the dependent variable is equal across groups.

a. Design: Intercept + Group

Tests of Between-Subjects Effects

Dependent Variable: Maxspeed Max speed

Source	Type III Sum of Squares	df	Mean Square	F	Sig.	Partial Eta Squared
Corrected Model	.005[a]	5	.001	1.004	.424	.085
Intercept	9.801	1	9.801	9017.704	.000	.994
Group	.005	5	.001	1.004	.424	.085
Error	.059	54	.001			
Total	9.865	60				
Corrected Total	.064	59				

a. R Squared = .085 (Adjusted R Squared = .000)

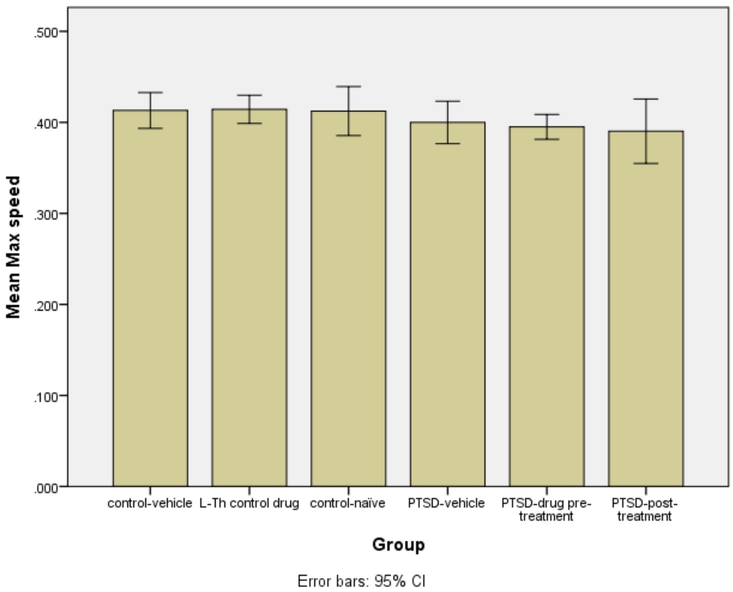

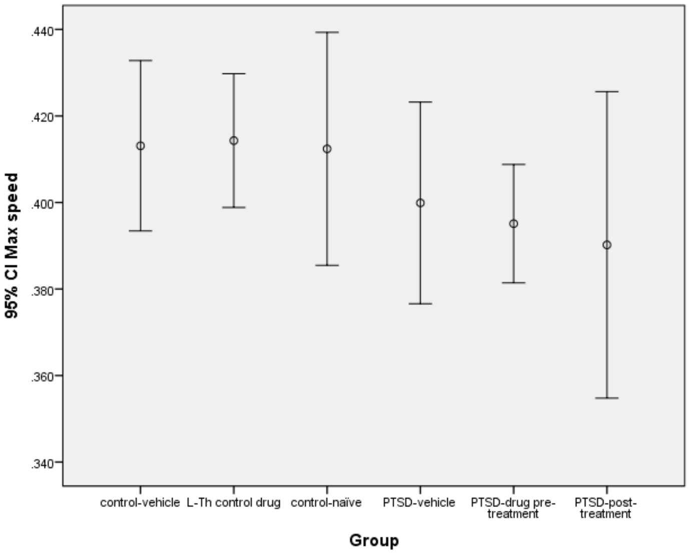

Summary: For the **Max Speed** outcome, there was not a significant difference between the six groups: $F(5, 54) = 1.00$, $p = .424$ ($\eta^2 = .085$). Though not significant the L-Th control group has the highest mean ($M = .414$) and PTSD-post-treatment the lowest ($M = .39$).

Zone 1 time

Descriptive Statistics

Dependent Variable: ZONE1time ZONE 1 : time

Group	Mean	Std. Deviation	N
1 control-vehicle	14.460	5.8523	10
2 L-Th control drug	15.200	4.7754	10
3 control-naïve	19.370	4.8383	10
4 PTSD-vehicle	17.090	5.5065	10
5 PTSD-drug pre-treatment	19.180	8.7035	10
6 PTSD-post-treatment	16.360	5.4584	10
Total	16.943	6.0388	60

Levene's Test of Equality of Error Variances[a]

Dependent Variable: ZONE1time ZONE 1 : time

F	df1	df2	Sig.
1.015	5	54	.418

Tests the null hypothesis that the error variance of the dependent variable is equal across groups.

a. Design: Intercept + Group

Tests of Between-Subjects Effects

Dependent Variable: ZONE1time ZONE 1 : time

Source	Type III Sum of Squares	df	Mean Square	F	Sig.	Partial Eta Squared
Corrected Model	204.593[a]	5	40.919	1.135	.353	.095
Intercept	17224.593	1	17224.593	477.735	.000	.898
Group	204.593	5	40.919	1.135	.353	.095
Error	1946.954	54	36.055			
Total	19376.140	60				
Corrected Total	2151.547	59				

a. R Squared = .095 (Adjusted R Squared = .011)

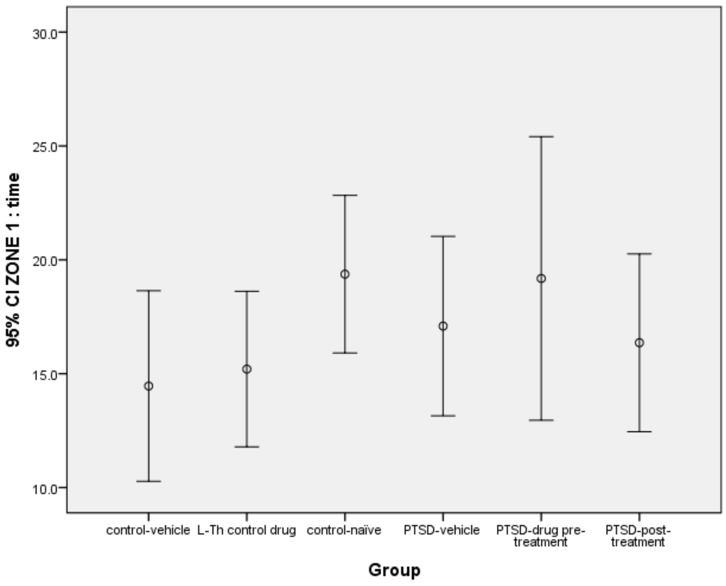

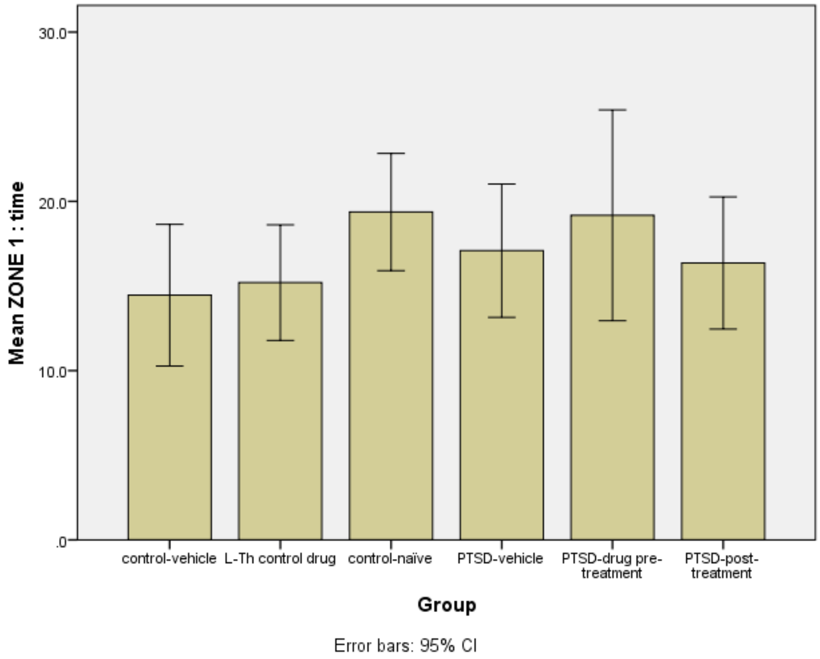

Summary: For the **Zone 1 Time** outcome, there was not a significant difference between the six groups: $F(5, 54) = 1.14$, $p = .353$ ($\eta^2 = .095$). Though not significant the control naïve group has the highest mean ($M = 19.37$) and the control vehicle group the lowest ($M = 14.46$).

Report

ZONE1time

Group	Mean	N	Std. Deviation	Std. Error of Mean
1	14.46	10	5.852	1.851
2	15.20	10	4.775	1.510
3	19.37	10	4.838	1.530
4	17.09	10	5.506	1.741
5	19.18	10	8.703	2.752
6	16.36	10	5.458	1.726
Total	16.94	60	6.039	.780
