# Supplementary material for: Effects of pregabalin on neurobehavior in an adult male rat model of PTSD
Source: PLoS One. 2018 Dec 31;13(12):e0209494. doi: 10.1371/journal.pone.0209494 (PMC6312257; doi:10.1371/journal.pone.0209494)
Supplement: S6 Fig — Each of the main two groups (Stressed and Non-stressed) had three subgroups: non-stressed: control vehicle, control PGB, control naïve; and stressed: Posttraumatic stress disorder (PTSD) vehicle, PTSD Pre-PGB (prophylactic), PTSD Post-PGB (non-prophylactic). There was no significant difference between the six groups. (PDF) [file pone.0209494.s006.pdf]

Forced Swim Test (FST)

Study: Pregabalin & L-Theanine Prophylactic Effects on PTSD Behavior and Gene Expression in Male Sprague-Dawley Rats

Per the protocol the aims of this study are as follows (amended 10/13/17):

SPECIFIC AIMS

The aims of these studies are to determine if preemptive administration of PGB or L-Th prevent PTSD development in the rodent model. Specifically, the aims are as follows:

1. Determine the effects of PGB and L-Th on anxiety
2. Determine the effects of PGB and L-Th on locomotion
3. Determine the effects of PGB and L-Th on memory
4. Determine the effects of PGB and L-Th on depression
5. Determine the effects of PGB and L-Th on gene expression in the brain (p. 14).

And:

The aims of this research protocol will be guided by the following questions:

1. Is there a significant difference in the anxiolytic effects between the groups?
2. Is there a significant difference in locomotion between the groups?
3. Is there a significant difference in memory between the groups?
4. Is there a significant difference in depression between the groups?
5. Are there significant differences in gene expression and regulation in the hippocampus between the groups?
6. Are there significant differences in gene expression and regulation in the amygdala b etween the groups? (p. 15).

The grouping variable is as follows:

There was a total of 6 groups (1-6), each with 10 rat subjects.

The three groups of non-stressed rats:
1 - control-vehicle (received vehicle injections BID);
2 - L-Th control drug (received PGB BID)*
3 - control-naïve (received no injections)

The three groups in the 3-day restraint/shock stressed rats:
4 - PTSD-vehicle (received vehicle injection BID after three-day restraint/shock);
5 - PTSD-drug pre-treatment (received PGB BID 24 hours before and for a period of 10 days after three-day restraint shock);
6 - PTSD-post-treatment (received PGB injections BID for 10 days after three-day restraint/shock)

Group

		Frequency	Percent	Valid Percent	Cumulative Percent
Valid	1 control-vehicle	10	16.7	16.7	16.7
	2 L-Th control drug	10	16.7	16.7	33.3
	3 control-naïve	10	16.7	16.7	50.0
	4 PTSD-vehicle	10	16.7	16.7	66.7
	5 PTSD-drug pre-treatment	10	16.7	16.7	83.3
	6 PTSD-post-treatment	10	16.7	16.7	100.0
	Total	60	100.0	100.0	

Statistical Analysis: For this design a one-way ANOVA will be conducted for each of the outcome variables. All assumptions will be examined including homogeneity of error variances (via the Levine test) and normality. The eta-squared (η^2) effect size will be reported. Though interpreting and casting judgment as to what constitutes a small/medium/large effect size is context-dependent using Cohen's (1988) taxonomy .01/.059/138 will be small/medium/large. As well, all outliers and data anomalies will be examined and addressed accordingly (e.g., transformations, nonparametric options, etc.). In the event of a significant result ($\alpha = .05$) post hoc tests (e.g., Tukey's HSD) will be performed. Descriptive statistics and graphics will be provided for the full sample ($n = 60$) and by group.

Cohen (1988). *Statistical power analysis for the behavioral sciences*. (2nd Ed.). Hillsdale, NJ: Lawrence Erlbaum.

Descriptive Statistics: Full sample

Statistics

		MeanTimeMobileSeconds Mean Time Mobile (Seconds)	FSTNumberFSTFecalPelletsPoop FST Number FST Fecal Pellets (Poop)
N	Valid	59	60
	Missing	1	0
Mean		47.0282	5.10
Std. Error of Mean		3.86496	.288
Median		38.3333	5.00
Mode		17.00[a]	7
Std. Deviation		29.68734	2.230
Variance		881.338	4.973
Skewness		1.081	-.376
Std. Error of Skewness		.311	.309
Kurtosis		.585	-.528
Std. Error of Kurtosis		.613	.608
Range		125.00	9
Minimum		6.33	0
Maximum		131.33	9
Sum		2774.67	306

a. Multiple modes exist. The smallest value is shown

MeanTimeMobileSeconds Mean Time Mobile (Seconds)

		Frequency	Percent	Valid Percent	Cumulative Percent
Valid	6.33	1	1.7	1.7	1.7
	11.00	1	1.7	1.7	3.4
	15.67	1	1.7	1.7	5.1
	16.33	1	1.7	1.7	6.8
	16.67	1	1.7	1.7	8.5
	17.00	2	3.3	3.4	11.9
	17.33	2	3.3	3.4	15.3
	19.00	1	1.7	1.7	16.9
	21.67	1	1.7	1.7	18.6
	22.00	1	1.7	1.7	20.3
	23.00	1	1.7	1.7	22.0
	24.00	1	1.7	1.7	23.7
	24.33	1	1.7	1.7	25.4
	24.67	2	3.3	3.4	28.8
	26.33	2	3.3	3.4	32.2
	27.33	1	1.7	1.7	33.9
	28.00	1	1.7	1.7	35.6
	29.00	1	1.7	1.7	37.3
	30.00	1	1.7	1.7	39.0
	31.33	1	1.7	1.7	40.7
	32.33	1	1.7	1.7	42.4
	35.33	1	1.7	1.7	44.1
	35.67	2	3.3	3.4	47.5
	37.67	1	1.7	1.7	49.2
	38.33	1	1.7	1.7	50.8
	43.33	1	1.7	1.7	52.5
	43.67	1	1.7	1.7	54.2
	44.67	1	1.7	1.7	55.9
	48.67	1	1.7	1.7	57.6
	50.67	1	1.7	1.7	59.3
	51.33	1	1.7	1.7	61.0
	51.67	2	3.3	3.4	64.4
	52.33	1	1.7	1.7	66.1
	53.33	1	1.7	1.7	67.8
	55.00	2	3.3	3.4	71.2
	56.67	1	1.7	1.7	72.9
	58.67	1	1.7	1.7	74.6
	59.00	2	3.3	3.4	78.0
	61.33	1	1.7	1.7	79.7
	68.00	1	1.7	1.7	81.4
	71.00	1	1.7	1.7	83.1
	79.33	1	1.7	1.7	84.7
	80.33	1	1.7	1.7	86.4
	86.67	1	1.7	1.7	88.1
	87.67	1	1.7	1.7	89.8
	93.67	1	1.7	1.7	91.5
	106.33	1	1.7	1.7	93.2
	110.67	1	1.7	1.7	94.9
	114.33	1	1.7	1.7	96.6
	118.00	1	1.7	1.7	98.3
	131.33	1	1.7	1.7	100.0
	Total	59	98.3	100.0	
Missing	System	1	1.7		
Total		60	100.0		

FSTNumberFSTFecalPelletsPoop FST Number FST Fecal Pellets (Poop)

		Frequency	Percent	Valid Percent	Cumulative Percent
Valid	0	2	3.3	3.3	3.3
	1	2	3.3	3.3	6.7
	2	4	6.7	6.7	13.3
	3	6	10.0	10.0	23.3
	4	10	16.7	16.7	40.0
	5	8	13.3	13.3	53.3
	6	8	13.3	13.3	66.7
	7	12	20.0	20.0	86.7
	8	6	10.0	10.0	96.7
	9	2	3.3	3.3	100.0
	Total	60	100.0	100.0	

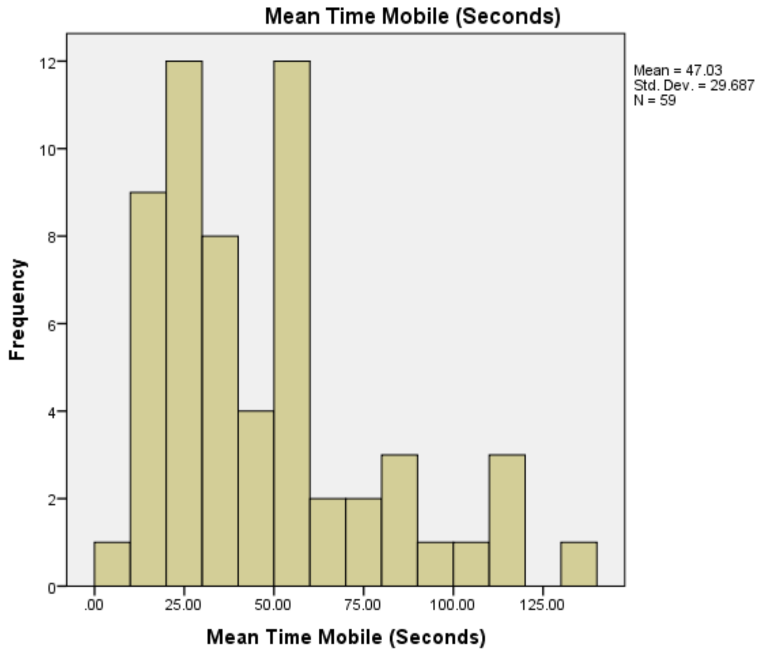

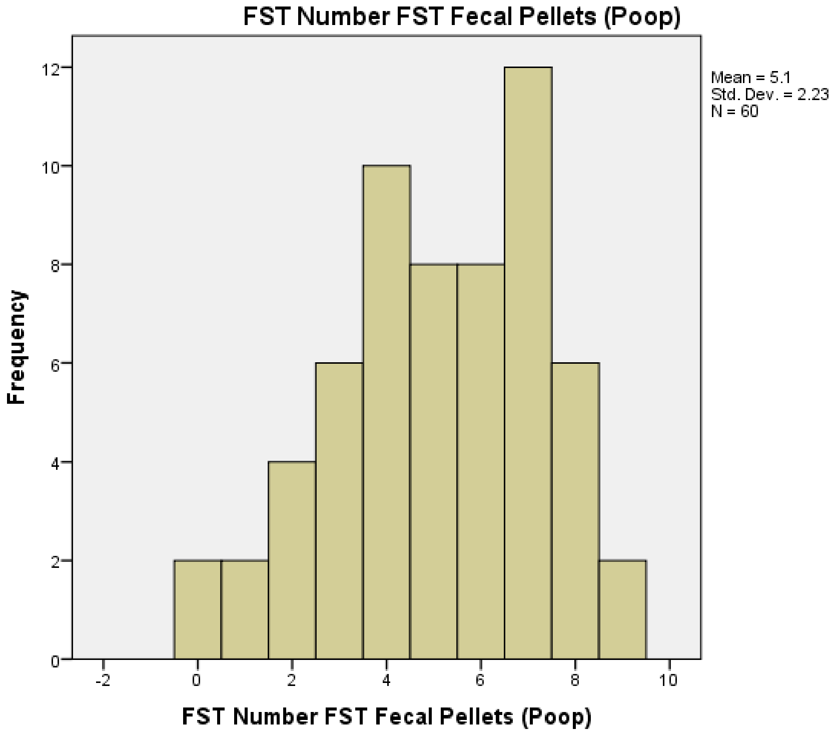

FST Number FST Fecal Pellets (Poop)
Mean = 5.1
Std. Dev. = 2.23
N = 60
Frequency
0
2
4
6
8
10
12
-2
0
2
4
6
8
10
FST Number FST Fecal Pellets (Poop)

Descriptive Statistics: By Group

Statistics[a]

		MeanTimeMobileSeconds Mean Time Mobile (Seconds)	FSTNumberFSTFecalPelletsPoop FST Number FST Fecal Pellets (Poop)
N	Valid	10	10
	Missing	0	0
Mean		43.5333	5.00
Std. Error of Mean		8.63973	.650
Median		41.0000	5.50
Mode		17.00[b]	6
Std. Deviation		27.32123	2.055
Variance		746.449	4.222
Skewness		1.768	-1.729
Std. Error of Skewness		.687	.687
Kurtosis		4.066	3.765
Std. Error of Kurtosis		1.334	1.334
Range		93.67	7
Minimum		17.00	0
Maximum		110.67	7
Sum		435.33	50

a. Group = 1 control-vehicle

b. Multiple modes exist. The smallest value is shown

Statistics[a]

		MeanTimeMobileSeconds Mean Time Mobile (Seconds)	FSTNumberFSTFecalPelletsPoop FST Number FST Fecal Pellets (Poop)
N	Valid	10	10
	Missing	0	0
Mean		36.2667	5.50
Std. Error of Mean		6.61457	.792
Median		28.1667	6.00
Mode		26.33	6
Std. Deviation		20.91712	2.506
Variance		437.526	6.278
Skewness		1.935	-.980
Std. Error of Skewness		.687	.687
Kurtosis		3.537	1.861
Std. Error of Kurtosis		1.334	1.334
Range		69.33	9
Minimum		17.33	0
Maximum		86.67	9
Sum		362.67	55

a. Group = 2 L-Th control drug

Statistics[a]

		MeanTimeMobileSeconds Mean Time Mobile (Seconds)	FSTNumberFSTFecalPelletsPoop FST Number FST Fecal Pellets (Poop)
N	Valid	10	10
	Missing	0	0
Mean		52.2333	5.70
Std. Error of Mean		9.09579	.633
Median		54.1667	6.00
Mode		15.67[b]	7
Std. Deviation		28.76342	2.003
Variance		827.335	4.011
Skewness		.439	-.523
Std. Error of Skewness		.687	.687
Kurtosis		-.210	-.688
Std. Error of Kurtosis		1.334	1.334
Range		90.67	6
Minimum		15.67	2
Maximum		106.33	8
Sum		522.33	57

a. Group = 3 control-naïve

b. Multiple modes exist. The smallest value is shown

Statistics[a]

		MeanTimeMobileSeconds Mean Time Mobile (Seconds)	FSTNumberFSTFecalPelletsPoop FST Number FST Fecal Pellets (Poop)
N	Valid	9	10
	Missing	1	0
Mean		57.9630	5.20
Std. Error of Mean		9.65715	.892
Median		61.3333	5.50
Mode		6.33[b]	3[b]
Std. Deviation		28.97146	2.821
Variance		839.346	7.956
Skewness		-.621	-.143
Std. Error of Skewness		.717	.687
Kurtosis		-.348	-1.563
Std. Error of Kurtosis		1.400	1.334
Range		87.33	8
Minimum		6.33	1
Maximum		93.67	9
Sum		521.67	52

a. Group = 4 PTSD-vehicle

b. Multiple modes exist. The smallest value is shown

Statistics[a]

		MeanTimeMobileSeconds Mean Time Mobile (Seconds)	FSTNumberFSTFecalPelletsPoop FST Number FST Fecal Pellets (Poop)
N	Valid	10	10
	Missing	0	0
Mean		50.2333	4.30
Std. Error of Mean		13.14234	.651
Median		40.3333	4.00
Mode		11.00[b]	3[b]
Std. Deviation		41.55973	2.058
Variance		1727.211	4.233
Skewness		1.232	.262
Std. Error of Skewness		.687	.687
Kurtosis		.460	-.849
Std. Error of Kurtosis		1.334	1.334
Range		120.33	6
Minimum		11.00	1
Maximum		131.33	7
Sum		502.33	43

a. Group = 5 PTSD-drug pre-treatment

b. Multiple modes exist. The smallest value is shown

Statistics[a]

		MeanTimeMobileSeconds Mean Time Mobile (Seconds)	FSTNumberFSTFecalPelletsPoop FST Number FST Fecal Pellets (Poop)
N	Valid	10	10
	Missing	0	0
Mean		43.0333	4.90
Std. Error of Mean		9.34912	.674
Median		35.6667	5.00
Mode		35.67	2[b]
Std. Deviation		29.56451	2.132
Variance		874.060	4.544
Skewness		2.067	-.096
Std. Error of Skewness		.687	.687
Kurtosis		4.965	-1.297
Std. Error of Kurtosis		1.334	1.334
Range		101.33	6
Minimum		16.67	2
Maximum		118.00	8
Sum		430.33	49

a. Group = 6 PTSD-post-treatment

b. Multiple modes exist. The smallest value is shown

One-Way ANOVA

Mean Time Mobile Seconds

Descriptive Statistics

Dependent Variable: MeanTimeMobileSeconds Mean Time Mobile (Se

Group	Mean	Std. Deviation	N
1 control-vehicle	43.5333	27.32123	10
2 L-Th control drug	36.2667	20.91712	10
3 control-naïve	52.2333	28.76342	10
4 PTSD-vehicle	57.9630	28.97146	9
5 PTSD-drug pre-treatment	50.2333	41.55973	10
6 PTSD-post-treatment	43.0333	29.56451	10
Total	47.0282	29.68734	59

Levene's Test of Equality of Error Variances[a]

Dependent Variable: MeanTimeMobileSeconds

F	df1	df2	Sig.
.740	5	53	.597

Tests the null hypothesis that the error variance of the dependent variable is equal across groups.

a. Design: Intercept + Group

Tests of Between-Subjects Effects

Dependent Variable: MeanTimeMobileSeconds Mean Time Mobile (Seconds)

Source	Type III Sum of Squares	df	Mean Square	F	Sig.	Partial Eta Squared
Corrected Model	2889.621[a]	5	577.924	.635	.674	.057
Intercept	131298.392	1	131298.392	144.290	.000	.731
Group	2889.621	5	577.924	.635	.674	.057
Error	48227.999	53	909.962			
Total	181605.333	59				
Corrected Total	51117.620	58				

a. R Squared = .057 (Adjusted R Squared = -.032)

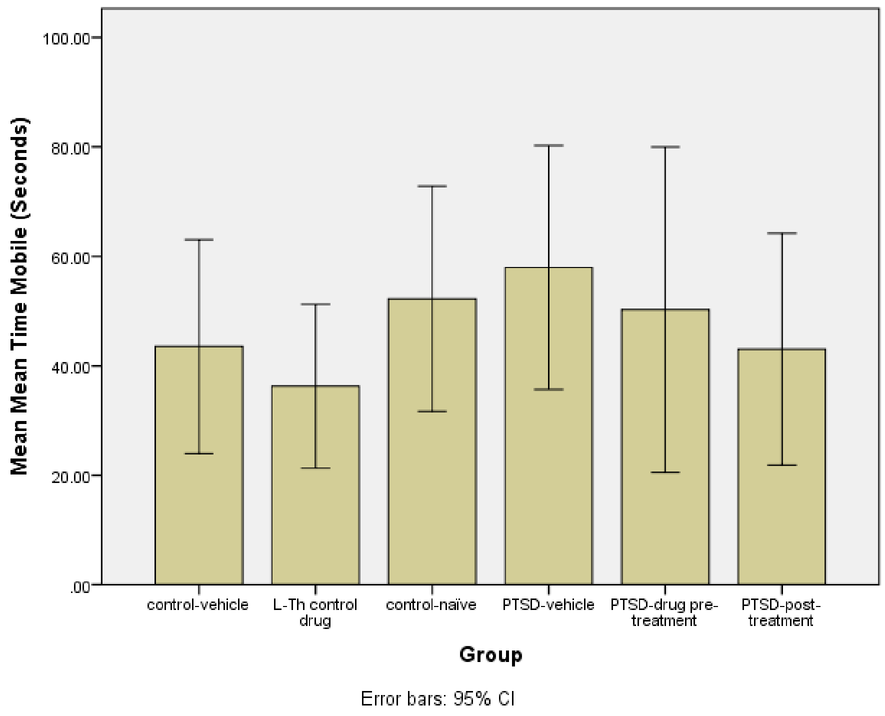

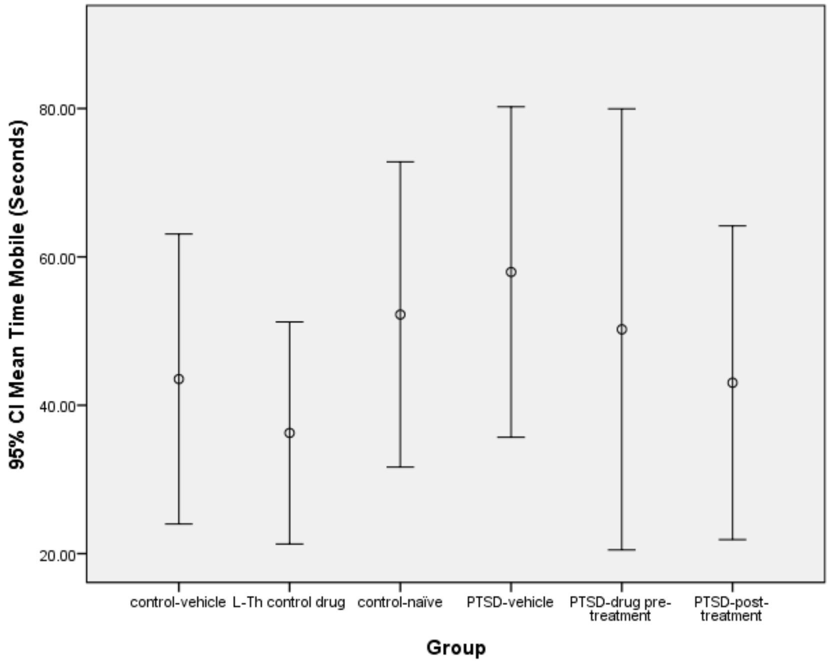

Summary: For the **Mean Time Mobile Seconds** outcome there was not a significant difference between the six groups: $F(5, 53) = .635$, $p = .674$ ($\eta^2 = .057$). Though not significant, the PTSD-vehicle group has the highest mean ($M = 57.96$) and the PGB control drug group the lowest ($M = 36.27$).

Number Fecal Pellets

Descriptive Statistics

Dependent Variable: FSTNumberFSTFecalPelletsPoop FST Number I

Group	Mean	Std. Deviation	N
1 control-vehicle	5.00	2.055	10
2 L-Th control drug	5.50	2.506	10
3 control-naïve	5.70	2.003	10
4 PTSD-vehicle	5.20	2.821	10
5 PTSD-drug pre-treatment	4.30	2.058	10
6 PTSD-post-treatment	4.90	2.132	10
Total	5.10	2.230	60

Levene's Test of Equality of Error Variances[a]

Dependent Variable: FSTNumberFSTFecalPelle

F	df1	df2	Sig.
.702	5	54	.624

Tests the null hypothesis that the error variance of the dependent variable is equal across groups.

a. Design: Intercept + Group

Tests of Between-Subjects Effects

Dependent Variable: FSTNumberFSTFecalPelletsPoop FST Number FST Fecal Pellets (Poop)

Source	Type III Sum of Squares	df	Mean Square	F	Sig.	Partial Eta Squared
Corrected Model	12.200[a]	5	2.440	.469	.798	.042
Intercept	1560.600	1	1560.600	299.688	.000	.847
Group	12.200	5	2.440	.469	.798	.042
Error	281.200	54	5.207			
Total	1854.000	60				
Corrected Total	293.400	59				

a. R Squared = .042 (Adjusted R Squared = -.047)

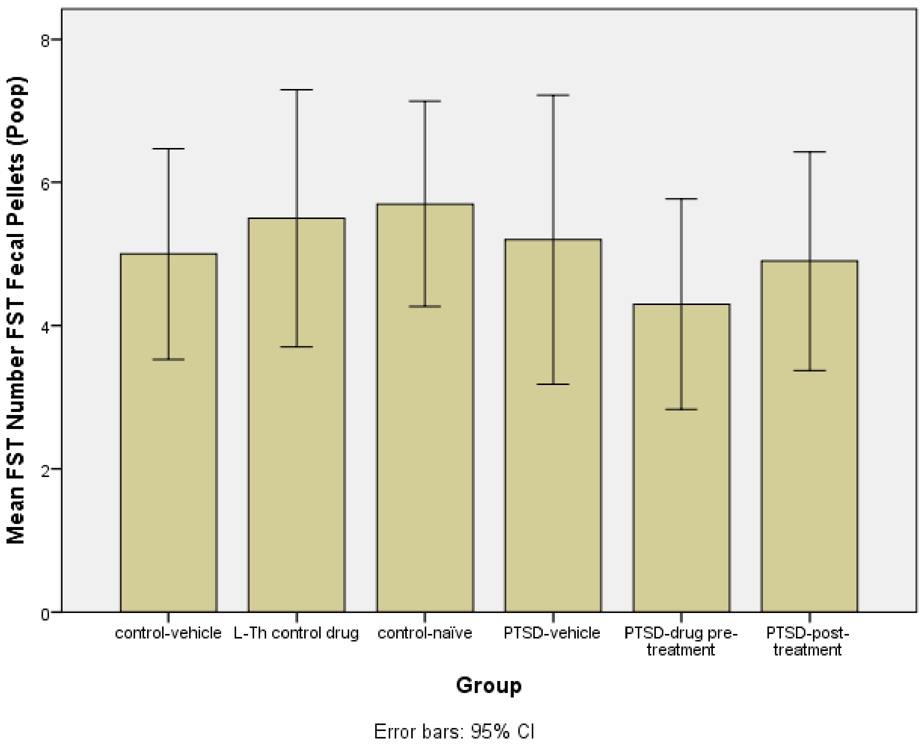

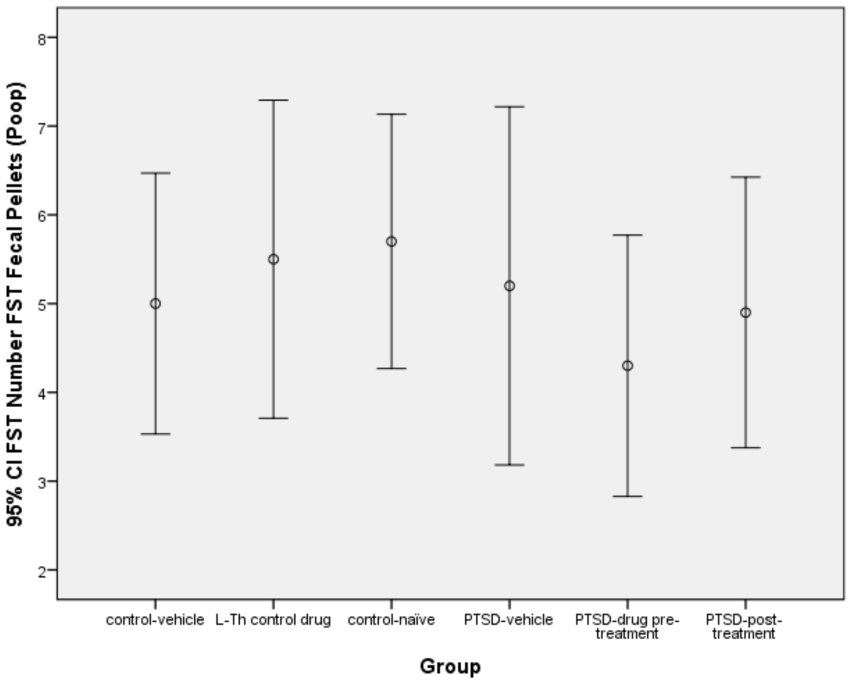

Summary: For the **Number Fecal Pellets** outcome there was not a significant difference between the six groups: $F(5, 54) = .469$, $p = .798$ ($\eta^2 = .042$). Though not significant, the control-naïve group has the highest mean ($M = 5.7$) and the PTSD-drug pre-treatment group the lowest ($M = 4.3$).

Given moderate violation of the normality assumption the Kruskal-Wallis (nonparametric) test was ran, and as with the ANOVA, neither of the results were significant (see table below).

Ranks

	Group	N	Mean Rank
MeanTimeMobileSeconds Mean Time Mobile (Seconds)	1 control-vehicle	10	28.45
	2 L-Th control drug	10	24.90
	3 control-naïve	10	34.05
	4 PTSD-vehicle	9	37.72
	5 PTSD-drug pre-treatment	10	28.40
	6 PTSD-post-treatment	10	27.25
	Total	59	
FSTNumberFSTFecalPelletsPoop FST Number FST Fecal Pellets (Poop)	1 control-vehicle	10	30.00
	2 L-Th control drug	10	34.00
	3 control-naïve	10	35.30
	4 PTSD-vehicle	10	31.50
	5 PTSD-drug pre-treatment	10	23.60
	6 PTSD-post-treatment	10	28.60
	Total	60	

Test Statistics[a,b]

	MeanTimeMobileSeconds Mean Time Mobile (Seconds)	FSTNumberFSTFecalPelletsPoop FST Number FST Fecal Pellets (Poop)
Chi-Square	3.682	2.935
df	5	5
Asymp. Sig.	.596	.710

a. Kruskal Wallis Test

b. Grouping Variable: Group
